# Inhibition of TREM1 attenuates myocardial ischemia-reperfusion injury-induced cardiomyocyte pyroptosis by suppressing the activation of the NF-κB signaling pathway

**DOI:** 10.1371/journal.pone.0340382

**Published:** 2026-01-09

**Authors:** Xiaohui Xu, Liang Cai, Xuan Dang, Jianbin Han, Yan Kou, Chunyan Rong, Junjie Kou

**Affiliations:** 1 Department of Cardiology, The Second Affiliated Hospital of Harbin Medical University, Harbin, China; 2 The Key Laboratory of Myocardial Ischemia, Ministry of Education, Harbin Medical University, Harbin, Heilongjiang Province, China; Noorda College of Osteopathic Medicine, UNITED STATES OF AMERICA

## Abstract

**Background:**

Triggering receptor expressed on myeloid cells-1 (TREM-1) is a potent amplifier of inflammatory responses and plays a critical role in the pathogenesis of various infectious diseases. Notably, emerging evidence suggests that TREM-1 is also involved in the development and progression of cardiovascular diseases, such as atherosclerosis and atrial fibrillation. However, its role in myocardial ischemia/reperfusion (I/R) injury remains unclear. This study aimed to investigate the role of TREM-1 in myocardial I/R injury and to explore the potential underlying molecular mechanisms.

**Methods:**

A hypoxia/reoxygenation (H/R) model was established using HL-1 cardiomyocytes subjected to 6 hours of hypoxia followed by 6 hours of reoxygenation. Pyroptosis levels were assessed by Hoechst-PI staining, lactate dehydrogenase (LDH) release assay, CCK-8 assay, and Western blot analysis. In vivo, a myocardial I/R injury model was established in C57BL/6 mice by subjecting them to 30 minutes of ischemia followed by 24 hours or 7 days of reperfusion. Evaluation was performed using TTC staining, Western blotting, echocardiography, histochemical staining, and immunohistochemistry.

**Results:**

In this study, we found that TREM-1 expression was significantly upregulated in both in vitro and in vivo models of myocardial ischemia-reperfusion injury (MIRI). Pharmacological inhibition of TREM-1 by LR12 effectively reduced the levels of cardiomyocyte pyroptosis and suppressed activation of the NF-κB signaling pathway. In addition, LR12 treatment alleviated myocardial inflammation and fibrosis and improved left ventricular function in mice.Intervention experiments with MCC950, a specific NLRP3 inhibitor, confirmed that NLRP3 inhibition could mimic the anti-pyroptotic effect of LR12 and reduce the expression of pyroptosis-related proteins. Immunofluorescence experiments further verified that inhibition of NF-κB decreased NLRP3 expression, clarifying the association between TREM-1 downstream signals and NLRP3. Long-term follow-up experiments showed that LR12 treatment significantly reduced the area of myocardial fibrosis at 7 days after reperfusion.

**Conclusion:**

Our findings indicate that inhibition of TREM-1 alleviates cardiomyocyte pyroptosis during MIRI by suppressing activation of the NF-κB signaling pathway. Therefore, TREM-1 may represent a promising therapeutic target for the treatment of myocardial ischemia-reperfusion injury.

## 1. Introduction

Myocardial infarction (MI) has been widely recognized as one of the leading causes of disability and mortality worldwide [1]. The application of pharmacological thrombolysis or percutaneous coronary intervention (PCI) can effectively restore blood supply to cardiomyocytes, thereby reducing myocardial injury and limiting the infarct size.However, it is noteworthy that the process of restoring coronary perfusion is often accompanied by a secondary injury phenomenon known as myocardial ischemia-reperfusion injury (MIRI) [2].This condition has been shown not only to compromise the efficacy of therapeutic interventions but also to exacerbate tissue and organ damage [3].Given the severe consequences of MIRI, there is an urgent need to develop novel and effective therapeutic targets.

Pyroptosis is a form of inflammatory programmed cell death characterized by rapid plasma membrane rupture mediated by gasdermin D (GSDMD). Upon recognition of pathogen-associated molecular patterns (PAMPs) or damage-associated molecular patterns (DAMPs) by intracellular pattern recognition receptors, inflammasome assembly is triggered.Specifically, activation of NLRP3 leads to its recruitment of ASC, which in turn facilitates the activation of caspase-1. Activated caspase-1 cleaves full-length GSDMD into its C-terminal and N-terminal fragments. It also processes pro-inflammatory cytokines pro-IL-1β and pro-IL-18 into their mature forms IL-1β and IL-18. The GSDMD N-terminal fragment (GSDMD-N) then forms pores in the plasma membrane, enabling the release of these inflammatory cytokines, which further amplify cellular damage [4,5]. Recent studies have demonstrated that pyroptosis is involved in the pathogenesis of various cardiovascular diseases, including myocardial infarction, myocardial I/R injury, cardiomyopathy, and heart failure [6–9].

Inflammatory activation is a key contributor to the pathogenesis of MIRI [10]. Triggering receptor expressed on myeloid cells-1 (TREM-1), a receptor expressed on myeloid cell surfaces, is a crucial amplifier of inflammatory signaling [11]. Emerging evidence has hghlighted its involvement in multiple cardiovascular diseases, including atherosclerosis, atrial fibrillation, and septic cardiomyopathy [12–14]. TREM-1 has been shown to modulate inflammatory pathways such as NF-κB activation and pyroptosis across various pathological contexts [15]. NF-κB is a central regulator of inflammation [16,17], while pyroptosis represents an inflammation-associated form of programmed cell death [18]. However, the expression pattern of TREM-1 during MIRI and whether the TREM-1/NF-κB signaling axis participates in the regulation of myocardial I/R injury remain unclear.

In this study, we utilized both an in vitro hypoxia/reoxygenation (H/R) cardiomyocyte model and an in vivo ischemia/reperfusion (I/R) mouse model to investigate the role of TREM-1 in MIRI, and further explored the involvement of the TREM-1/NF-κB axis in cardiomyocyte pyroptosis.

## 2. Materials and methods

### 2.1. Construction of the in Vitro H/R model

HL-1 cardiomyocytes were cultured in MEM medium supplemented with 10% fetal bovine serum (FBS) and 1% penicillin streptomycin. Cells were seeded into 6 cm dishes and maintained in a humidified incubator at 37°C with 5% CO2. When cell confluence reached approximately 80%, the culture medium was replaced with glucose- and serum-free MEM. Cells were then transferred to a tri-gas incubator (95% N2, 5% CO2) for 6 hours to induce hypoxia. After hypoxia, the cells were returned to normoxic conditions in standard MEM medium for 6 hours of reoxygenation.The experiment was divided into 4 groups: ① Control group: cultured normally without H/R treatment; ② Control + LR12 (MCE,HY-P3211A) group: cultured normally and pretreated with 5 μmol/L LR12 for 12 hours; ③ H/R group: subjected to H/R treatment alone; ④ H/R + LR12 group: pretreated with 5 μmol/L LR12 for 12 hours before H/R treatment. Additionally, an H/R + PDTC (MCE,HY-18738) group was set up: pretreated with 20 μmol/L PDTC for 12 hours before H/R treatment to verify the role of the NF-κB pathway; and an H/R + MCC950 (MCE,HY-12815) group: pretreated with 10 μmol/L MCC950 for 2 hours before H/R treatment to verify the effect of NLRP3 on pyroptosis.

### 2.2. CCK-8 assay

HL-1 cells were seeded in 96-well plates at a density of 5 × 10^3^ cells per well, with 5 replicate wells per group. PBS was added around the plate to prevent evaporation. After culturing for 12 hours to allow cell adhesion, the cells were treated according to the above groups. At the end of treatment, 100 μL of serum-free MEM medium containing 10% CCK-8 reagent (Biosharp, catalog number: ALX-850–039) was added to each well, followed by incubation at 37°C in the dark for 1 hour. The absorbance (OD value) at 450 nm was measured using a microplate reader (purchased from Tacan Company, Switzerland). Cell viability was calculated as follows: Cell viability = [(OD value of experimental group – OD value of blank control group)/ (OD value of control group – OD value of blank control group)] × 100%.

### 2.3. LDH activity assay

After the treatment of cells in each group, the culture supernatants were collected and assayed according to the instructions of the LDH detection kit (Beyotime Biotechnology, Shanghai, catalog number: C0017): 120 μL of the supernatant was mixed with 60 μL of the detection reagent, incubated at 37°C in the dark for 30 minutes, and the OD value at 450 nm was measured using a microplate reader (purchased from Tacan Company, Switzerland). The LDH release rate was calculated as follows: LDH release rate = [(OD value of experimental group – OD value of blank group)/ (OD value of control group – OD value of blank group)] × 100%.

### 2.4. Hoechst 33342/PI staining

HL-1 cells were seeded into 24-well plates and cultured overnight. After treatment, cells were stained with Hoechst 33342 (1:1000) for 30 minutes in the dark, followed by fixation with paraformaldehyde for 15 minutes. Propidium iodide (PI) solution (1:1000) was then added and incubated for 5 minutes. The cells were washed with PBS and observed under a fluorescence microscope.Observation was performed under a fluorescence microscope (purchased from Leika Company, Germany): Hoechst 33342 stained cell nuclei blue, while PI stained cells with damaged cell membranes (pyroptotic/necrotic cells) red.

### 2.5. In vivo myocardial ischemia/reperfusion (I/R) model

Eight-week-old male C57BL/6 mice were purchased from Harbin Medical University. A myocardial ischemia-reperfusion injury model was established by opening the chest through a small left incision to expose the heart, ligating the left anterior descending coronary artery for 30 minutes, followed by 24 hours of reperfusion. The mice were divided into 3 groups: sham operation control group, ischemia-reperfusion (I/R) group, and I/R + LR12 treatment group. The LR12 treatment regimen was as follows: 5 mg/kg LR12 was dissolved in 100 μL normal saline, intraperitoneally injected once every 12 hours for a total of 2 times before modeling, and an additional injection was administered 5 minutes before reperfusion; during reperfusion, injections were given once every 12 hours until the mice were euthanized. The animal experiments in this study were approved by the Ethics Committee of the Second Affiliated Hospital of Harbin Medical University (approval number: YJSDW2024−040), and all experimental operations strictly followed the Guidelines for Ethical Review of Experimental Animals (GB/T 35892−2018) and the requirements of PLOS ONE regarding animal humane endpoints and welfare.

#### 2.5.1. Preoperative care and anesthesia optimization for mice.

Mice were fasted for 12 hours before surgery with free access to water. During fasting, the breeding environment was maintained at 22 ± 1°C, relative humidity of 50 ± 5%, and a light cycle of 12 hours light/12 hours dark to reduce environmental stress. Anesthesia was induced by intraperitoneal injection of 5% sodium pentobarbital (50 mg/kg). After injection, the corneal reflex of mice and the limb response to painful stimuli (such as tail clamping) were closely monitored to ensure the anesthetic depth reached the standard of “disappeared corneal reflex and no spontaneous limb movement”, avoiding intraoperative pain caused by insufficient anesthesia. If the anesthetic effect was poor (such as limb twitching during surgery), 1/4 of the initial dose of sodium pentobarbital was supplemented to maintain stable intraoperative anesthesia. A rodent-specific ventilator was used for assisted ventilation (respiratory rate: 60 breaths per minute, tidal volume: 10 mL/kg). During ventilation, a body temperature maintenance instrument (Chengdu TaiMeng Technology Co., Ltd., model: TP-2000) was used to maintain the mouse body temperature at 37 ± 0.5°C to prevent stress injury caused by hypothermia.

#### 2.5.2. Definition and monitoring of humane endpoints.

The following humane endpoints were predefined in this study. If any of the conditions occurred, euthanasia was performed immediately to avoid excessive pain in mice: inability to eat or drink independently for more than 1 hour during the postoperative recovery period, with a weight loss of more than 15% within 24 hours; severe respiratory abnormalities (respiratory rate > 100 breaths per minute or < 30 breaths per minute), accompanied by cyanosis (cyanosis of oral/nasal mucosa) or persistent convulsions; limb paralysis, inability to move independently, or no response to external stimuli (such as touch, sound); persistent hypotension after myocardial ischemia-reperfusion (non-invasive tail artery sphygmomanometer showed systolic blood pressure < 60 mmHg for more than 30 minutes). During the experiment, the mental state, activity ability, food and water intake, and body weight of mice were monitored daily and recorded in the Experimental Animal Health Monitoring Form. If abnormalities occurred, timely assessment was conducted to determine whether the humane endpoint was reached.

#### 2.5.3. Mouse euthanasia methods and pain relief measures.

At the end of the experiment (24 hours after reperfusion), mice were euthanized by intraperitoneal injection of an excessive dose of sodium pentobarbital (200 mg/kg, 4 times the anesthetic dose). After injection, the corneal reflex, breathing, and heartbeat of mice were monitored. Euthanasia was confirmed successful when breathing and heartbeat stopped with no signs of recovery for 5 consecutive minutes. This method can rapidly inhibit the central nervous system and avoid pain during death. Violent operations were avoided during euthanasia, and mice were gently fixed to reduce limb compression and struggle. After euthanasia, blood was collected from the heart to confirm the cessation of the circulatory system and ensure no possibility of resuscitation, in line with the principle of “painless euthanasia”.

#### 2.5.4. Postoperative care measures.

After surgery, mice were placed in a 37°C constant temperature recovery box. After the recovery of spontaneous breathing and corneal reflex, they were transferred to clean cages for individual housing (to avoid stress caused by fighting during group housing). Soft bedding was laid in the cages, and adequate sterile water and easily digestible feed (such as moistened mouse feed) were provided. The recovery status of mice was monitored every hour within 6 hours after surgery, recording the awakening time, activity status, and wound bleeding; within 24 hours after surgery, monitoring was conducted every 4 hours to observe whether the wound was infected (such as redness, swelling, suppuration). If wound abnormalities occurred, antibiotic ointment (such as erythromycin ointment) was applied for anti-infection treatment.

### 2.6. Echocardiography

Transthoracic echocardiography was used to assess cardiac function. Mice were anesthetized with pentobarbital sodium, and M-mode echocardiographic imaging was performed using a Vevo 3100LT system.

### 2.7. TTC staining

Twenty-four hours after reperfusion, the mice were euthanized, and the hearts were rapidly excised, rinsed with normal saline, and then frozen on dry ice. The hearts were cut into 5 slices of uniform thickness (approximately 1 mm) along the short axis using a cardiac sectioning mold, placed in 2% TTC solution (Solarbio, Beijing, catalog number: G3005), and incubated at 37°C in the dark for 30 minutes. Normal myocardial tissue appeared red, while infarcted myocardial tissue appeared white. Image J software was used to analyze the infarct area and ventricular area of each slice, and the percentage of infarct area relative to the left ventricular area was calculated as follows: Infarct area (%) = (Infarct area/ Left ventricular area) × 100%.

### 2.8. Histological staining for observing myocardial pathological changes

#### 2.8.1. HE staining of cardiac tissue (Observation of Myocardial Pathological Morphology).

Fresh cardiac tissue (below the ligation line) was collected and fixed in 4% paraformaldehyde solution at 4°C for 36 hours. After fixation, the cardiac tissue was gradiently dehydrated using a tissue dehydrator and embedded in paraffin to form paraffin blocks. The paraffin blocks were sectioned into 5 μm-thick serial sections using a microtome, which were then attached to glass slides and baked in a 60°C oven for 2 hours to ensure firm adhesion for subsequent use. Staining was performed following the instructions of the modified Hematoxylin-Eosin (HE) Staining Kit (Solarbio, Beijing, Catalog Number: G1120). ① Deparaffinization: Sections were deparaffinized in Xylene I and Xylene II for 10 minutes each, then rehydrated through a graded ethanol series to distilled water. ② Staining: Sections were stained with hematoxylin solution for 10 minutes, rinsed with tap water, differentiated with 1% hydrochloric acid-ethanol for 30 seconds, blued with bluing solution for several seconds, and further blued with warm water for 10 minutes. Subsequently, sections were stained with eosin solution for 1 minute and briefly rinsed with distilled water. ③ Dehydration and clearing: Gradient dehydration was performed with 95% and 100% ethanol, followed by clearing in xylene, and finally mounted with neutral balsam. After the mounted sections dried, the glass slides were scanned using the Slide Scanning System SQS-12P for subsequent analysis of myocardial pathological morphological changes.

#### 2.8.2. Masson’s trichrome staining of cardiac tissue (Detection of Myocardial Fibrosis).

Tissue fixation, embedding, and sectioning were performed identically to HE staining. Staining was conducted according to the instructions of the modified Masson’s Trichrome Staining Kit (Solarbio, Beijing, Catalog Number: Cat#G1346). ① Deparaffinization to water: Sections were deparaffinized to distilled water following the same steps as HE staining. Enzyme staining was then performed: incubation in an enzyme solution in a 60°C oven for 1 hour, followed by three rinses with distilled water (5 minutes each). ② Nuclear staining: Staining with celestine blue for 3 minutes, rinsed twice with distilled water; staining with hematoxylin for 3 minutes, rinsed twice with distilled water; differentiated with acid differentiation solution for 30 seconds, briefly rinsed with distilled water, and blued under running water for 10 minutes. ③ Primary staining of cytoplasm and collagen: Acid fuchsin-ponceau solution was added to completely cover the tissue and incubated at room temperature for 5 minutes. Rapidly rinsed twice with distilled water (30 seconds each) to remove residual surface staining solution. ④ Differentiation and counterstaining of collagen: Sections were immersed in phosphomolybdic acid solution and incubated at room temperature for 10 minutes. The supernatant was poured off, and aniline blue staining solution was directly added to cover the tissue, followed by incubation at room temperature for 5 minutes. ⑤ Differentiation, dehydration, and clearing: Sections were immersed in glacial acetic acid solution for 1 minute, then directly transferred to 95% ethanol and 100% ethanol for dehydration, finally cleared in xylene and mounted with neutral balsam. After mounting, sections were air-dried in a well-ventilated area and scanned using the Slide Scanning System SQS-12P. Myocardial muscle fibers appeared bright red, collagen fibers appeared dark blue, and cell nuclei appeared blue-black. The degree of fibrosis was evaluated by quantitative analysis of “collagen fiber area/ total myocardial area × 100%” using Image J software.

#### 2.8.3. Immunohistochemical staining of cardiac tissue.

Tissue fixation, embedding, and sectioning were performed as described in the HE staining protocol. Immunohistochemical staining was carried out following the instructions of the Immunohistochemistry Kit (Boster Biological Technology, Wuhan, Catalog Number: PK10006). ① Routine deparaffinization to water as per HE staining. ② Antigen retrieval: Antigen retrieval was performed using antigen retrieval solution (citrate buffer, pH = 6.0) in an antigen retrieval pot. After cooling, sections were rinsed three times with PBS (5 minutes each). ③ Blocking of endogenous peroxidase: Incubation with 3% hydrogen peroxide solution in a humid chamber at room temperature for 20 minutes, followed by three PBS rinses (5 minutes each). ④ Blocking: Incubation with blocking solution (5% BSA) at room temperature for 1 hour. ⑤ Primary antibody incubation: Incubation with primary antibody against TREM1 (1:150, affinity, DF6091) at 4°C overnight. The next day, sections were rinsed three times with PBS (5 minutes each), then incubated with secondary antibody at room temperature for 2 hours, followed by three PBS rinses (5 minutes each). ⑥ DAB color development, hematoxylin staining (counterstaining of cell nuclei), and mounting with neutral balsam. Image acquisition and result analysis were performed identically to HE staining. Result interpretation: Cell nuclei appeared blue, and regions with positive expression of the target protein appeared brownish-yellow. The relative expression level of the target protein was subsequently evaluated by quantitative analysis of “integrated optical density (IOD) of positive regions/ tissue area” using Image J software.

### 2.9. Confocal detection of NF-κB nuclear translocation

HL-1 cells were seeded on cell climbing slides and treated according to groups (Control group, Control + LR12 group, H/R group, H/R + LR12 group). After treatment, cells were fixed with 4% paraformaldehyde for 15 minutes, permeabilized with 0.1% Triton X-100 for 15 minutes, and blocked with 5% BSA for 30 minutes. Primary antibody against NF-κB p65 (Abmart, T55034F) was added at a dilution of 1:100 and incubated at 4°C overnight. Fluorescent secondary antibody (Abcam, ab150080) was added at a dilution of 1:500 and incubated at room temperature for 1 hour. Nuclei were stained with DAPI (Beyotime, C0017) for 5 minutes. Observation was performed under a confocal microscope, and the nuclear fluorescence intensity in different groups was counted and analyzed.

### 2.10. NLRP3 immunofluorescence assay

HL-1 cells were seeded on cell climbing slides and divided into four groups: Control group, Control + PDTC group, H/R group, and H/R + PDTC group. After treatment, the same confocal procedure as described above was performed. Primary antibody against NLRP3 (Abways, CY5651) was added at a dilution of 1:100, followed by the corresponding fluorescent secondary antibody (Abcam, ab150080) at a dilution of 1:500. The expression level and localization of NLRP3 were observed under a fluorescence microscope, and the fluorescence intensity was quantified using Image J software.

### 2.11. Western blot assay

Myocardial tissue or cells were collected and lysed on ice with RIPA lysis buffer containing protease inhibitors, followed by sonication. The lysate was centrifuged at 12,000 rpm at 4°C for 15 minutes, and the supernatant was collected. Protein concentration was determined using the BCA method. A total of 30 μg of protein was separated by 12.5% SDS-PAGE and transferred to PVDF membranes. The membranes were blocked with 5% BSA for 2 hours and incubated with primary antibodies at 4°C overnight. After washing 3 times with TBST (10 minutes each), the membranes were incubated with secondary antibodies for 1 hour, followed by another 3 washes with TBST (10 minutes each). Protein bands were visualized using Meilun ECL reagent, and images were acquired with a Tanon imaging system. The gray value of bands was measured using Image J software, and the relative expression level of target proteins was calculated with tubulin as the internal reference. The antibodies used were as follows: TREM1 (affinity, Cat. No.: DF6091, Dilution: 1:1000), NLRP3 (Abways, Cat. No.: CY5651, Dilution: 1:1000), GSDMD (Abmart, Cat. No.: PU224937, Dilution: 1:2000), caspase-1 (Abmart, Cat. No.: T510200, Dilution: 1:1500), IL-1β (Abmart, Cat. No.: P50520, Dilution: 1:1500), IL-18 (Abmart, Cat. No.: M027287, Dilution: 1:1500), P-IκBα (Abmart, Cat. No.: TP56280, Dilution: 1:1000), IκBα (Abmart, Cat. No.: T55026, Dilution: 1:1000), P-NF-κB (Abmart, Cat. No.: TP56372, Dilution: 1:1000), NF-κB (Abmart, Cat. No.: T55034, Dilution: 1:1000), and tubulin (Abways, Cat. No.: AB0050, Dilution: 1:5000).

### 2.12. Data and statistical analysis

All experiments were repeated three times or more. Experimental data were expressed as mean ± standard error of the mean (mean ± SEM). Independent samples t-test was used for comparisons between two groups, and one-way analysis of variance (one-way ANOVA) was used for comparisons among multiple groups. Statistical analysis and graphing were performed using GraphPad Prism 9.0 software. Fluorescence intensity and the exposure intensity of target proteins were analyzed using Image J software. ns indicated no statistical significance, and P < 0.05 was considered statistically significant (*P < 0.05; **P < 0.01; ***P < 0.001; ****P < 0.0001).

## 3. Results

### 3.1. Hypoxia/Reoxygenation induces cardiomyocyte pyroptosis and upregulates TREM-1 expression

**Fig 1:**
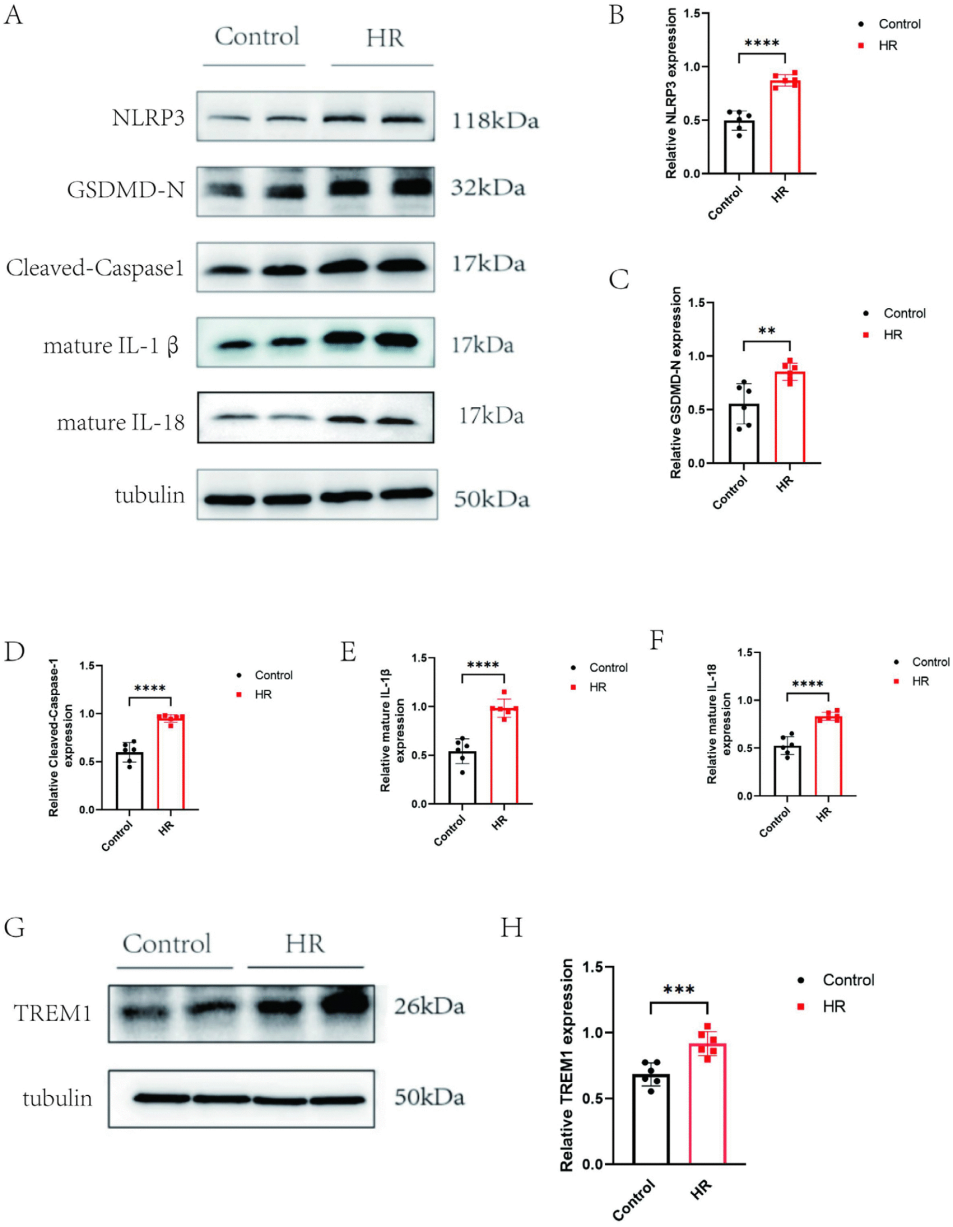
H/R Induces Cardiomyocyte Pyroptosis and Upregulates TREM-1 Expression (A-F) WB method was used to detect the expression of pyroptosis related proteins NLRP3, GSDMD-N, Cleaved-Caspase-1, and mature IL-1β (IL-1β)、mature IL-18 (G-H) WB method was used to detect the expression of TREM1. **P < 0.01,***P < 0.001,****P < 0.0001.

Previous studies have demonstrated that hypoxia/reoxygenation (H/R) can induce cardiomyocyte pyroptosis [19,20]. To validate this, Western blot analysis was performed. The results showed that the expression levels of pyroptosis-related proteins including NLRP3, GSDMD-N, Cleaved-Caspase-1, and mature interleukin-1β (IL-1β)、mature IL-18 were significantly increased following H/R treatment (P < 0.01; Fig 1A–1F). Furthermore, Western blotting confirmed that TREM-1 protein expression was also markedly upregulated after H/R (P < 0.001; Fig 1G–1H).

### 3.2. Inhibition of TREM-1 by LR12 attenuates cardiomyocyte pyroptosis

**Fig 2:**
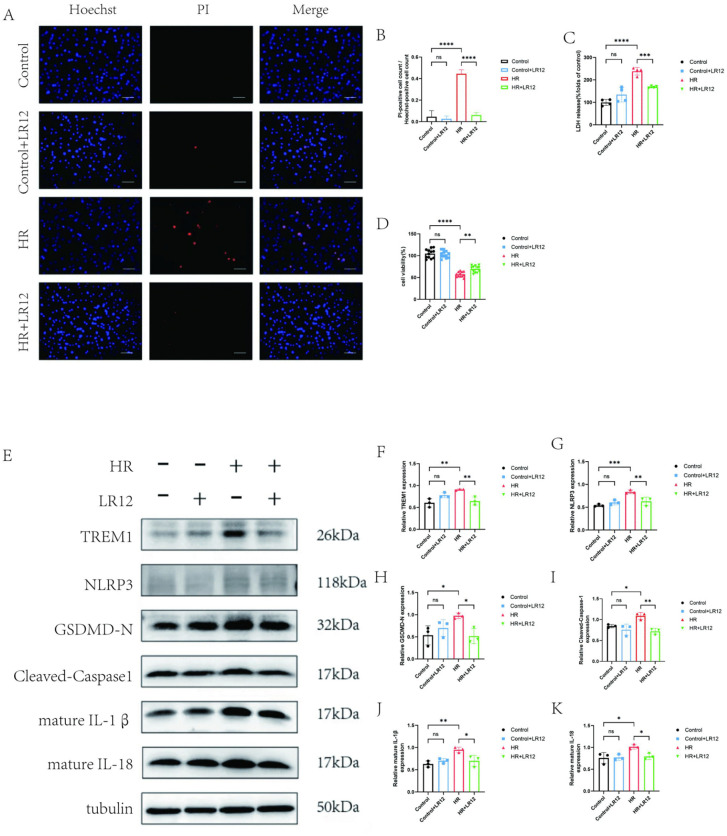
Inhibition of TREM-1 by LR12 Attenuates Cardiomyocyte Pyroptosis. After different treatments **(A-B)**, Hoechst PI was used to detect cell pyroptosis. (scale bar: 100 μm). **(C)** LDH release assay for detecting LDH activity in cell supernatant **(D)** CCK8 detects cell viability in different groups **(E-K)** WB method was used to detect the expression of pyroptosis related proteins TREM1,NLRP3, GSDMD-N, Cleaved-Caspase-1, and mature IL-1β (IL-1β)、mature IL-18.ns indicates no statistical significance*P < 0.05,**P < 0.01,***P < 0.001,****P < 0.0001.

To clarify the role of TREM1 in H/R-induced cardiomyocyte pyroptosis, LR12, a specific TREM1 inhibitor, was introduced for intervention. The experiment was divided into four groups: Control group, Control + LR12 group, H/R group, and H/R + LR12 group. The results of Hoechst 33342/PI double staining (Fig 2A-2B; Hoechst stains viable cell nuclei blue, while PI stains dead cell nuclei red) showed that the number of pyroptotic cells in the H/R group was significantly higher than that in the Control group, whereas LR12 intervention markedly reduced the number of pyroptotic cells. Detection of LDH activity in cell supernatants (Fig 2C) and CCK-8 assay (Fig 2D) further confirmed that LR12 significantly decreased H/R-induced LDH release and improved cell viability (P < 0.05). Western blot results demonstrated that H/R treatment significantly upregulated the expression of NLRP3, GSDMD-N, Cleaved-Caspase-1, mature IL-1β, and mature IL-18 (P < 0.05), while LR12 intervention effectively inhibited the abnormal upregulation of these proteins (P < 0.05; Fig 2E-2K). These findings indicate that LR12 can effectively alleviate H/R-induced cardiomyocyte pyroptosis by inhibiting TREM1.

### 3.3. Inhibition of TREM-1 can alleviate H/R-induced pyroptosis by downregulating NF-κB activation

**Fig 3:**
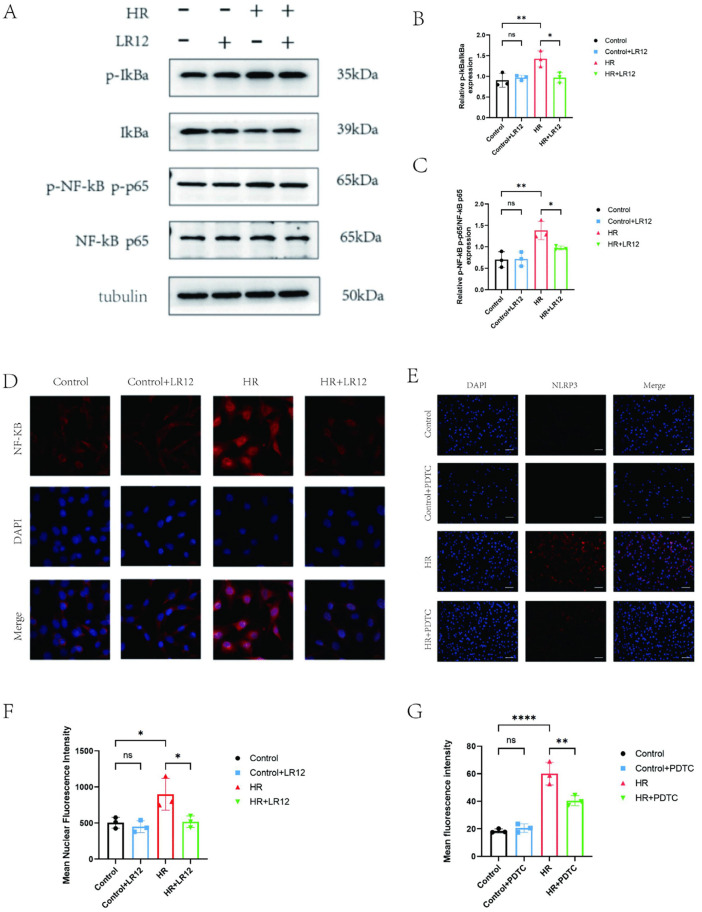
Inhibition of TREM-1 can alleviate H/R-induced pyroptosis by downregulating NF-κB activation. The (A-C) WB method was used to detect the expression of NF-κB signaling pathway related proteins p-IkBa, IkBa, p-NF-κB, and NF-κB. (D-E) Confocal microscopy for observing NF-κB nuclear translocation. (scale bar: 20 μm). (F-G) NLRP3 fluorescence assay for observing NLRP3 expression in different groups. (scale bar: 100 μm).ns indicates no statistical significance,*P < 0.05,**P < 0.01.

Previous studies have confirmed that TREM1 is involved in NF-κB-mediated inflammatory responses under various pathological conditions [21–23]. Western blot analysis showed that H/R treatment significantly increased the ratios of p-IκBα/IκBα and p-NF-κB/NF-κB (P < 0.05), while LR12 intervention markedly reversed this effect (Fig 3A-3C). Results of confocal detection for NF-κB nuclear translocation demonstrated that H/R treatment significantly increased the proportion of cells with nuclear p65 positivity, and LR12 intervention obviously attenuated the nuclear translocation(P < 0.05) (Fig 3D-3E), confirming that TREM1 function may depend on NF-κB signaling activation. Further NLRP3 immunofluorescence experiments showed that treatment with PDTC, an NF-κB inhibitor, reduced the H/R-induced upregulation of NLRP3 expression (P < 0.01) (Fig 3F-3G), clarifying the regulatory association between TREM1 downstream NF-κB signaling and NLRP3. In addition, in the absence of H/R stimulation, there was no significant difference in NF-κB activity between the LR12 treatment group and the control group, suggesting that LR12 has no off-target effect on NF-κB and further verifying the specificity of the TREM1-NF-κB signaling axis.

### 3.4. Inhibition of NF-κB or NLRP3 effectively attenuates cardiomyocyte pyroptosis

**Fig 4:**
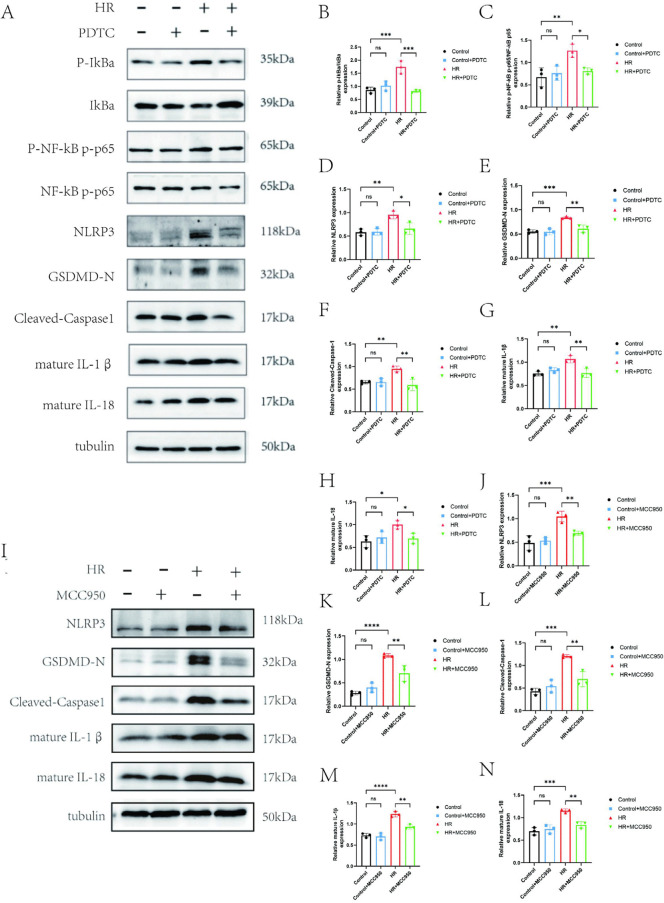
Inhibition of NF-κB or NLRP3 Effectively Attenuates Cardiomyocyte Pyroptosis. **(A-H)** WB method was used to detect the expression of NF-κB signaling pathway related proteins p-IkBa, IkBa, p-NF-κB, and NF-κB and the expression of pyroptosis related proteins NLRP3、GSDMD-N、 Cleaved-Caspase-1, and mature IL-1β (IL-1β)、mature IL-18. (I-N) WB method was used to detect the expression of pyroptosis-related proteins after inhibiting NLRP3 with MCC950.ns indicates no statistical significance,*P < 0.05,**P < 0.01,***P < 0.001.

To verify the mediating role of NF-κB signaling in H/R-induced cardiomyocyte pyroptosis, cardiomyocytes were pretreated with PDTC, a specific NF-κB inhibitor, before H/R exposure. Western blot results showed that compared with the control group, H/R treatment significantly increased the ratios of p-IκBα/IκBα and p-NF-κB/NF-κB (P < 0.05), and simultaneously upregulated the expressions of NLRP3, GSDMD-N, Cleaved-Caspase-1, mature IL-1β, and mature IL-18 (P < 0.05). However, PDTC pretreatment significantly inhibited the above H/R-induced effects (Fig 4A-4H), indicating that H/R triggers cardiomyocyte pyroptosis mainly by activating the NF-κB pathway. Intervention experiments with MCC950, a specific NLRP3 inhibitor, demonstrated that pretreatment with 10 μM MCC950 for 2 hours significantly reduced the H/R-induced expression levels of Cleaved-Caspase-1, mature IL-1β, mature IL-18, and GSDMD-N (P < 0.01)(Fig 4I-4N). This directly confirms that NLRP3 is a key molecule in H/R-induced cardiomyocyte pyroptosis, and inhibition of NLRP3 can mimic the anti-pyroptotic effect of LR12, further supporting the core role of the TREM1-NF-κB-NLRP3 pathway in regulating cardiomyocyte pyroptosis.

### 3.5. TREM-1 inhibition attenuates myocardial pyroptosis in vivo via NF-κB suppression

**Fig 5:**
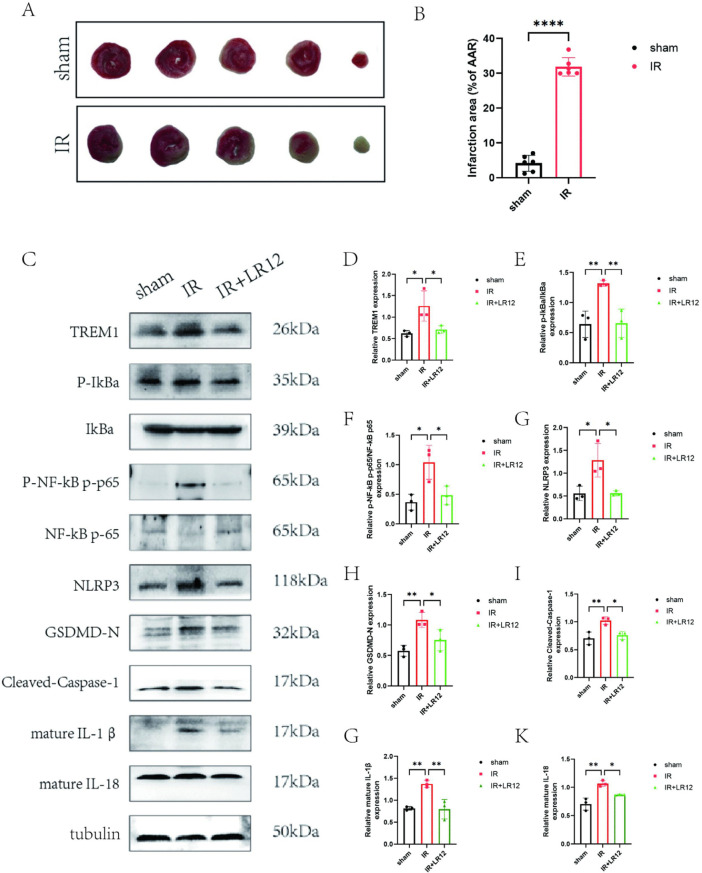
TREM-1 Inhibition Attenuates Myocardial Pyroptosis In Vivo via NF-κB Suppression. Comparison of TTC staining between (A-B) sham group and MIRI group mice. (C-K) WB method was used to detect the expression ofTREM1and NF-κB signaling pathway related proteins p-IkBa, IkBa, p-NF-κB,NF-κB and the expression of pyroptosis related proteinsNLRP3、GSDMD-N、 Cleaved-Caspase-1, and mature IL-1β (IL-1β)、mature IL-18.ns indicates no statistical significance,*P < 0.05,**P < 0.01,****P < 0.0001.

To validate the above findings in vivo, a myocardial ischemia/reperfusion (I/R) model was established in 8-week-old male C57BL/6 mice by ligating the left anterior descending (LAD) coronary artery for 30 minutes followed by 24 hours of reperfusion. Mice were divided into sham, I/R, and I/R + LR12 groups. TTC staining revealed a significantly increased infarct size in the I/R group compared to the sham group (P < 0.0001; Fig 5A–5B), confirming successful model establishment. Western blot analysis demonstrated that the expression of TREM-1, p-IκBα/IκBα, p-NF-κB/NF-κB, NLRP3, GSDMD-N, Cleaved-Caspase-1,mature IL-1β, and mature IL-18 were significantly upregulated in the I/R group (P < 0.05), and these increases were attenuated by LR12 treatment (Fig 5C–5K). This indicates that in vivo, inhibition of TREM1 can also attenuate cardiomyocyte pyroptosis induced by myocardial ischemia-reperfusion injury by blocking the activation of the NF-κB signaling pathway.

### 3.6. TREM-1 inhibition improves prognosis in mice with MIRI

**Fig 6:**
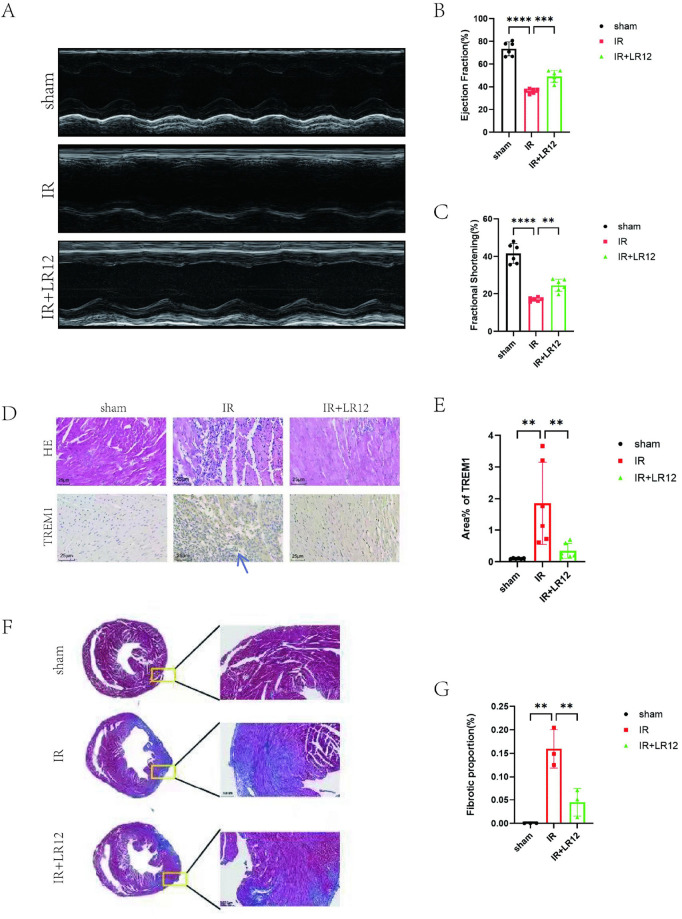
TREM-1 Inhibition Improves Prognosis in Mice with MIRI. **(A-C)** Measure the cardiac function of mice after corresponding treatment measures using echocardiography. **(D-E)** H&E and immunohistochemistry was used to detect TREM1 expression in different groups. (scale bar: 25 μm). (F-G) Masson staining results of different groups. (scale bar: 100 μm).**P < 0.01,***P < 0.001,****P < 0.0001. Abbreviation: TREM-1, Triggering Receptor Expressed on Myeloid Cells-1; NF-κB, Nuclear Factor-κB; NLRP3, NOD-like Receptor Pyrin Domain-Containing Protein 3; H/R/HR, Hypoxia/Reoxygenation; MIRI, Myocardial Ischemia-Reperfusion Injury; I/R/IR, Ischemia-Reperfusion Injury.

Echocardiographic examination showed that left ventricular function was significantly impaired in MIRI mice. Compared with the sham operation group, the left ventricular ejection fraction (LVEF) and fractional shortening (FS) in the I/R group were significantly decreased (P < 0.001), indicating impaired cardiac systolic function; LR12 treatment significantly reversed these parameters and restored cardiac systolic function (Fig 6A-C). HE and immunohistochemical analyses (Fig 6D-E) demonstrated that compared with the sham operation group, the I/R injury group exhibited obvious cardiomyocyte hypertrophy, disordered and sparse arrangement of myocardial fibers, destruction of normal structure, interstitial edema, inflammatory cell infiltration, and significantly increased TREM1 expression(P < 0.01). However, LR12 pretreatment reduced TREM1 expression and alleviated these pathological changes. Masson’s trichrome staining during long-term follow-up (7 days after reperfusion) showed extensive myocardial fibrosis in the I/R group, and LR12 treatment significantly reduced the fibrotic area(P < 0.01)(Fig 6F-G). These results indicate that inhibition of TREM1 can not only improve the short-term cardiac function of MIRI mice but also reduce long-term myocardial fibrosis and improve the prognosis of myocardial remodeling.

## 4. Discussion

Myocardial ischemia-reperfusion injury (MIRI) is an inevitable pathological process in clinical revascularization therapy for myocardial infarction. Its core mechanism is closely related to the excessive activation of inflammatory responses and abnormal cardiomyocyte death, which seriously affects treatment outcomes and patient prognosis. Through in vitro hypoxia-reoxygenation (H/R) model of HL-1 cardiomyocytes and in vivo myocardial ischemia-reperfusion (I/R) model of C57BL/6 mice, this study systematically explored the role and molecular mechanism of triggering receptor expressed on myeloid cells-1 (TREM1) in MIRI. It is the first to clarify the key regulatory role of the TREM1-NF-κB-NLRP3 signaling axis in cardiomyocyte pyroptosis, providing new experimental evidence and theoretical support for the targeted therapy of MIRI.

As an important amplifier of inflammatory responses, the role of TREM1 in infectious diseases and cardiovascular diseases has received extensive attention [21,23–29]. This study found that the protein expression level of TREM1 was significantly upregulated in both in vitro H/R and in vivo I/R models, and was positively correlated with the degree of cardiomyocyte pyroptosis. This result is consistent with the abnormal expression characteristics of TREM1 in cardiovascular diseases such as atherosclerosis and atrial fibrillation reported in previous studies [26,27], suggesting that TREM1 may act as a key regulatory molecule in the pathological process of MIRI, participating in the inflammatory cascade reaction and cell death regulation after myocardial ischemia-reperfusion. Notably, through intervention with LR12, a specific TREM1 inhibitor, this study found that blocking TREM1 can not only significantly reduce H/R/I/R-induced cardiomyocyte pyroptosis, but also improve cardiac systolic function, alleviate myocardial inflammatory infiltration and long-term fibrosis in mice. This finding further confirms that the abnormal activation of TREM1 plays an important role in promoting pathological damage in MIRI, and it may participate in the regulation of cardiomyocyte survival and tissue repair by regulating downstream signaling pathways, providing a new potential target for the treatment of MIRI.

Pyroptosis, an inflammatory-related programmed cell death mode, is characterized by GSDMD-N-mediated cell membrane perforation and the release of inflammatory factors such as IL-1β and IL-18, which plays a key role in the pathological process of MIRI [4,5]. This study confirmed that H/R/I/R can significantly activate the cardiomyocyte pyroptosis pathway, as evidenced by the activation of NLRP3 inflammasome and the upregulation of pyroptosis-related molecules such as Cleaved-Caspase-1, mature IL-1β, and mature IL-18. LR12 intervention can effectively reverse this effect, suggesting that TREM1 may be involved in the pathological regulation of MIRI by regulating the pyroptosis pathway. The NF-κB signaling pathway is the core pathway regulating inflammatory responses and cell death, and its abnormal activation can promote inflammatory cascade reactions and cell pyroptosis by regulating the expression of downstream target genes [14,15]. Through Western blotting and confocal detection of NF-κB nuclear translocation, this study confirmed that H/R/I/R can significantly activate the NF-κB signaling pathway, manifested by increased ratios of p-IκBα/IκBα and p-NF-κB/NF-κB, and enhanced p65 nuclear translocation. LR12 intervention can significantly inhibit the excessive activation of NF-κB signaling. Further experiments with PDTC, a specific NF-κB inhibitor, confirmed that inhibiting NF-κB can mimic the anti-pyroptotic effect of LR12, significantly reducing the expression of H/R-induced pyroptosis-related molecules, clarifying that NF-κB is a key downstream pathway of TREM1 in regulating cardiomyocyte pyroptosis. As the core initiating molecule of the pyroptosis pathway, the activation of NLRP3 inflammasome is a key link in the occurrence of pyroptosis. Through NLRP3 immunofluorescence experiments and intervention experiments with MCC950, a specific inhibitor, this study confirmed that TREM1-NF-κB signaling can regulate the expression and activation of NLRP3, and inhibiting NLRP3 can significantly reduce H/R-induced cardiomyocyte pyroptosis, which is consistent with the effect of LR12 intervention. These results indicate that TREM1 may promote the activation of NLRP3 inflammasome by activating the NF-κB signaling pathway, thereby initiating the cardiomyocyte pyroptosis program, forming a regulatory axis of “TREM1-NF-κB-NLRP3-pyroptosis” that participates in the pathological damage process of MIRI. This mechanism is consistent with the previous research that TREM1 regulates inflammatory responses through the NF-κB pathway [14,23,28], further enriching the mechanism of TREM1 in non-infectious inflammatory diseases and providing a new perspective for understanding the complex pathological mechanism of MIRI.

### 4.1. Limitations

Although this study systematically clarified the role and mechanism of TREM1 in MIRI, there are still certain limitations: First, this study mainly relied on pharmacological inhibitors (LR12, PDTC, MCC950) for mechanism verification, lacking genetic evidence from TREM1 knockout or knockdown models, making it difficult to completely rule out drug off-target effects. Subsequent experiments with TREM1 siRNA or knockout mice are needed to further clarify the specific role of TREM1; second, the ligand of TREM1 and its upstream activation signals have not been clarified, and its interaction with other inflammatory molecules needs further exploration; third, this study did not detect the activation of other parallel inflammatory pathways such as MAPKs and STATs, so it is impossible to exclude the potential role of these pathways in TREM1-regulated MIRI; fourth, the secretion levels of inflammatory factors such as IL-1β and IL-18 in cell supernatants and serum were not detected by ELISA, nor were pyroptosis positive controls such as nigericin/ATP set up, so the completeness of the experimental results needs further supplement.

## 5. Conclusions

Through in vitro and in vivo experiments, this study clearly confirmed that TREM1 expression is significantly upregulated after myocardial ischemia-reperfusion injury (MIRI), and its expression level is positively correlated with the degree of cardiomyocyte pyroptosis. Mechanistically, TREM1 can promote the activation of NLRP3 inflammasome by activating the NF-κB signaling pathway, thereby inducing the expression and release of pyroptosis-related molecules (GSDMD-N, Cleaved-Caspase-1, mature IL-1β, mature IL-18) and aggravating myocardial ischemia-reperfusion injury. Targeted blocking of TREM1 with LR12, a specific TREM1 inhibitor, can downregulate NLRP3 inflammasome activity by inhibiting the activation and nuclear translocation of the NF-κB signaling pathway, reduce cardiomyocyte pyroptosis, and at the same time alleviate myocardial tissue inflammatory infiltration and long-term fibrosis, improving short-term cardiac systolic function and long-term myocardial remodeling prognosis in MIRI mice. In addition, MCC950, a specific NLRP3 inhibitor, can mimic the anti-pyroptotic effect of LR12, further confirming that NLRP3 is a key downstream molecule of the TREM1-NF-κB pathway in regulating cardiomyocyte pyroptosis. In summary, this study is the first to systematically clarify the core regulatory role of the TREM1-NF-κB-NLRP3 signaling axis in MIRI-induced cardiomyocyte pyroptosis, confirming that TREM1 can serve as a potential therapeutic target for myocardial ischemia-reperfusion injury. It provides a solid experimental basis and theoretical foundation for the development of targeted immunotherapeutic strategies, which is expected to enrich the clinical treatment methods of MIRI and improve patient prognosis.

## Supporting information

S1 File Raw data of fluorescence staining. This zip file (fluorescence.zip) contains the original files of fluorescence imaging experiments.
(ZIP)

S2 File Experimental data of C57 mice. This zip file (C57.zip) includes the raw experimental materials related to the C57 mouse model.
(ZIP)

S3 File Raw data of CCK8 and LDH assays. This zip file (CCK8 和 LDH.zip) contains the original data files of cell viability (CCK8) and lactate dehydrogenase (LDH) detection.
(ZIP)

S4 File Raw Western blot images for Figure 1. This PDF file (S4_Figure1_WB.pdf) contains the uncropped and minimally adjusted original blot images corresponding to Figure 1, with clear labels of sample groups and molecular weight markers. (PDF)

S5 File Raw Western blot images for Figure 2. This PDF file (S5_Figure2_WB.pdf) contains the uncropped and minimally adjusted original blot images corresponding to Figure 2, with clear labels of sample groups and molecular weight markers. (PDF)

S6 File Raw Western blot images for Figure 3. This PDF file (S6_Figure3_WB.pdf) contains the uncropped and minimally adjusted original blot images corresponding to Figure 3, with clear labels of sample groups and molecular weight markers. (PDF)

S7 File Raw Western blot images for Figure 4. This PDF file (S7_Figure4_WB.pdf) contains the uncropped and minimally adjusted original blot images corresponding to Figure 4, with clear labels of sample groups and molecular weight markers. (PDF)

S8 File Raw Western blot images for Figure 5. This PDF file (S8_Figure5_WB.pdf) contains the uncropped and minimally adjusted original blot images corresponding to Figure 5, with clear labels of sample groups and molecular weight markers. (PDF)

## Abbreviation

TREM-1: Triggering Receptor Expressed on Myeloid Cells-1
NF-κB: Nuclear Factor-κB
NLRP3: NOD-like Receptor Pyrin Domain-Containing Protein 3
H/R/HR: Hypoxia/Reoxygenation
MIRI: Myocardial Ischemia-Reperfusion Injury
I/R/IR: Ischemia-Reperfusion Injury
