# Supplementary figures and images for: Inhibition of TREM1 attenuates myocardial ischemia-reperfusion injury-induced cardiomyocyte pyroptosis by suppressing the activation of the NF-κB signaling pathway
Source: PLoS One. 2026 Jan 9;21(1):e0340382. doi: 10.1371/journal.pone.0340382 (PMC12788684; doi:10.1371/journal.pone.0340382)

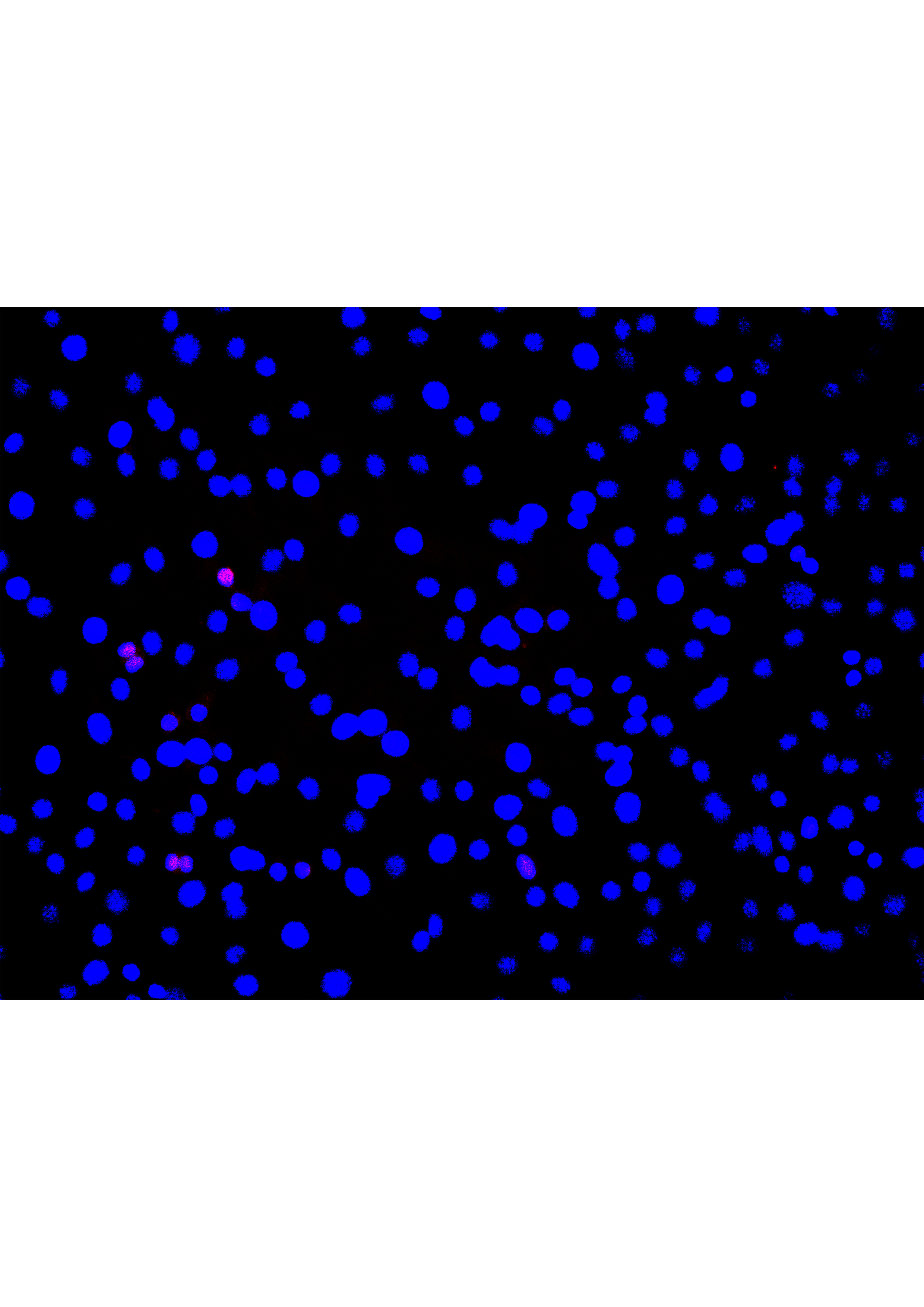

Supplement: S1 File — This zip file (fluorescence.zip) contains the original files of fluorescence imaging experiments. (ZIP) [file pone.0340382.s001.zip › fluorescence/Hoechst-PI/HR+LR12/HR+LR12 MG.png]

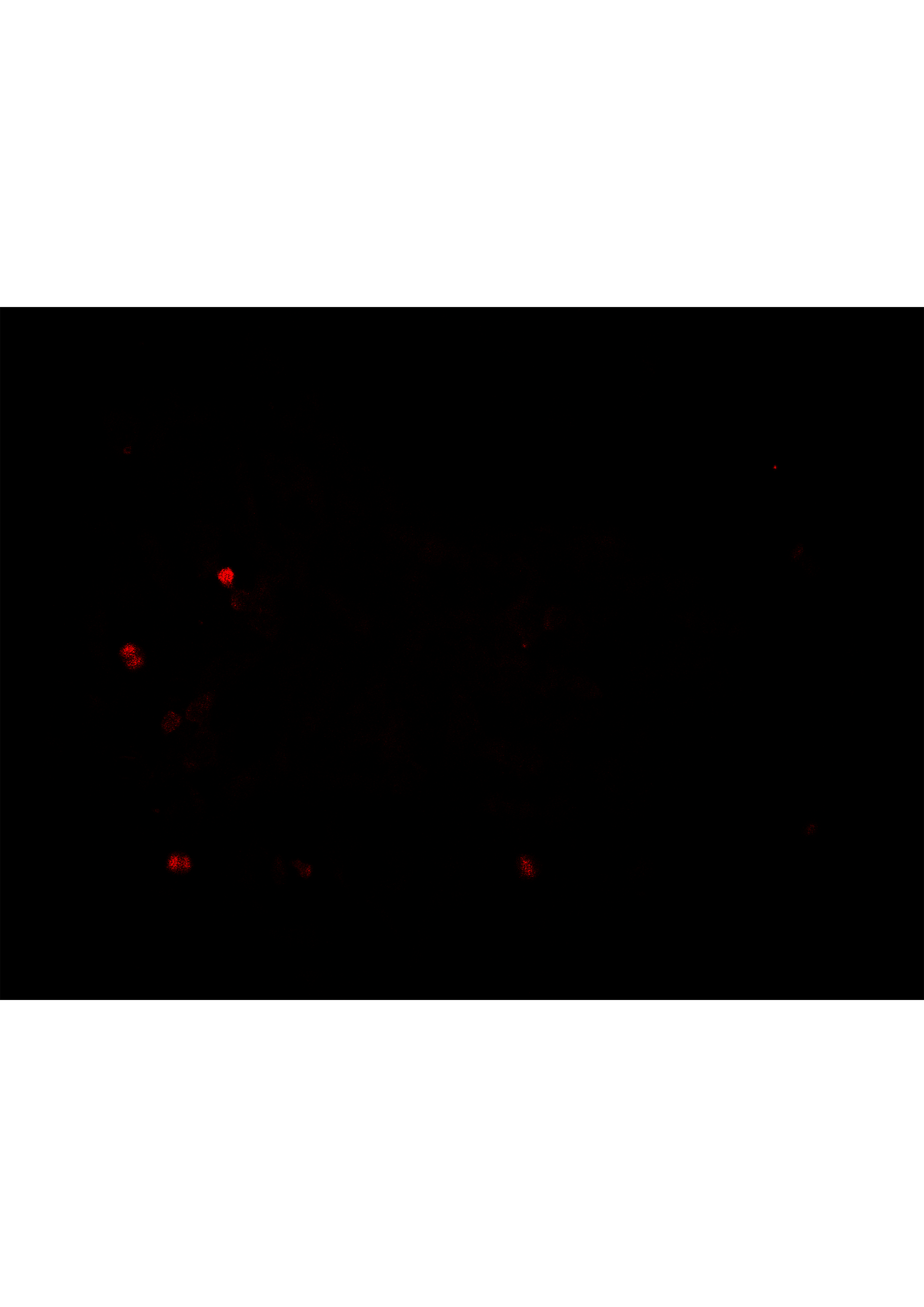

Supplement: S1 File — This zip file (fluorescence.zip) contains the original files of fluorescence imaging experiments. (ZIP) [file pone.0340382.s001.zip › fluorescence/Hoechst-PI/HR+LR12/HR+LR12 PI.png]

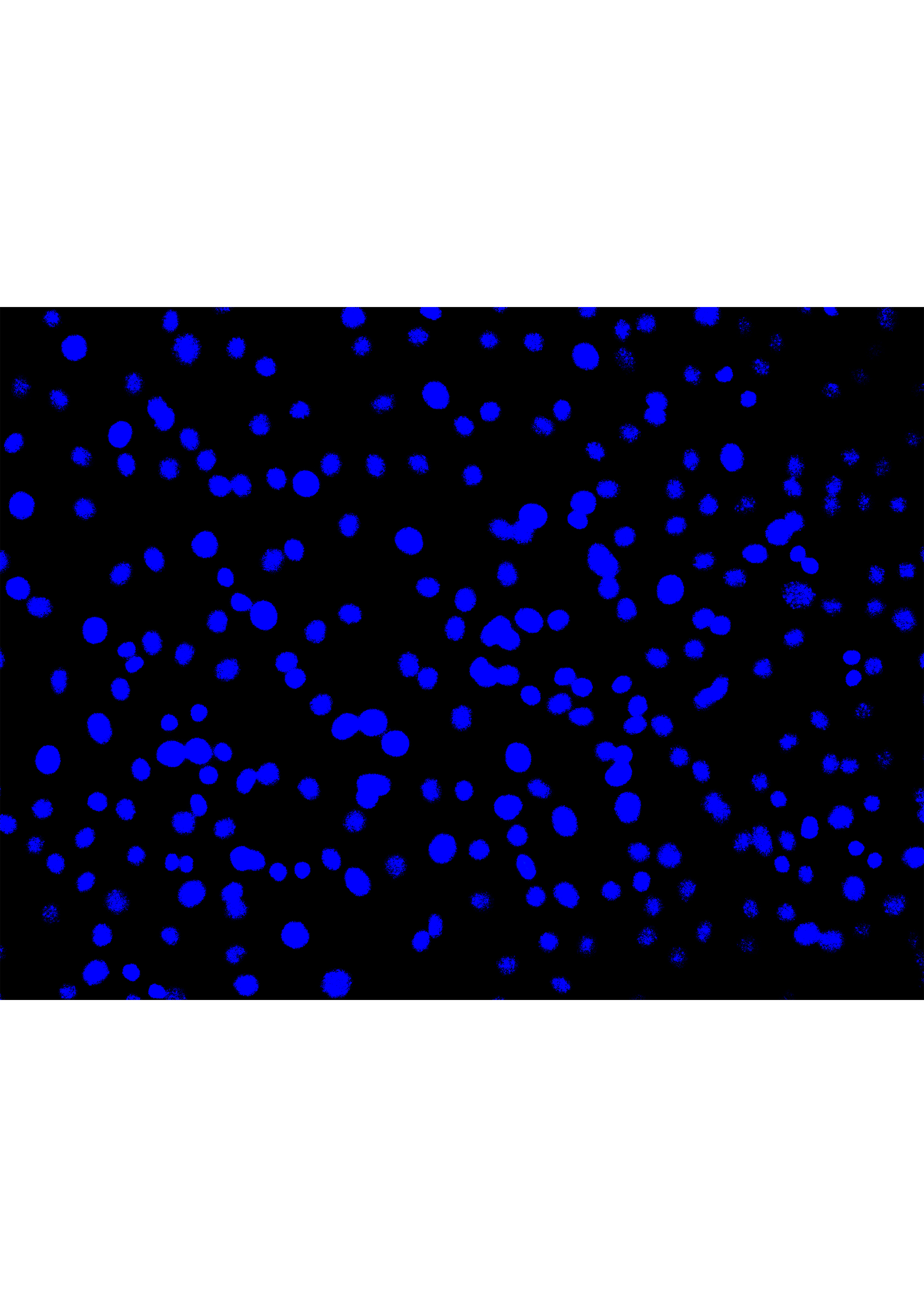

Supplement: S1 File — This zip file (fluorescence.zip) contains the original files of fluorescence imaging experiments. (ZIP) [file pone.0340382.s001.zip › fluorescence/Hoechst-PI/HR+LR12/HR+LR12.png]

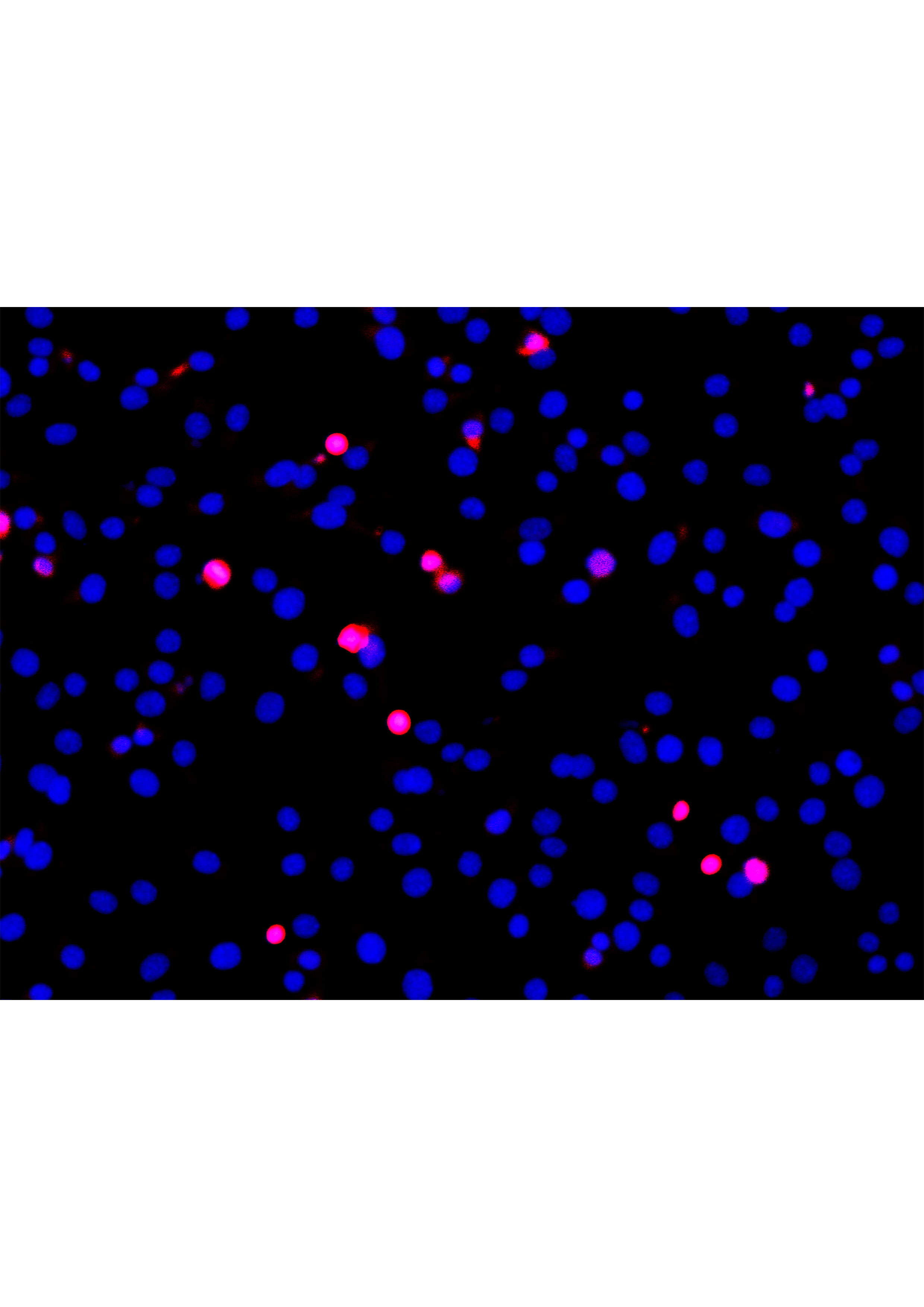

Supplement: S1 File — This zip file (fluorescence.zip) contains the original files of fluorescence imaging experiments. (ZIP) [file pone.0340382.s001.zip › fluorescence/Hoechst-PI/HR/HR MG.png]

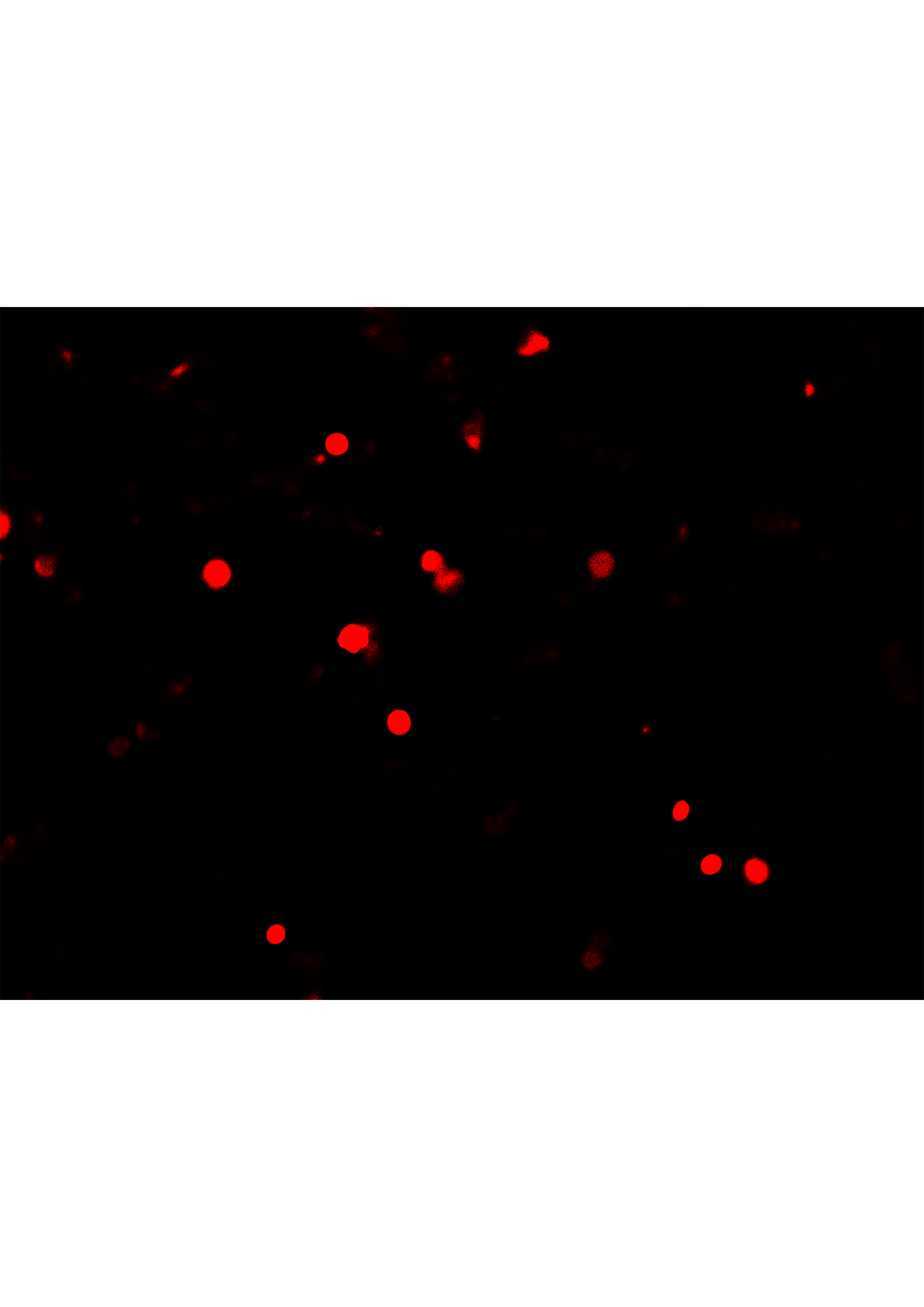

Supplement: S1 File — This zip file (fluorescence.zip) contains the original files of fluorescence imaging experiments. (ZIP) [file pone.0340382.s001.zip › fluorescence/Hoechst-PI/HR/HR PI.png]

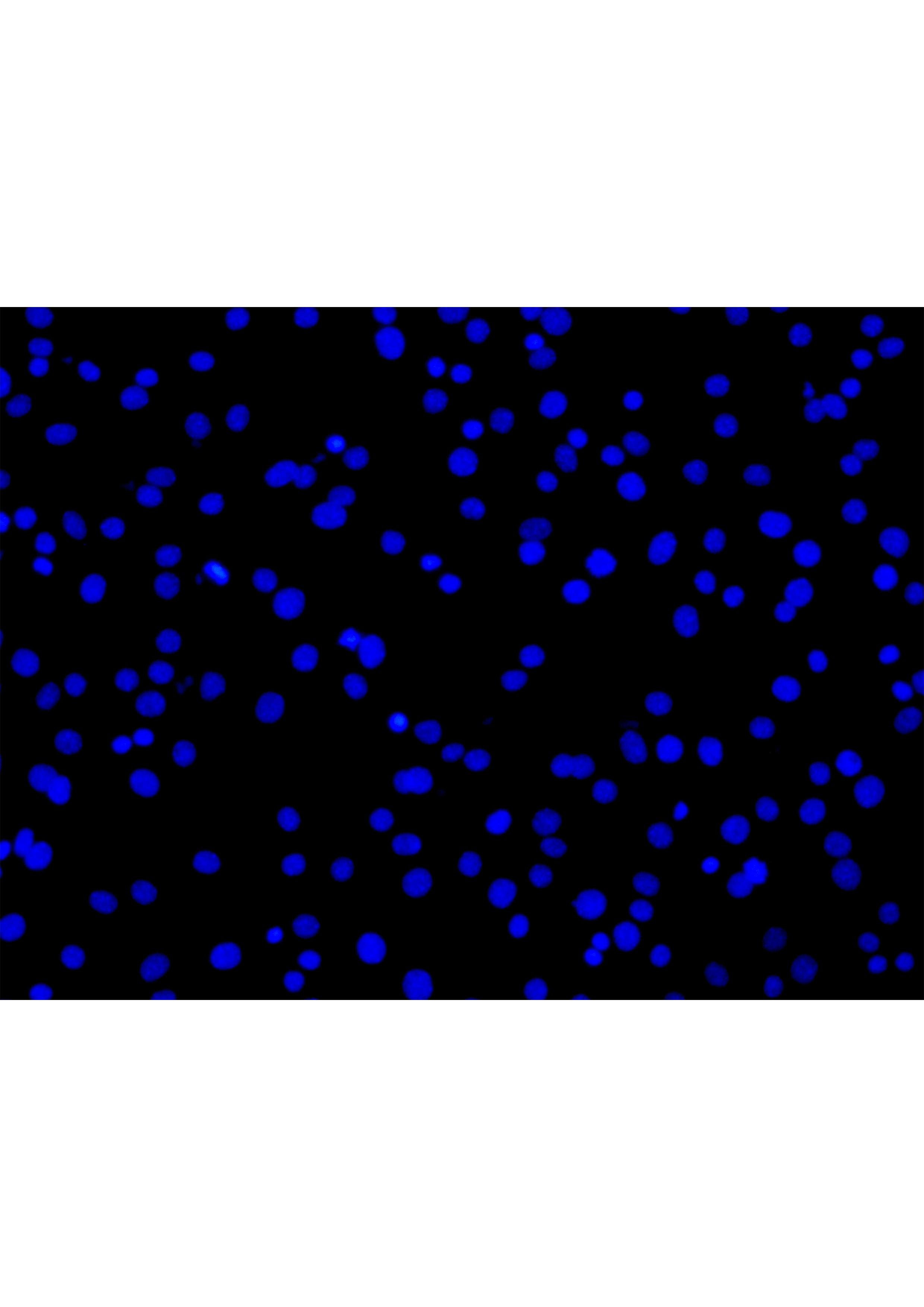

Supplement: S1 File — This zip file (fluorescence.zip) contains the original files of fluorescence imaging experiments. (ZIP) [file pone.0340382.s001.zip › fluorescence/Hoechst-PI/HR/HR.png]

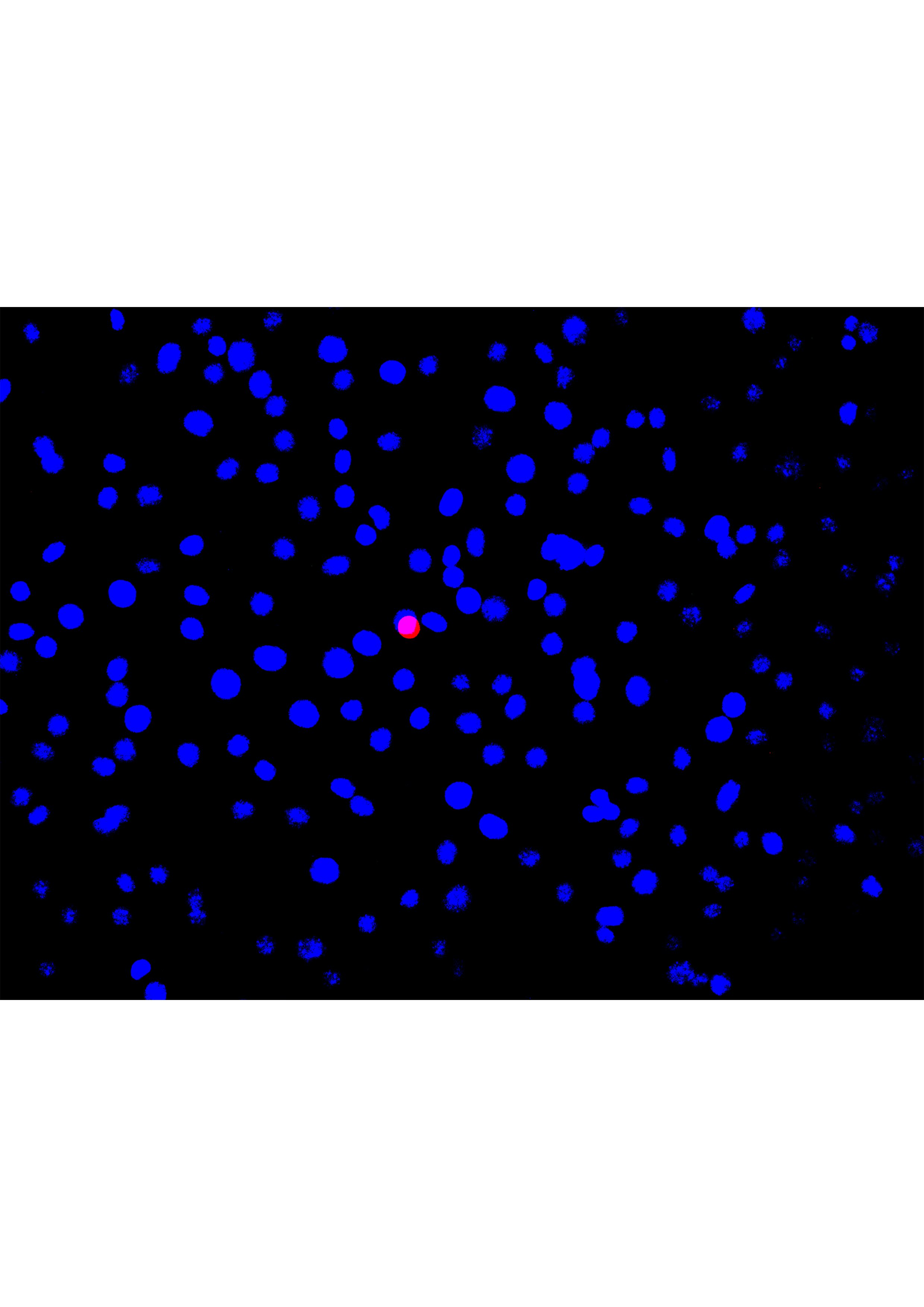

Supplement: S1 File — This zip file (fluorescence.zip) contains the original files of fluorescence imaging experiments. (ZIP) [file pone.0340382.s001.zip › fluorescence/Hoechst-PI/NC+LR12/NC+LR12 MG.png]

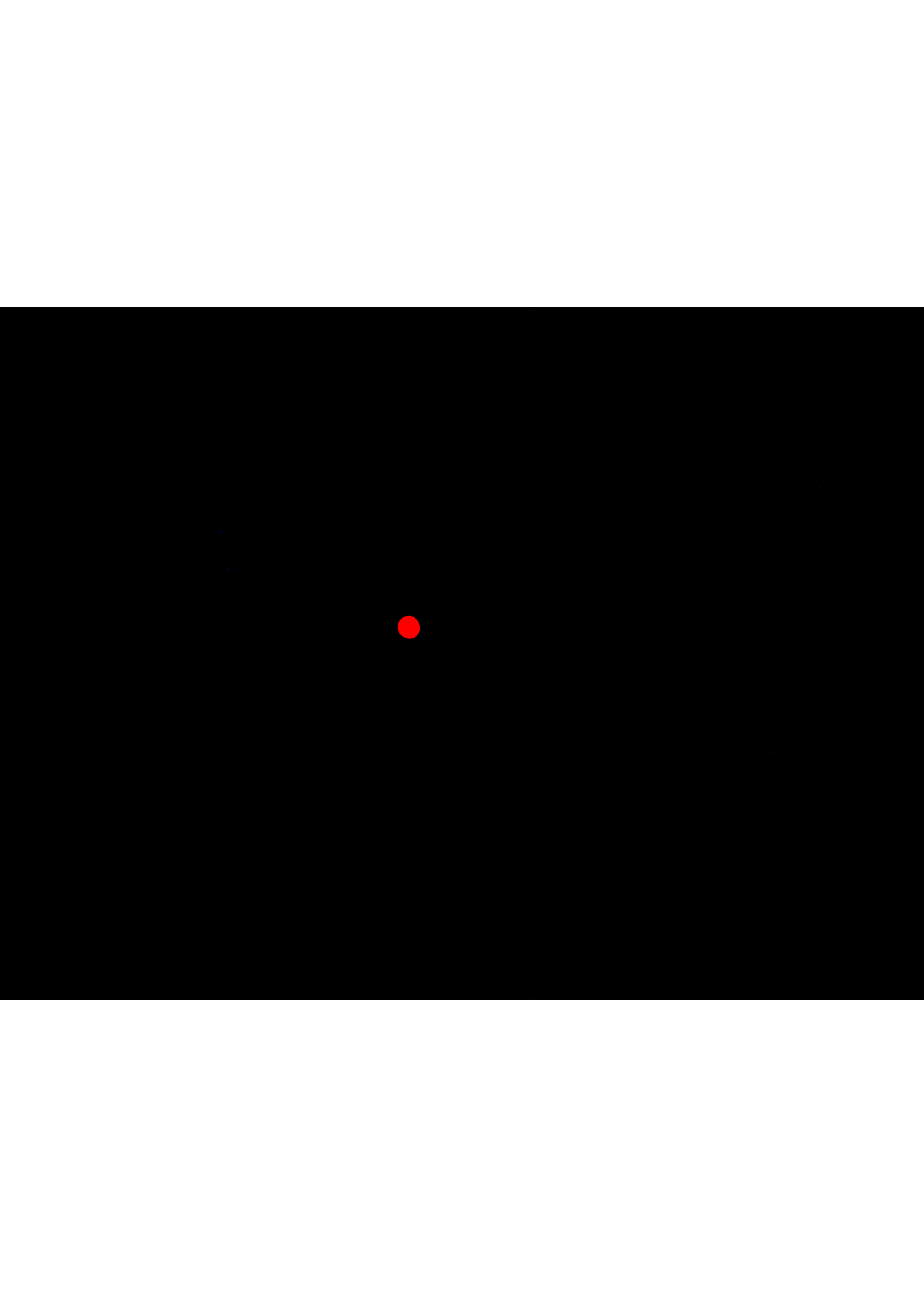

Supplement: S1 File — This zip file (fluorescence.zip) contains the original files of fluorescence imaging experiments. (ZIP) [file pone.0340382.s001.zip › fluorescence/Hoechst-PI/NC+LR12/NC+LR12 PI.png]

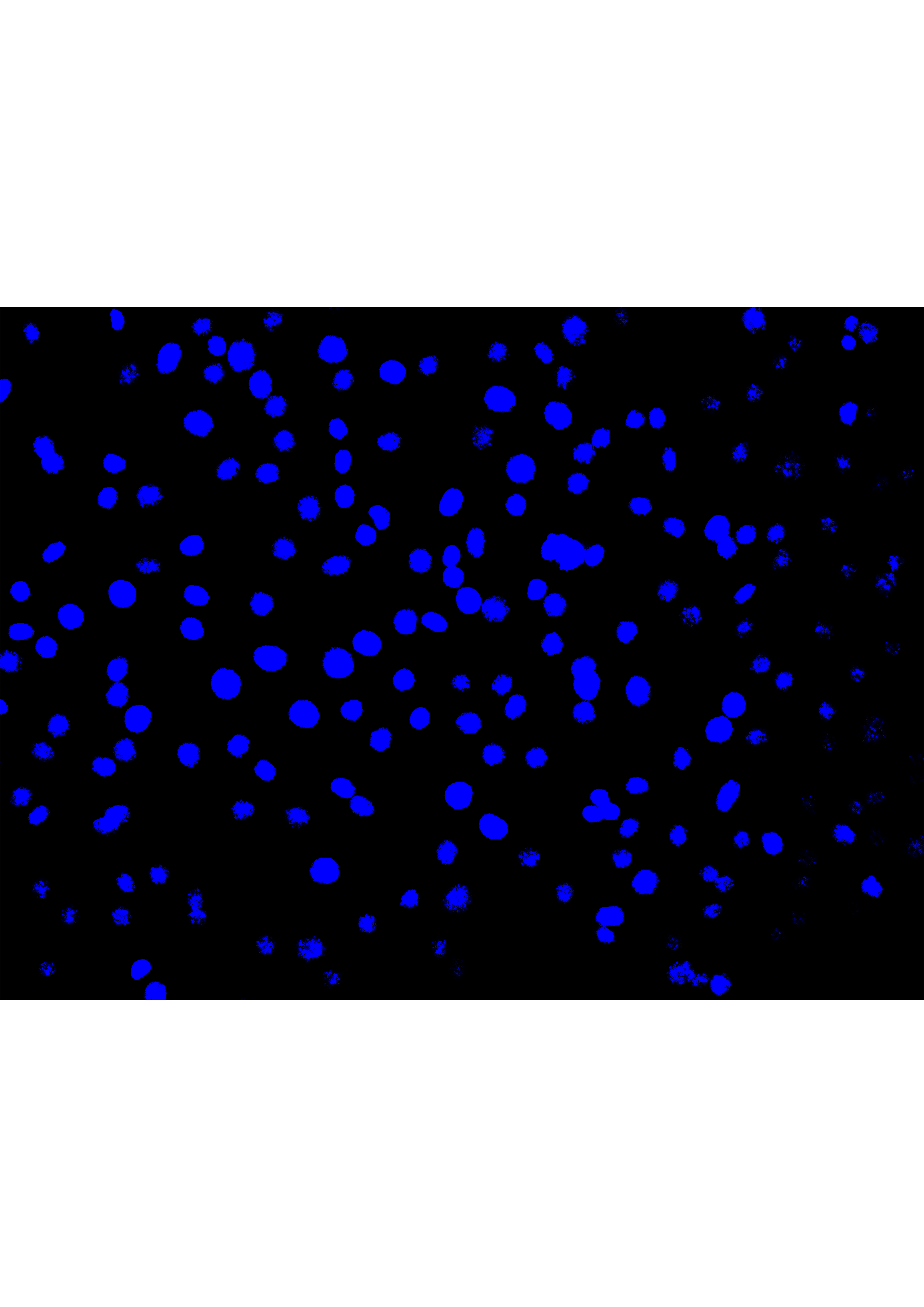

Supplement: S1 File — This zip file (fluorescence.zip) contains the original files of fluorescence imaging experiments. (ZIP) [file pone.0340382.s001.zip › fluorescence/Hoechst-PI/NC+LR12/NC+LR12.png]

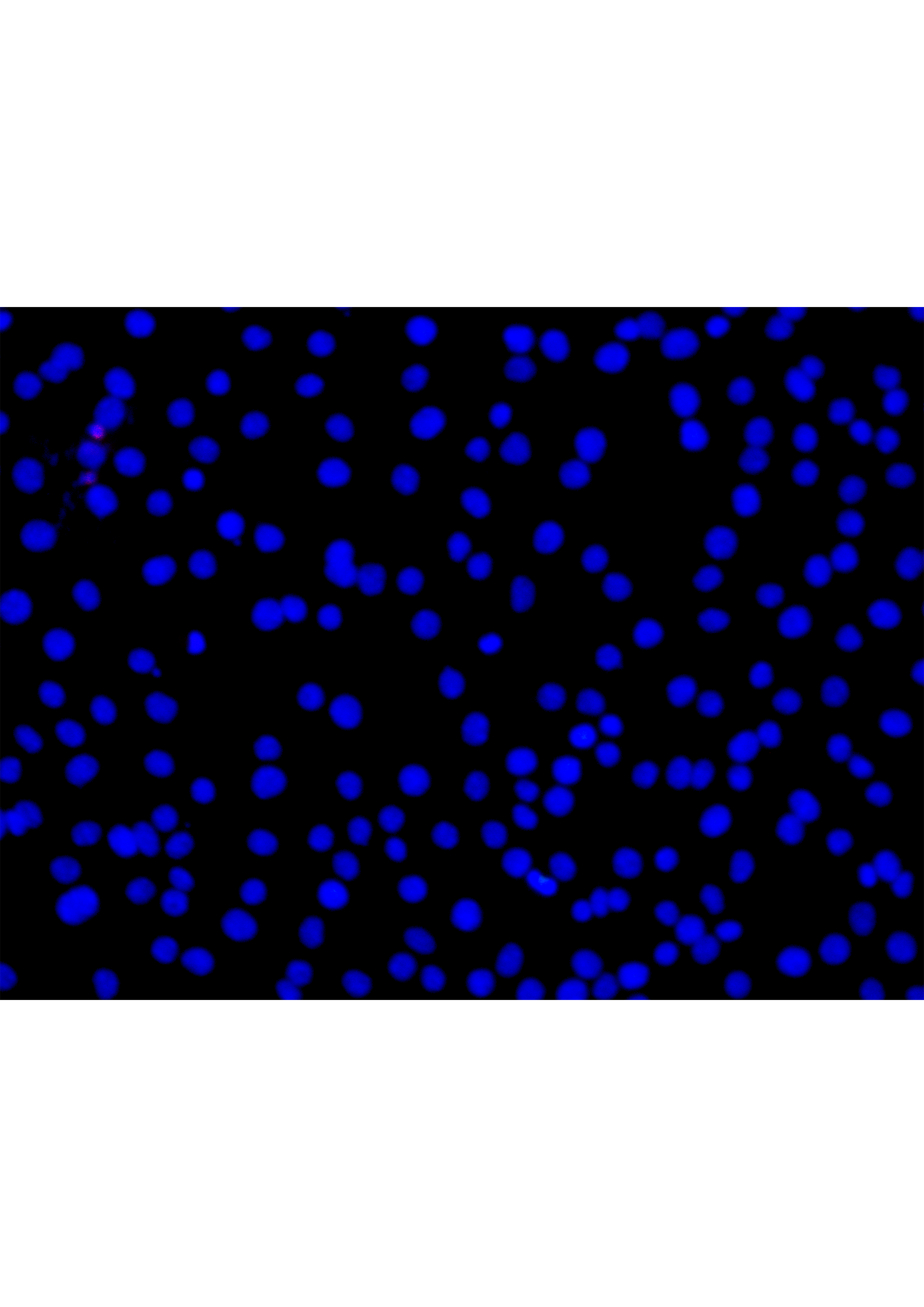

Supplement: S1 File — This zip file (fluorescence.zip) contains the original files of fluorescence imaging experiments. (ZIP) [file pone.0340382.s001.zip › fluorescence/Hoechst-PI/NC/NC MG.png]

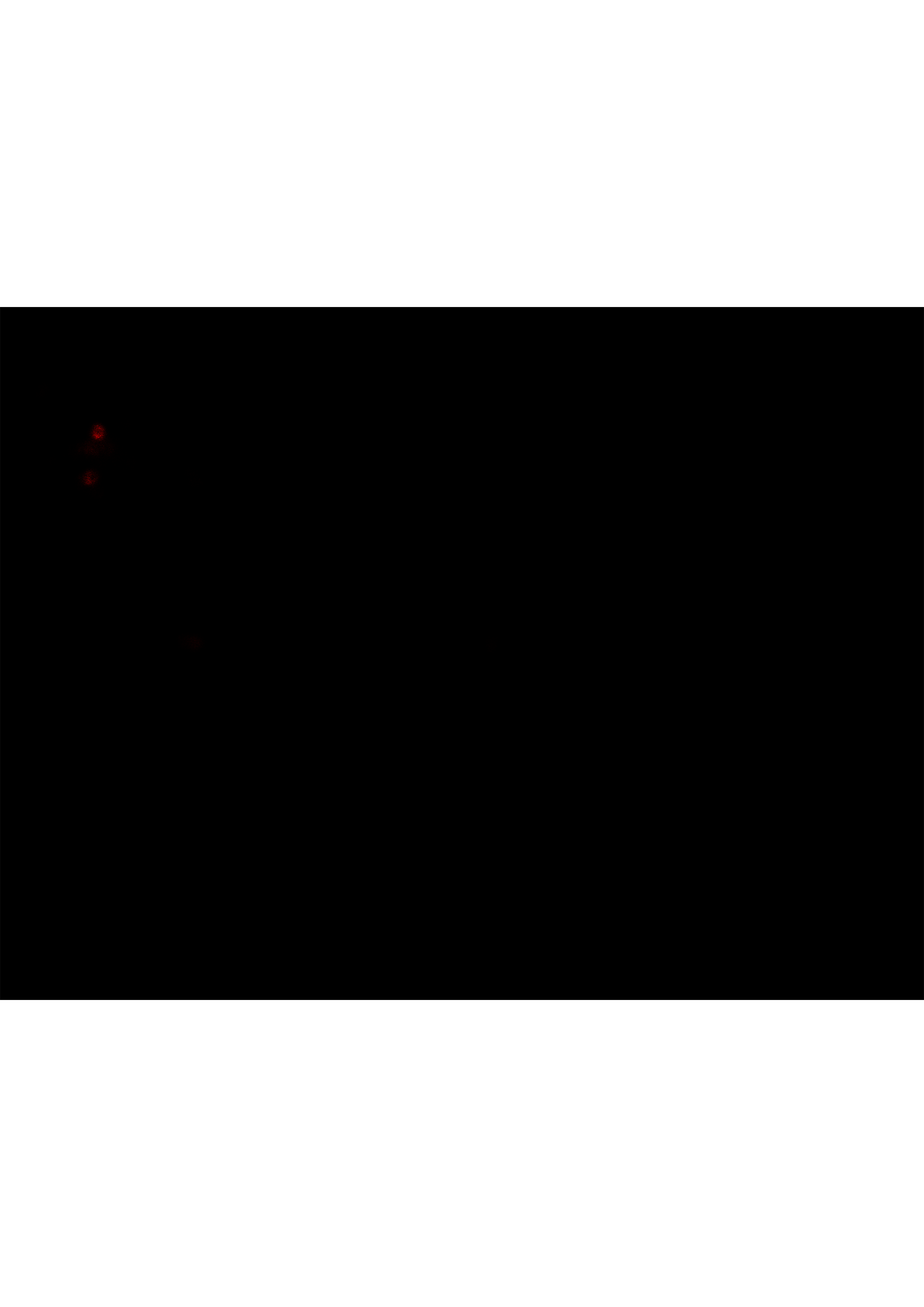

Supplement: S1 File — This zip file (fluorescence.zip) contains the original files of fluorescence imaging experiments. (ZIP) [file pone.0340382.s001.zip › fluorescence/Hoechst-PI/NC/NC PI.png]

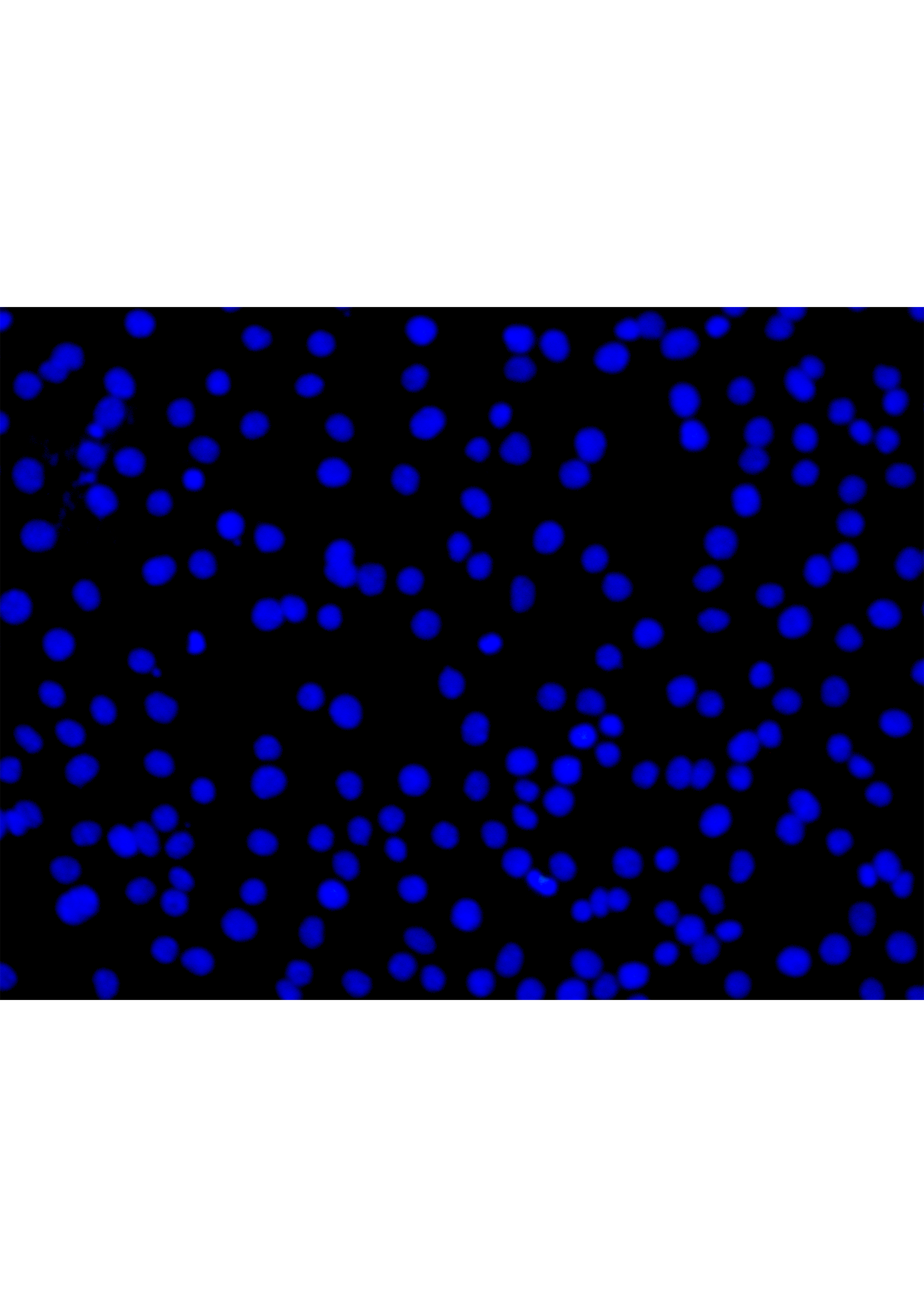

Supplement: S1 File — This zip file (fluorescence.zip) contains the original files of fluorescence imaging experiments. (ZIP) [file pone.0340382.s001.zip › fluorescence/Hoechst-PI/NC/NC.png]

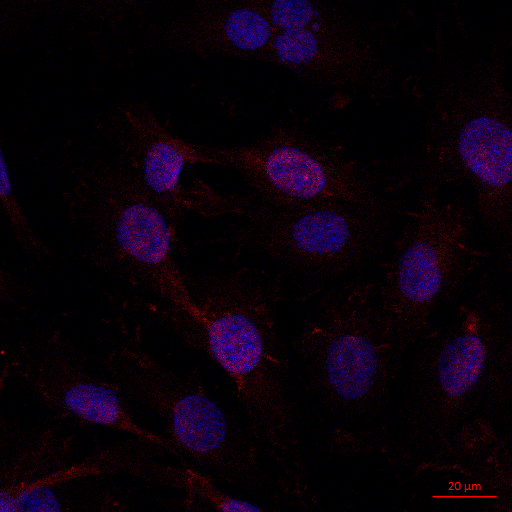

Supplement: S1 File — This zip file (fluorescence.zip) contains the original files of fluorescence imaging experiments. (ZIP) [file pone.0340382.s001.zip › fluorescence/NF-KB confocal/HR+LR12/Snap-426/Snap-426_c1+2.tif]

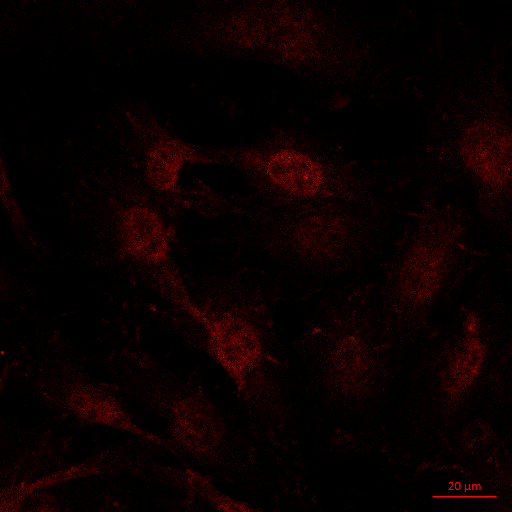

Supplement: S1 File — This zip file (fluorescence.zip) contains the original files of fluorescence imaging experiments. (ZIP) [file pone.0340382.s001.zip › fluorescence/NF-KB confocal/HR+LR12/Snap-426/Snap-426_c1.tif]

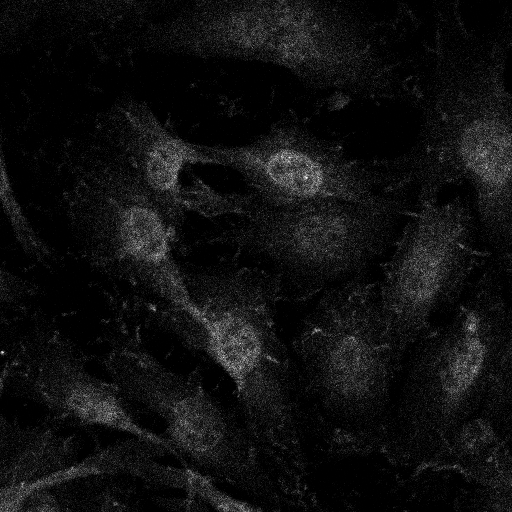

Supplement: S1 File — This zip file (fluorescence.zip) contains the original files of fluorescence imaging experiments. (ZIP) [file pone.0340382.s001.zip › fluorescence/NF-KB confocal/HR+LR12/Snap-426/Snap-426_c1_ORG.tif]

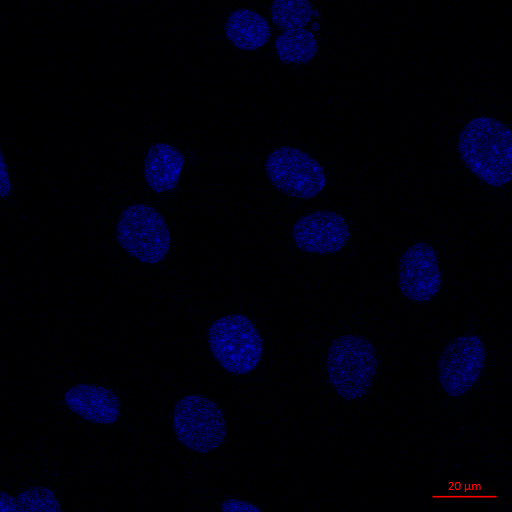

Supplement: S1 File — This zip file (fluorescence.zip) contains the original files of fluorescence imaging experiments. (ZIP) [file pone.0340382.s001.zip › fluorescence/NF-KB confocal/HR+LR12/Snap-426/Snap-426_c2.tif]

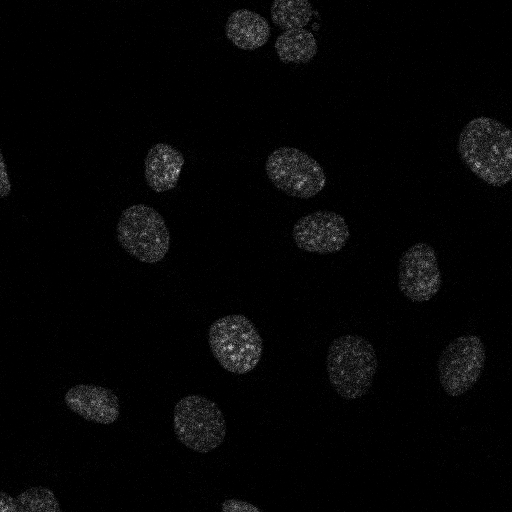

Supplement: S1 File — This zip file (fluorescence.zip) contains the original files of fluorescence imaging experiments. (ZIP) [file pone.0340382.s001.zip › fluorescence/NF-KB confocal/HR+LR12/Snap-426/Snap-426_c2_ORG.tif]

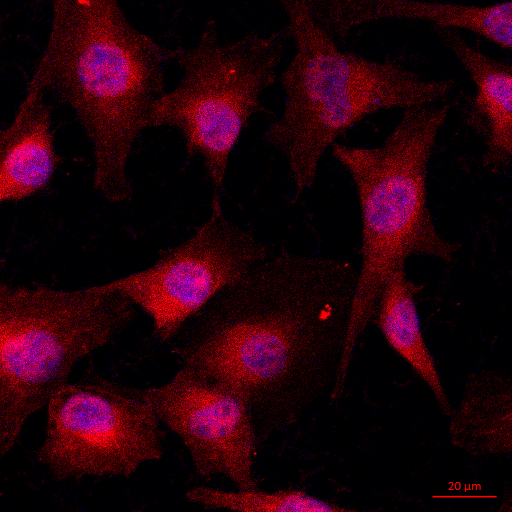

Supplement: S1 File — This zip file (fluorescence.zip) contains the original files of fluorescence imaging experiments. (ZIP) [file pone.0340382.s001.zip › fluorescence/NF-KB confocal/HR/Snap-428/Snap-428_c1+2.tif]

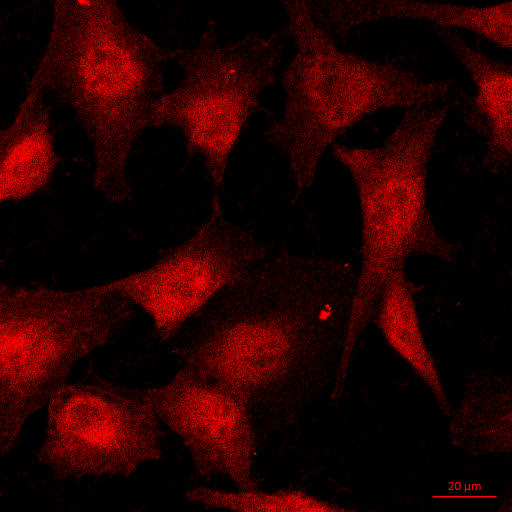

Supplement: S1 File — This zip file (fluorescence.zip) contains the original files of fluorescence imaging experiments. (ZIP) [file pone.0340382.s001.zip › fluorescence/NF-KB confocal/HR/Snap-428/Snap-428_c1.tif]

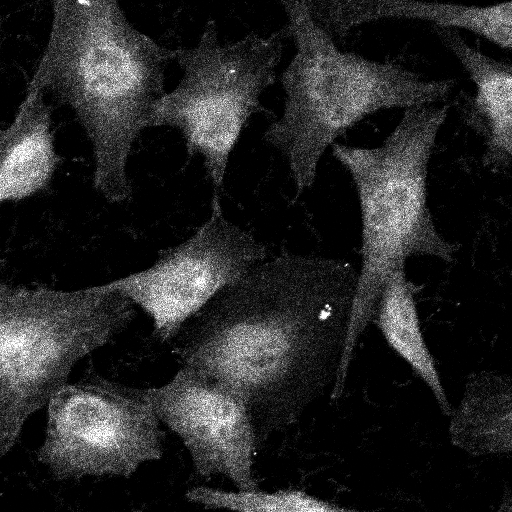

Supplement: S1 File — This zip file (fluorescence.zip) contains the original files of fluorescence imaging experiments. (ZIP) [file pone.0340382.s001.zip › fluorescence/NF-KB confocal/HR/Snap-428/Snap-428_c1_ORG.tif]

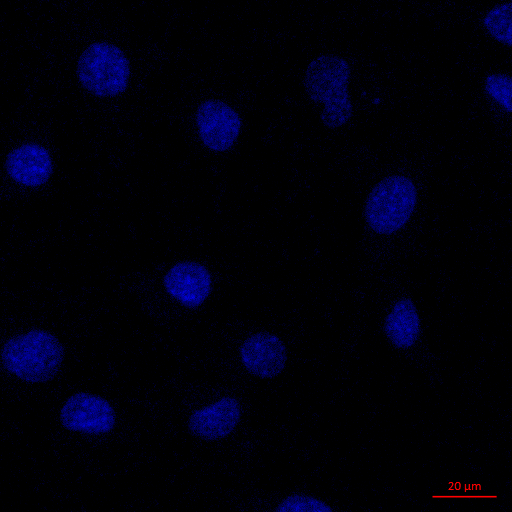

Supplement: S1 File — This zip file (fluorescence.zip) contains the original files of fluorescence imaging experiments. (ZIP) [file pone.0340382.s001.zip › fluorescence/NF-KB confocal/HR/Snap-428/Snap-428_c2.tif]

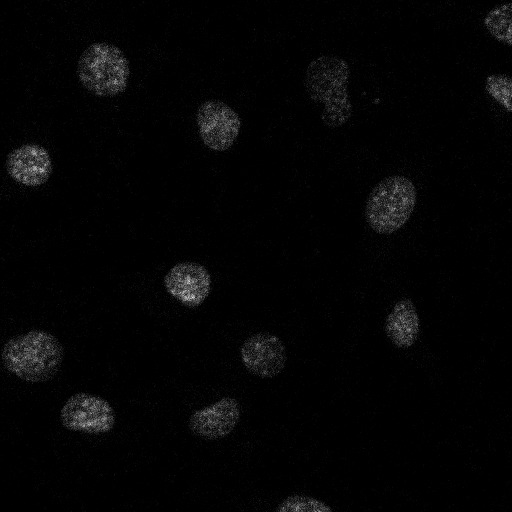

Supplement: S1 File — This zip file (fluorescence.zip) contains the original files of fluorescence imaging experiments. (ZIP) [file pone.0340382.s001.zip › fluorescence/NF-KB confocal/HR/Snap-428/Snap-428_c2_ORG.tif]

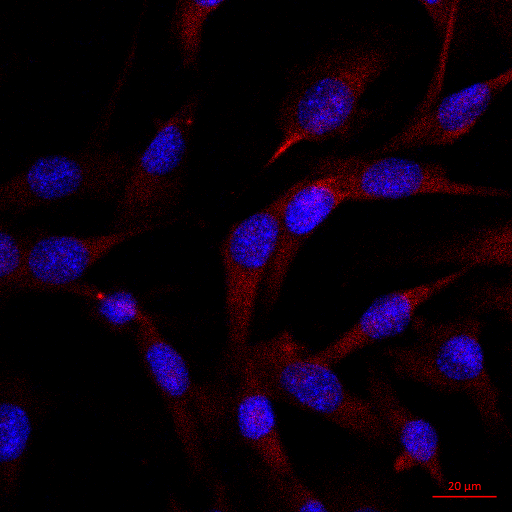

Supplement: S1 File — This zip file (fluorescence.zip) contains the original files of fluorescence imaging experiments. (ZIP) [file pone.0340382.s001.zip › fluorescence/NF-KB confocal/NC+LR12/Snap-411/Snap-411_c1+2.tif]

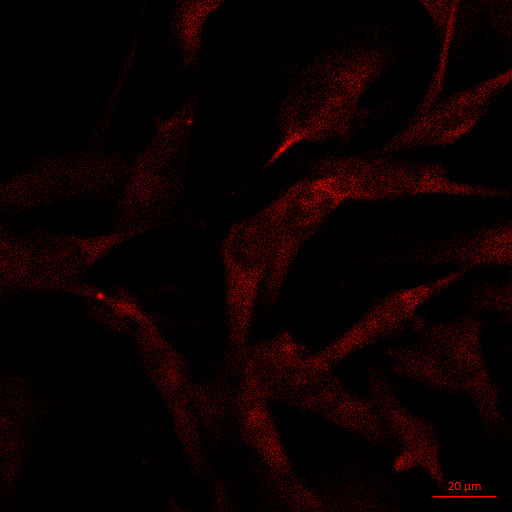

Supplement: S1 File — This zip file (fluorescence.zip) contains the original files of fluorescence imaging experiments. (ZIP) [file pone.0340382.s001.zip › fluorescence/NF-KB confocal/NC+LR12/Snap-411/Snap-411_c1.tif]

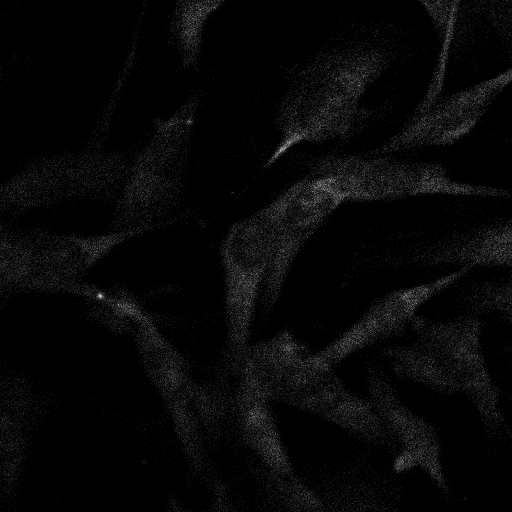

Supplement: S1 File — This zip file (fluorescence.zip) contains the original files of fluorescence imaging experiments. (ZIP) [file pone.0340382.s001.zip › fluorescence/NF-KB confocal/NC+LR12/Snap-411/Snap-411_c1_ORG.tif]

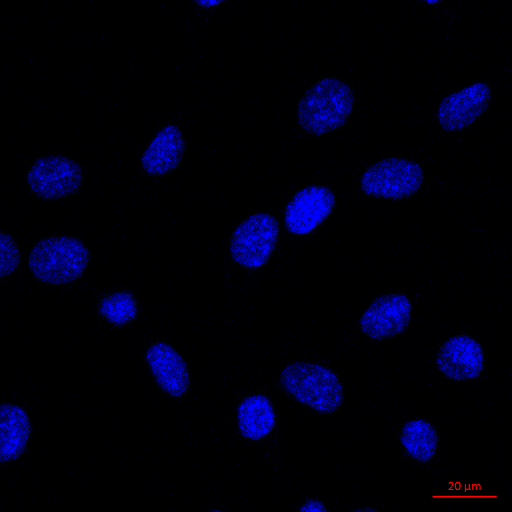

Supplement: S1 File — This zip file (fluorescence.zip) contains the original files of fluorescence imaging experiments. (ZIP) [file pone.0340382.s001.zip › fluorescence/NF-KB confocal/NC+LR12/Snap-411/Snap-411_c2.tif]

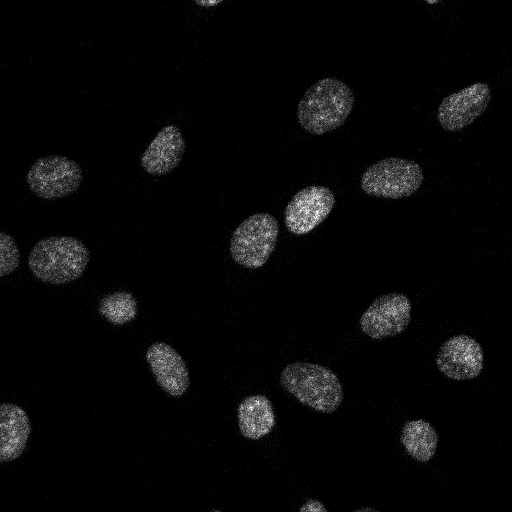

Supplement: S1 File — This zip file (fluorescence.zip) contains the original files of fluorescence imaging experiments. (ZIP) [file pone.0340382.s001.zip › fluorescence/NF-KB confocal/NC+LR12/Snap-411/Snap-411_c2_ORG.tif]

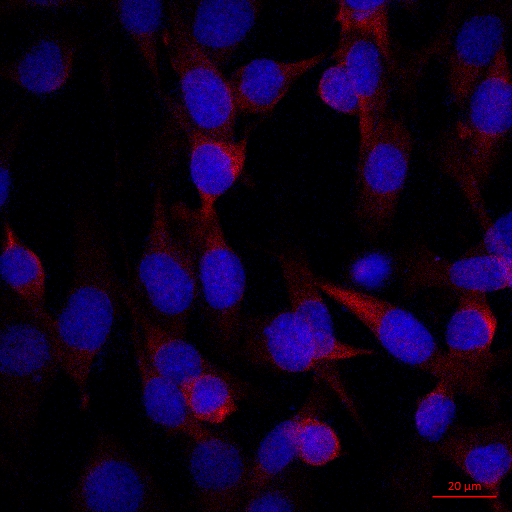

Supplement: S1 File — This zip file (fluorescence.zip) contains the original files of fluorescence imaging experiments. (ZIP) [file pone.0340382.s001.zip › fluorescence/NF-KB confocal/NC/Snap-417/Snap-417_c1+2.tif]

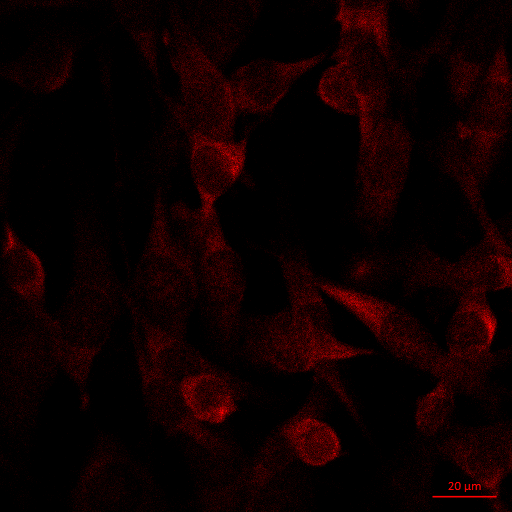

Supplement: S1 File — This zip file (fluorescence.zip) contains the original files of fluorescence imaging experiments. (ZIP) [file pone.0340382.s001.zip › fluorescence/NF-KB confocal/NC/Snap-417/Snap-417_c1.tif]

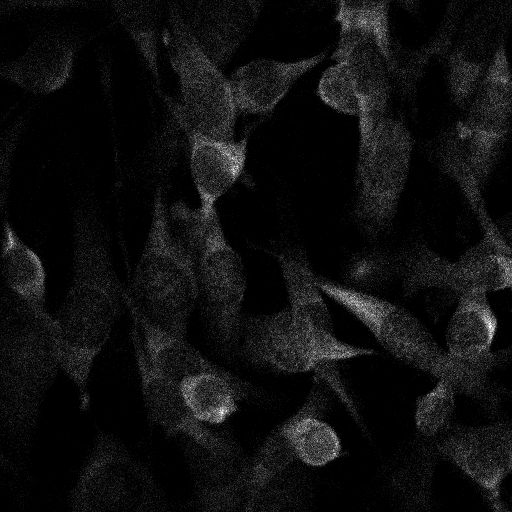

Supplement: S1 File — This zip file (fluorescence.zip) contains the original files of fluorescence imaging experiments. (ZIP) [file pone.0340382.s001.zip › fluorescence/NF-KB confocal/NC/Snap-417/Snap-417_c1_ORG.tif]

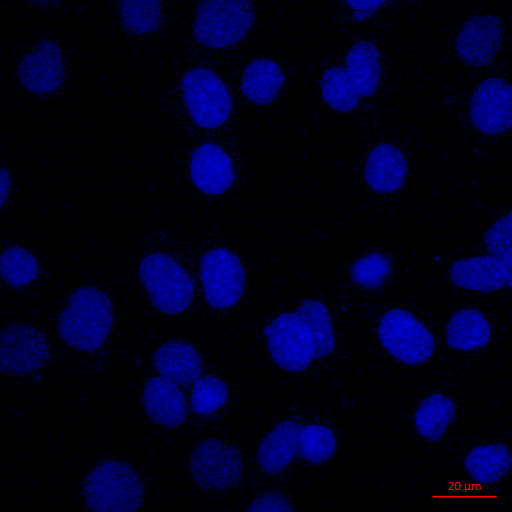

Supplement: S1 File — This zip file (fluorescence.zip) contains the original files of fluorescence imaging experiments. (ZIP) [file pone.0340382.s001.zip › fluorescence/NF-KB confocal/NC/Snap-417/Snap-417_c2.tif]

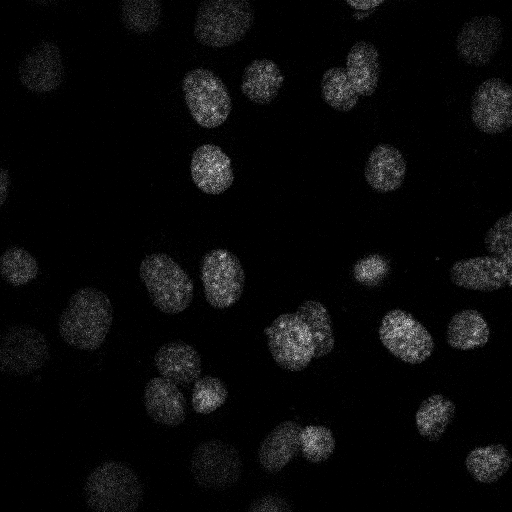

Supplement: S1 File — This zip file (fluorescence.zip) contains the original files of fluorescence imaging experiments. (ZIP) [file pone.0340382.s001.zip › fluorescence/NF-KB confocal/NC/Snap-417/Snap-417_c2_ORG.tif]

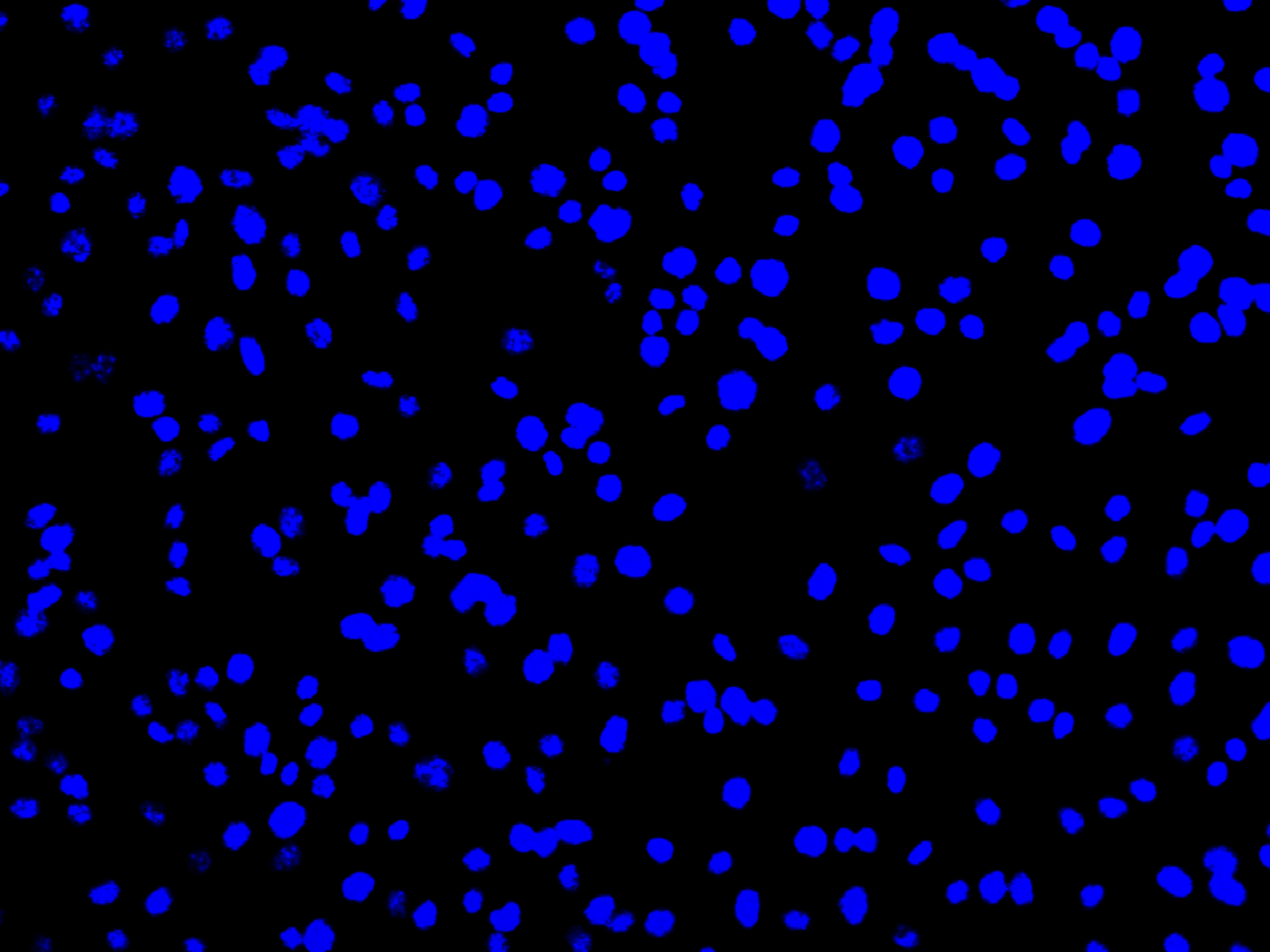

Supplement: S1 File — This zip file (fluorescence.zip) contains the original files of fluorescence imaging experiments. (ZIP) [file pone.0340382.s001.zip › fluorescence/NLPR3 fluorescence/NLRP3/HR+PDTC/1_image_A.tif]

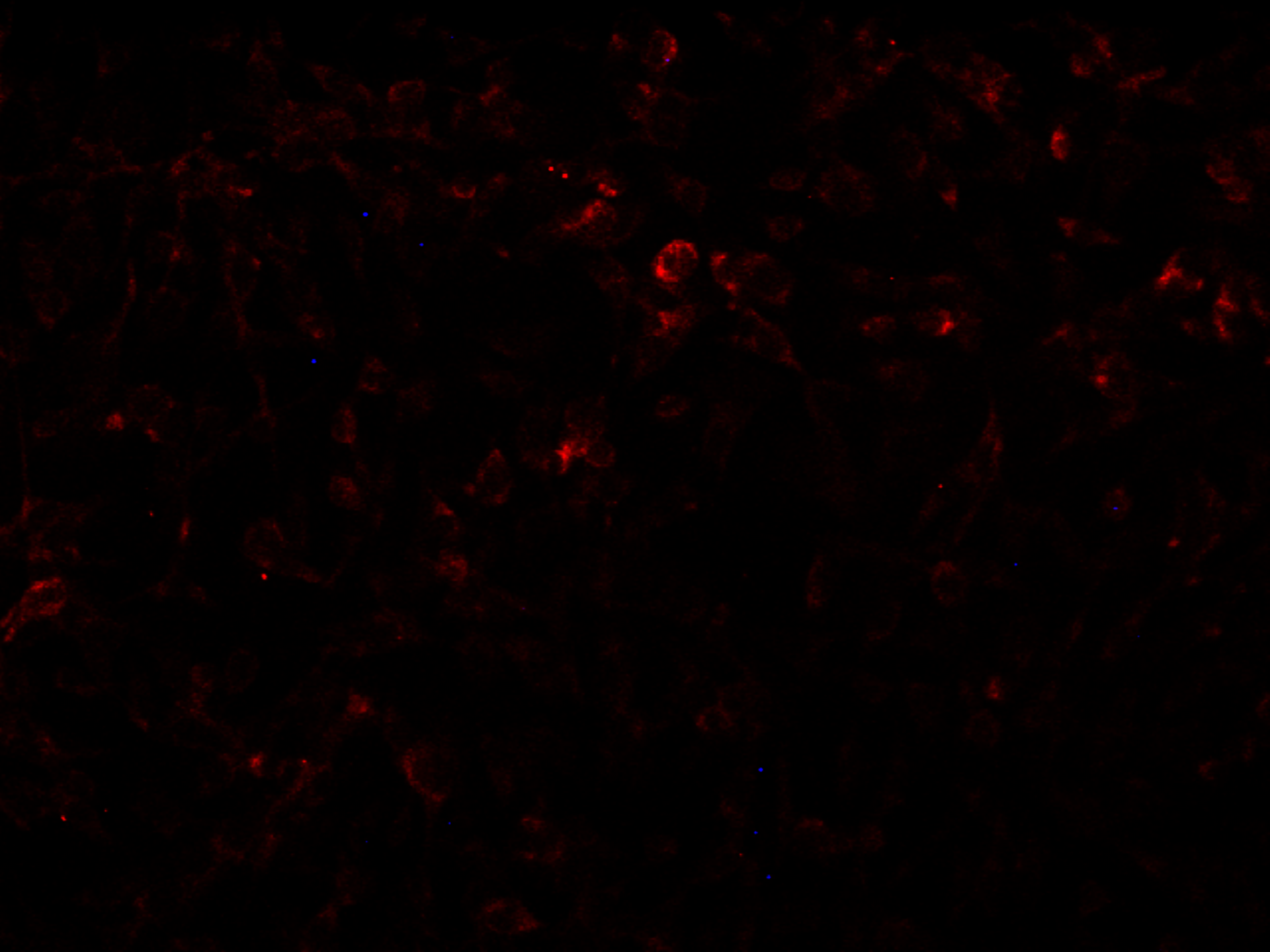

Supplement: S1 File — This zip file (fluorescence.zip) contains the original files of fluorescence imaging experiments. (ZIP) [file pone.0340382.s001.zip › fluorescence/NLPR3 fluorescence/NLRP3/HR+PDTC/3_image_N21.tif]

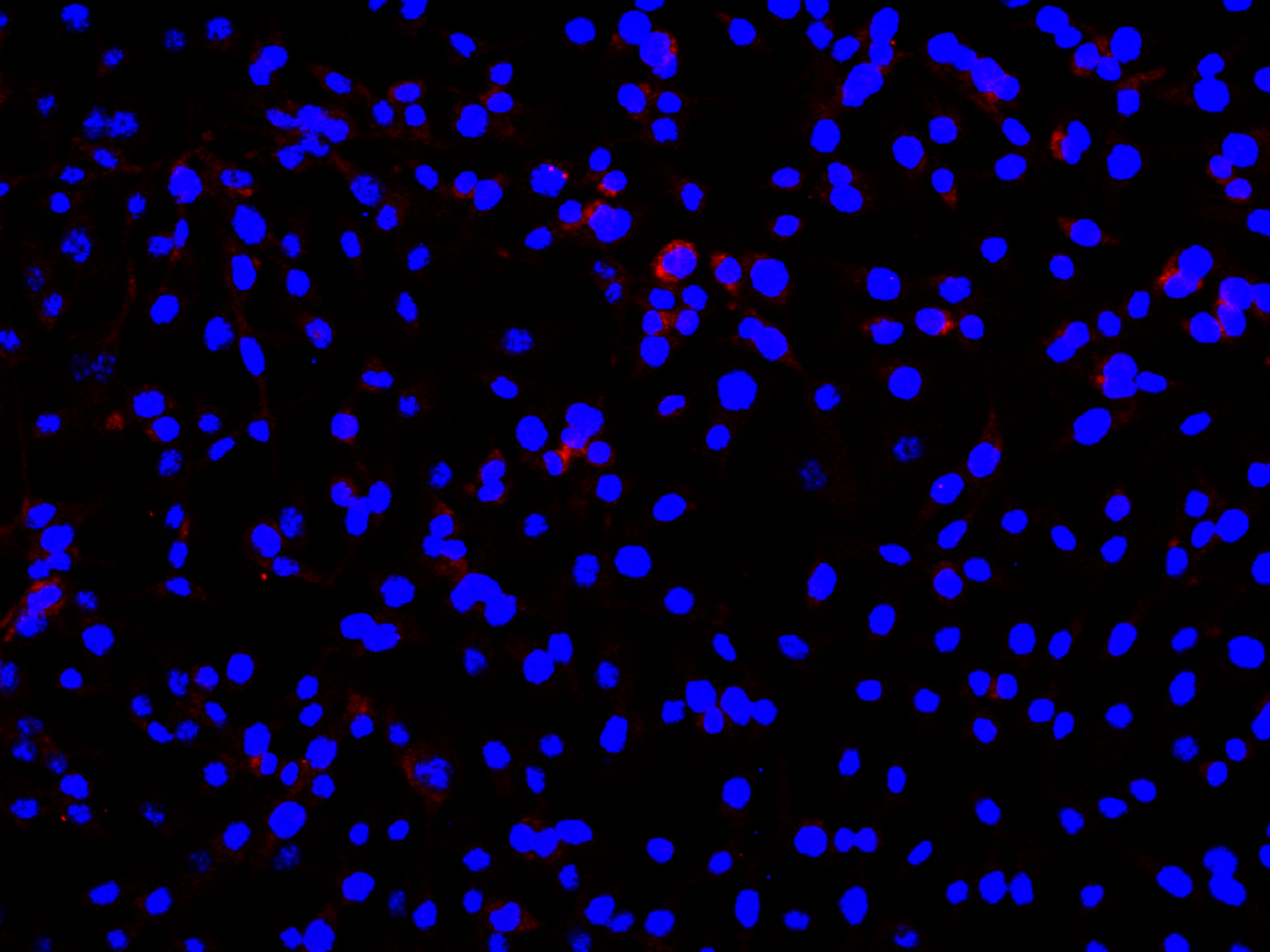

Supplement: S1 File — This zip file (fluorescence.zip) contains the original files of fluorescence imaging experiments. (ZIP) [file pone.0340382.s001.zip › fluorescence/NLPR3 fluorescence/NLRP3/HR+PDTC/Overlay_Maximum.tif]

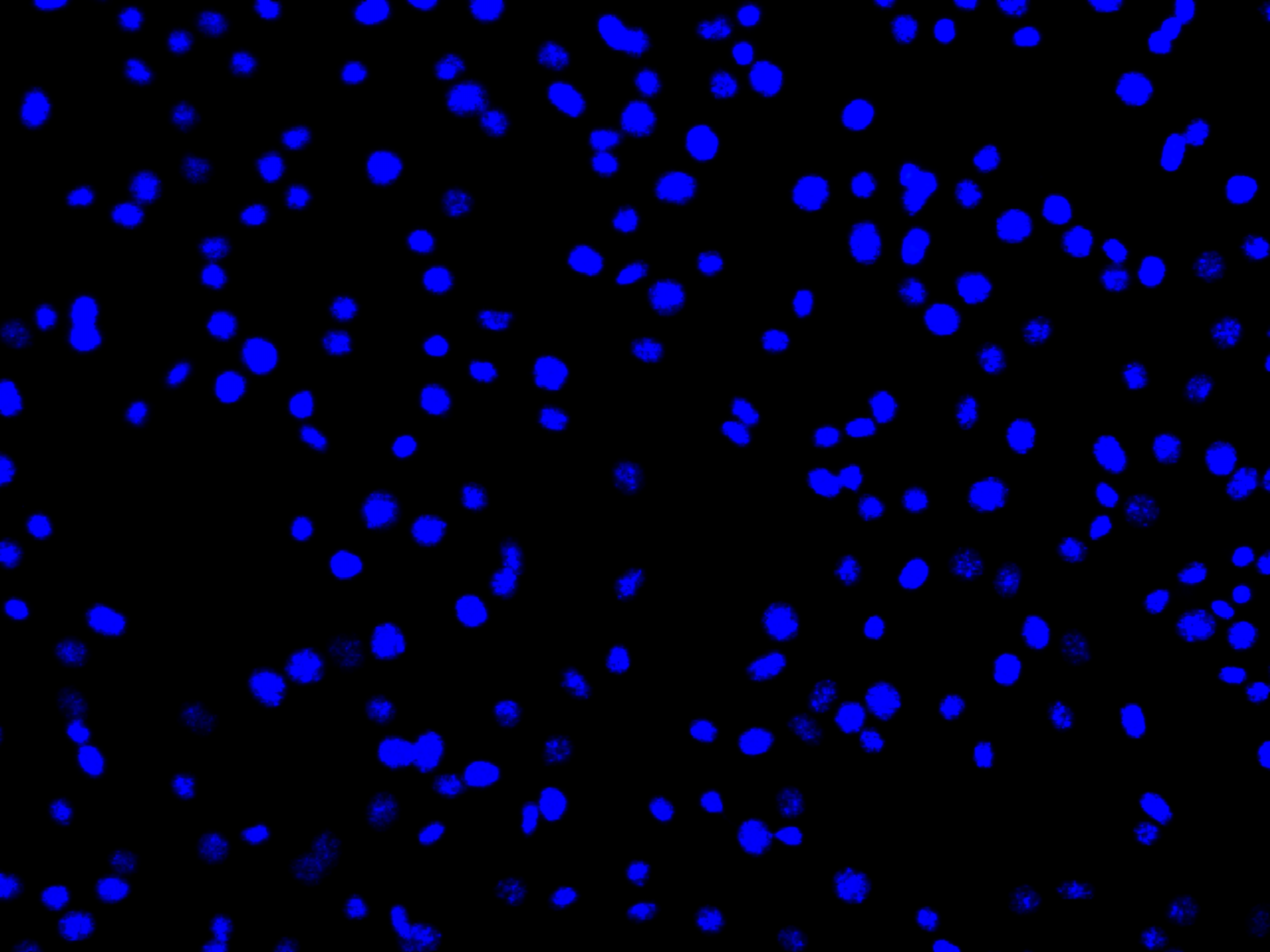

Supplement: S1 File — This zip file (fluorescence.zip) contains the original files of fluorescence imaging experiments. (ZIP) [file pone.0340382.s001.zip › fluorescence/NLPR3 fluorescence/NLRP3/HR/1_image_A.tif]

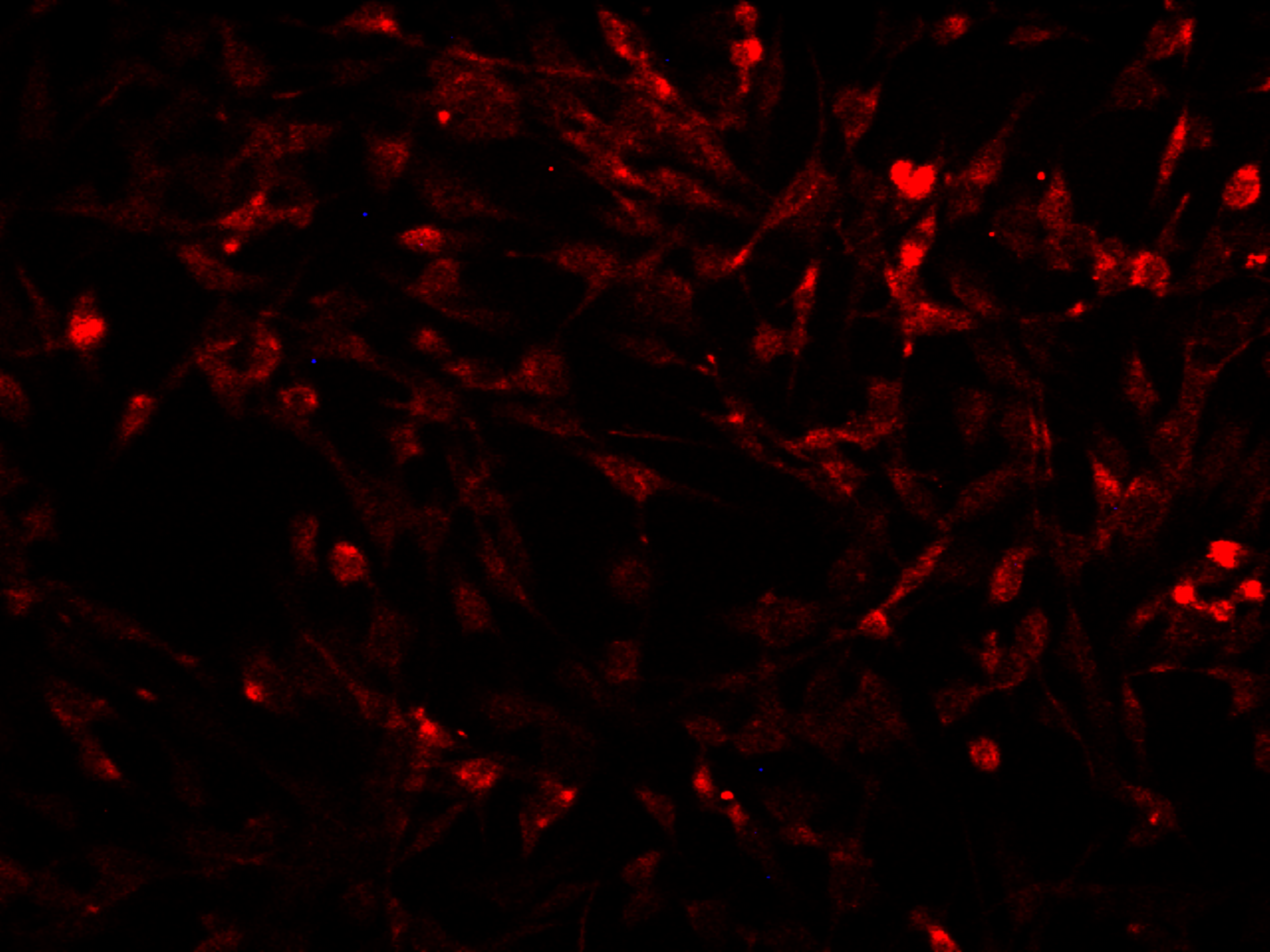

Supplement: S1 File — This zip file (fluorescence.zip) contains the original files of fluorescence imaging experiments. (ZIP) [file pone.0340382.s001.zip › fluorescence/NLPR3 fluorescence/NLRP3/HR/3_image_N21.tif]

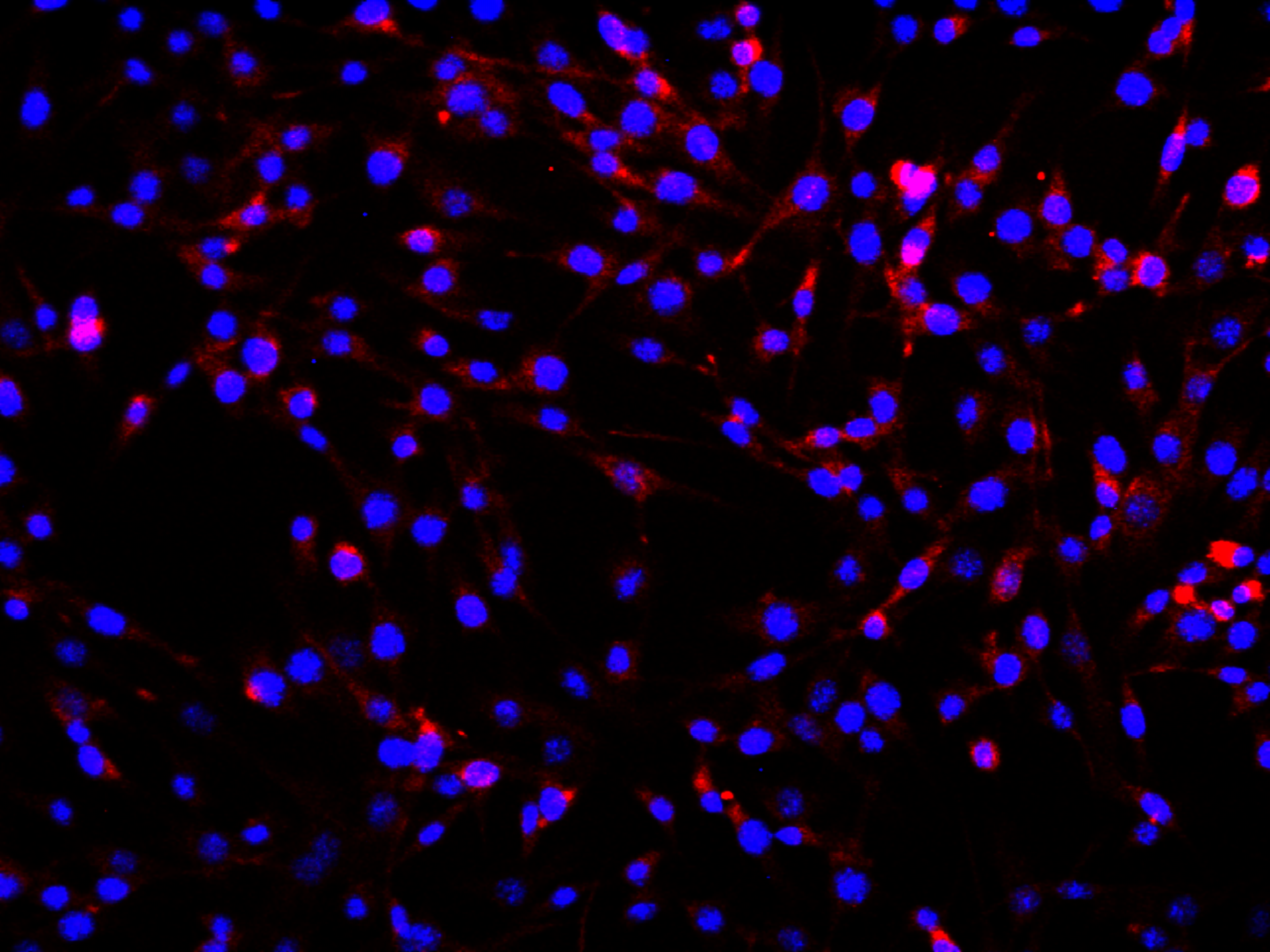

Supplement: S1 File — This zip file (fluorescence.zip) contains the original files of fluorescence imaging experiments. (ZIP) [file pone.0340382.s001.zip › fluorescence/NLPR3 fluorescence/NLRP3/HR/Overlay_Maximum.tif]

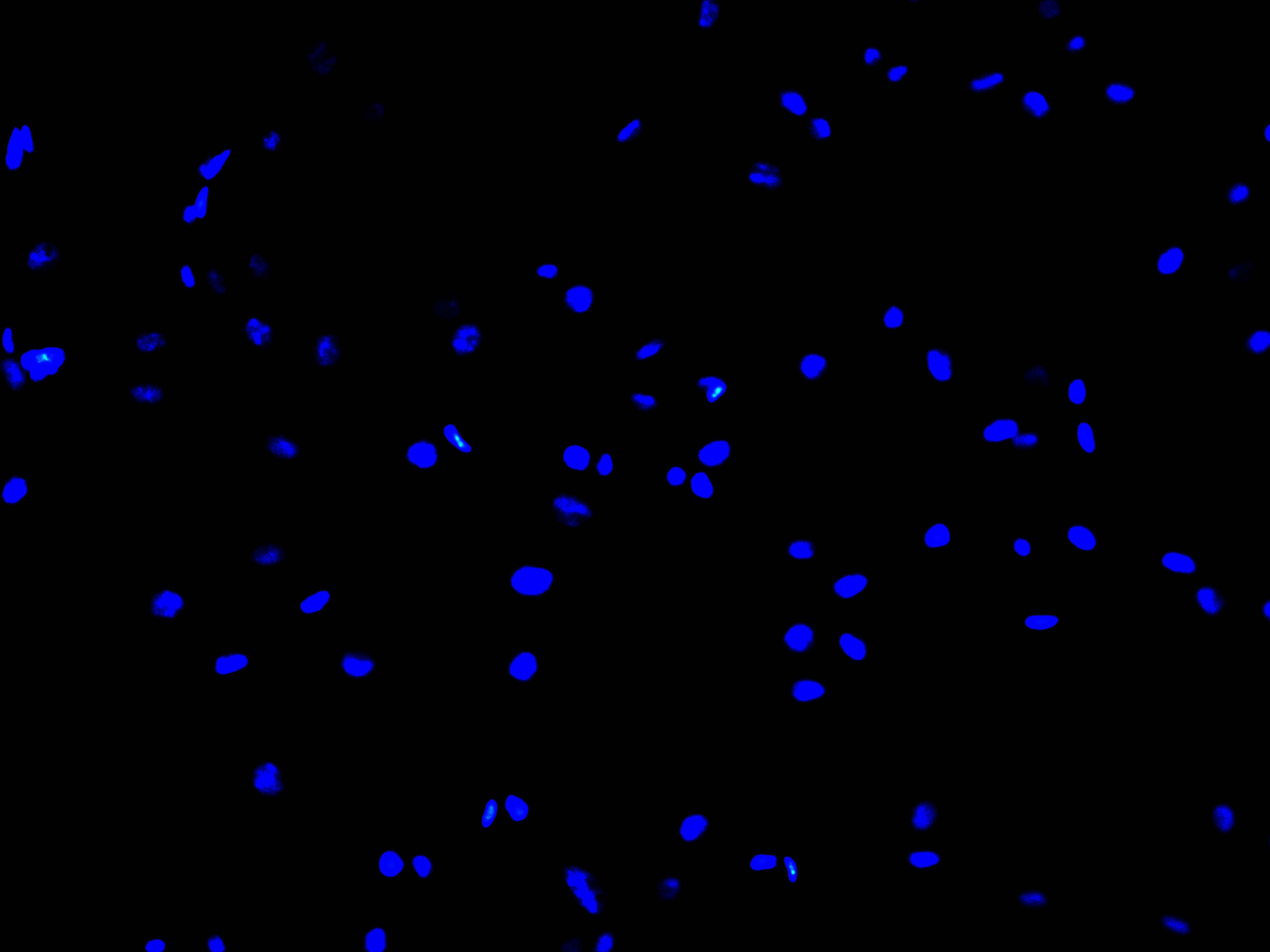

Supplement: S1 File — This zip file (fluorescence.zip) contains the original files of fluorescence imaging experiments. (ZIP) [file pone.0340382.s001.zip › fluorescence/NLPR3 fluorescence/NLRP3/NC+PDTC/1_image_A.tif]

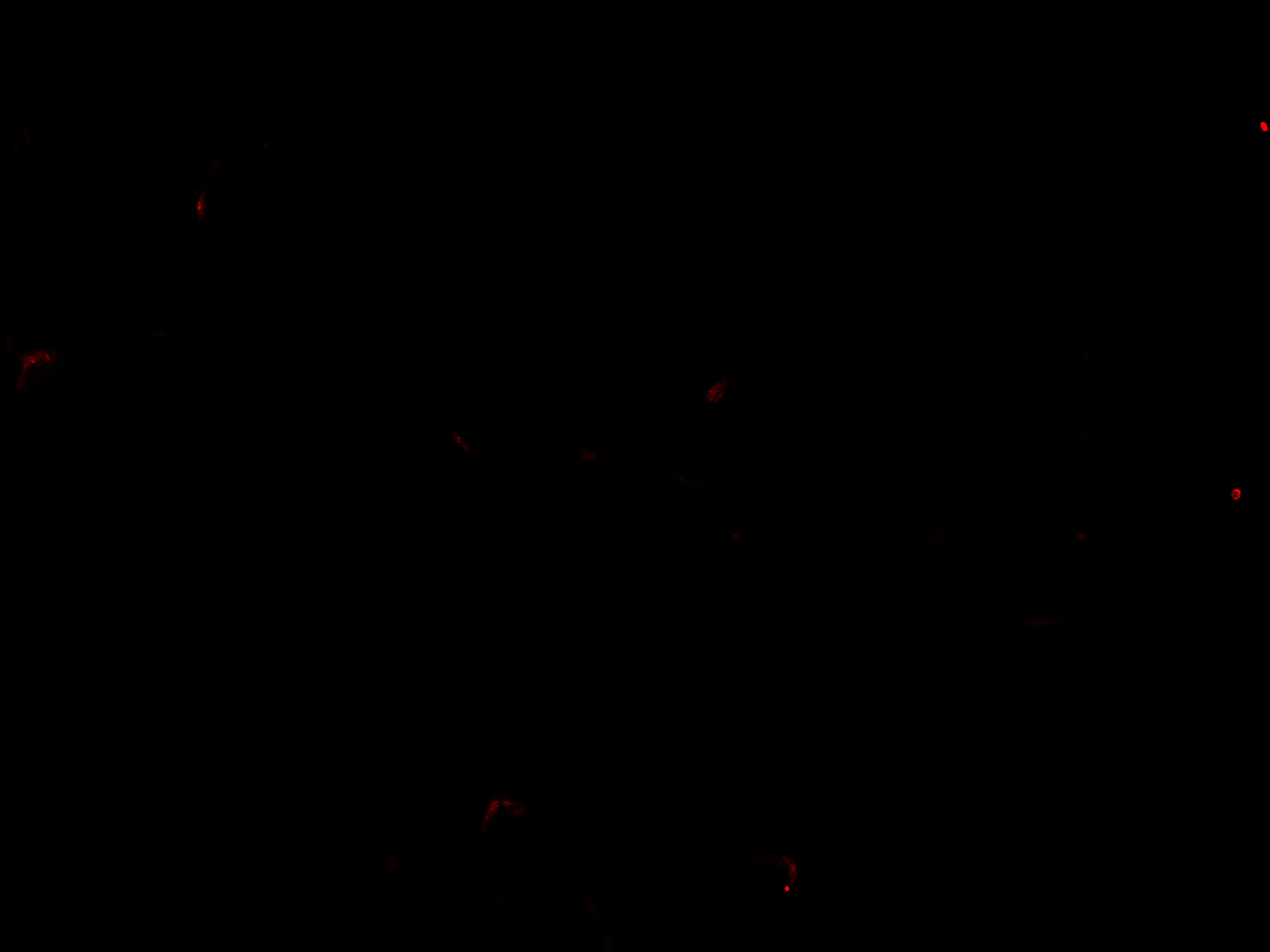

Supplement: S1 File — This zip file (fluorescence.zip) contains the original files of fluorescence imaging experiments. (ZIP) [file pone.0340382.s001.zip › fluorescence/NLPR3 fluorescence/NLRP3/NC+PDTC/3_image_N21.tif]

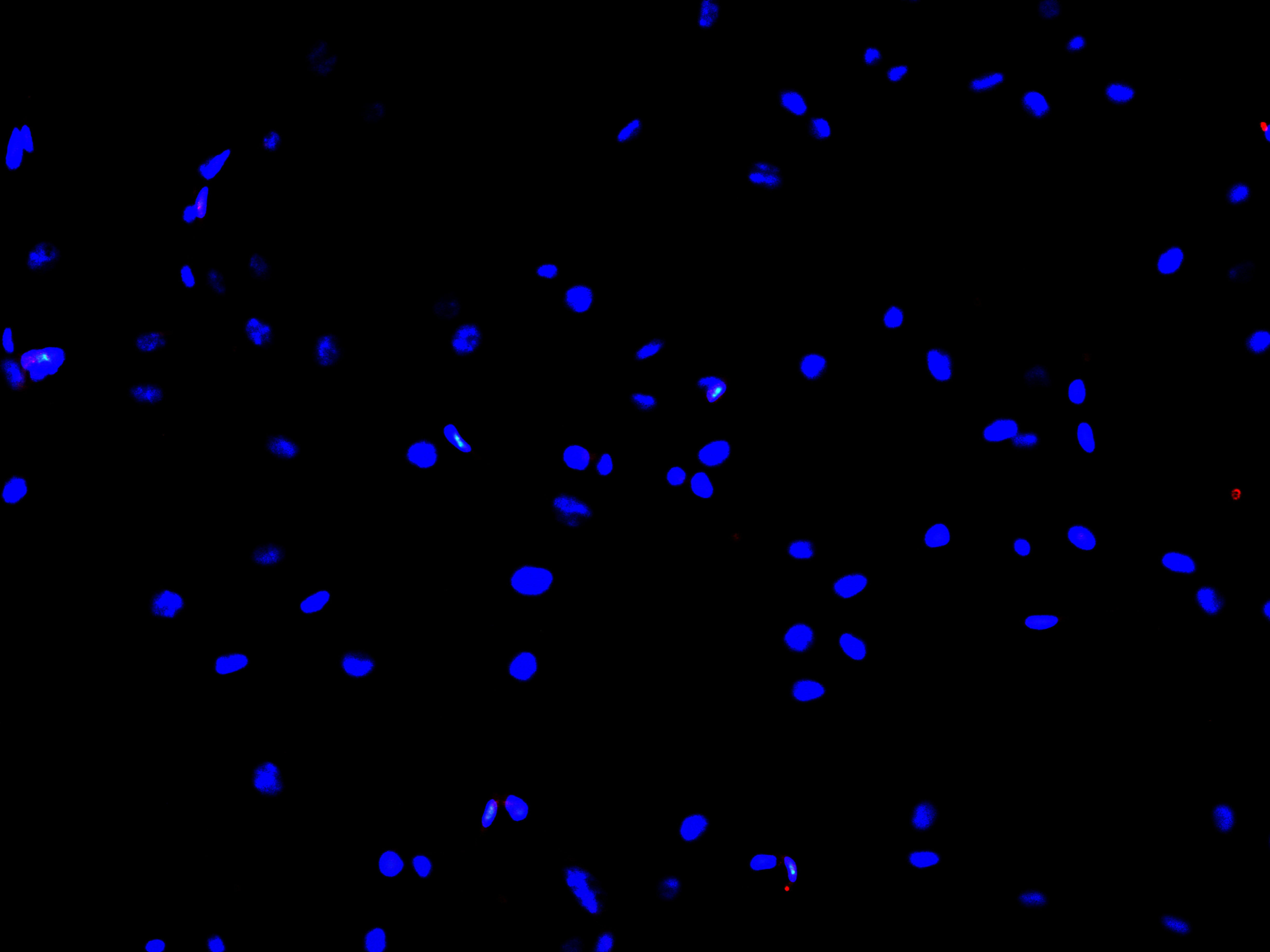

Supplement: S1 File — This zip file (fluorescence.zip) contains the original files of fluorescence imaging experiments. (ZIP) [file pone.0340382.s001.zip › fluorescence/NLPR3 fluorescence/NLRP3/NC+PDTC/Overlay_Maximum.tif]

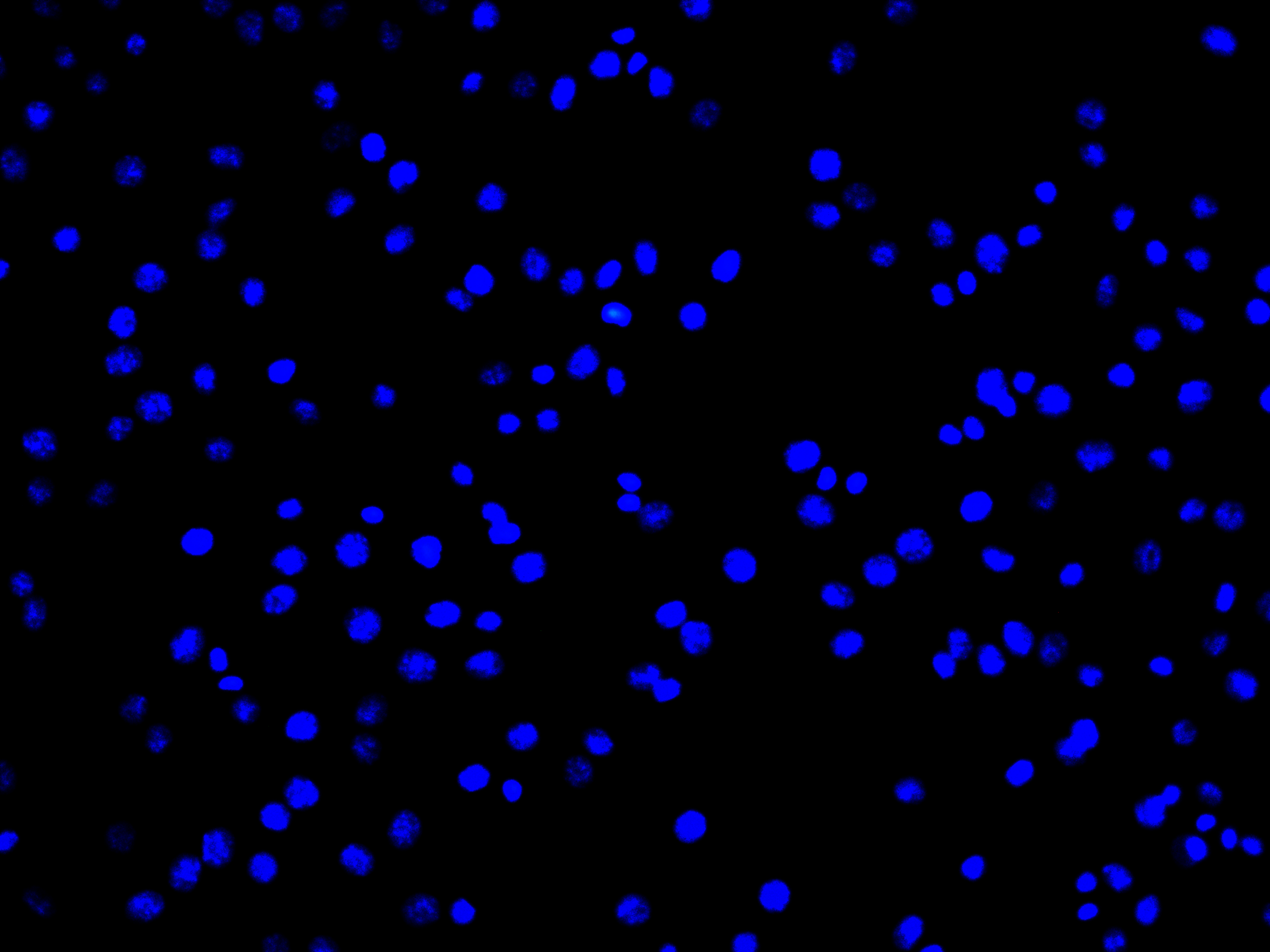

Supplement: S1 File — This zip file (fluorescence.zip) contains the original files of fluorescence imaging experiments. (ZIP) [file pone.0340382.s001.zip › fluorescence/NLPR3 fluorescence/NLRP3/NC/1_image_A.tif]

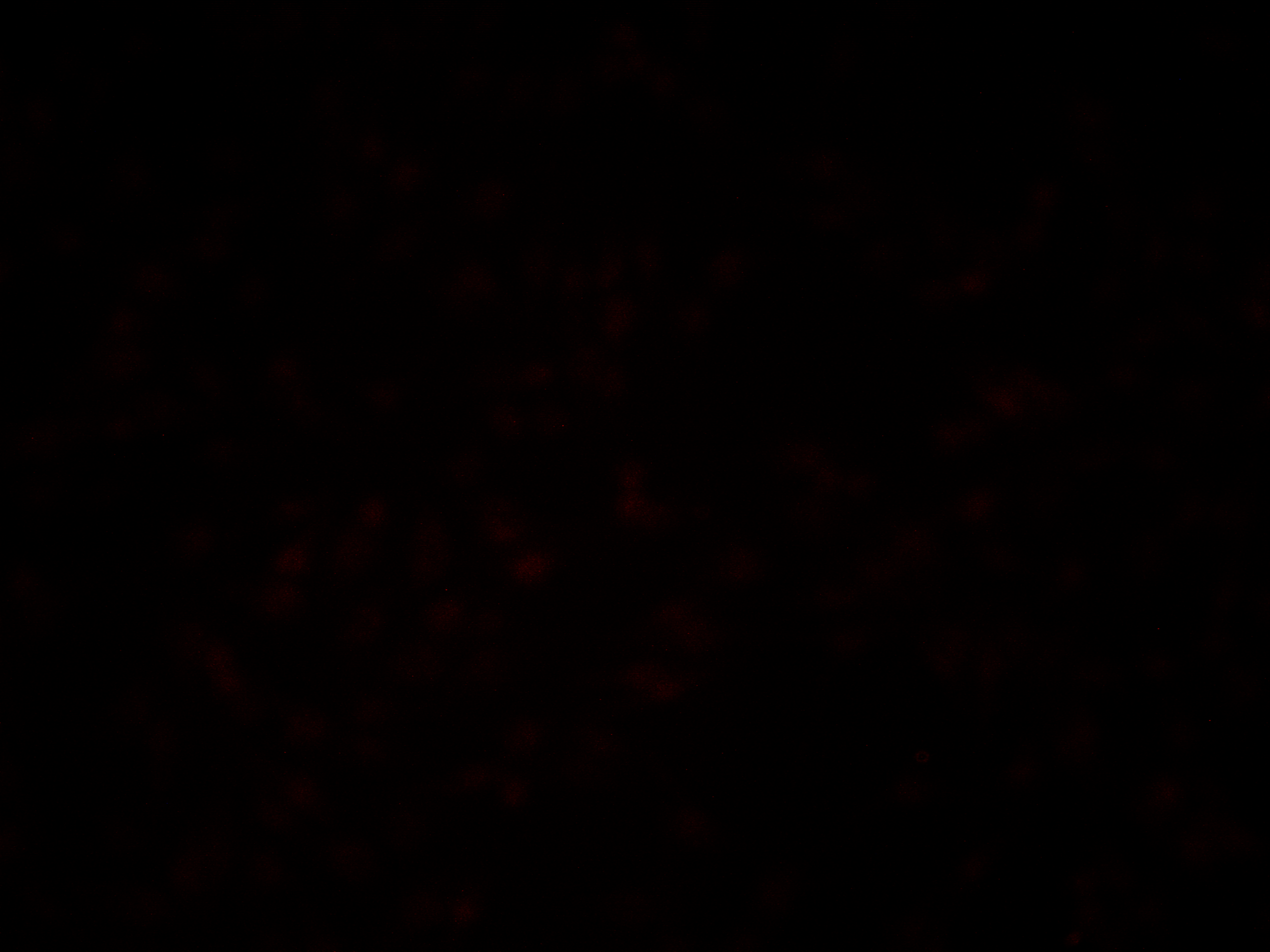

Supplement: S1 File — This zip file (fluorescence.zip) contains the original files of fluorescence imaging experiments. (ZIP) [file pone.0340382.s001.zip › fluorescence/NLPR3 fluorescence/NLRP3/NC/3_image_N21.tif]

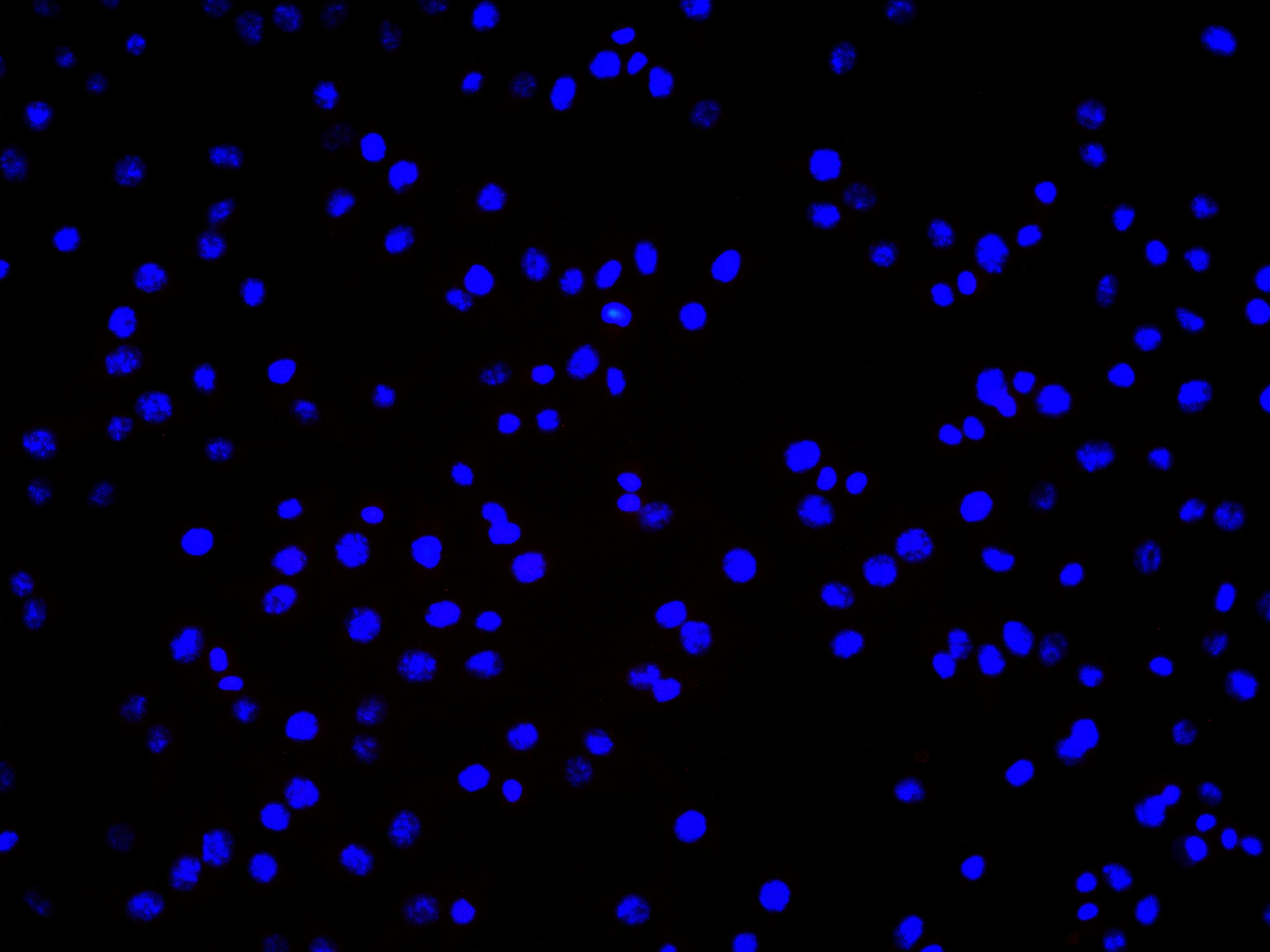

Supplement: S1 File — This zip file (fluorescence.zip) contains the original files of fluorescence imaging experiments. (ZIP) [file pone.0340382.s001.zip › fluorescence/NLPR3 fluorescence/NLRP3/NC/Overlay_Maximum.tif]

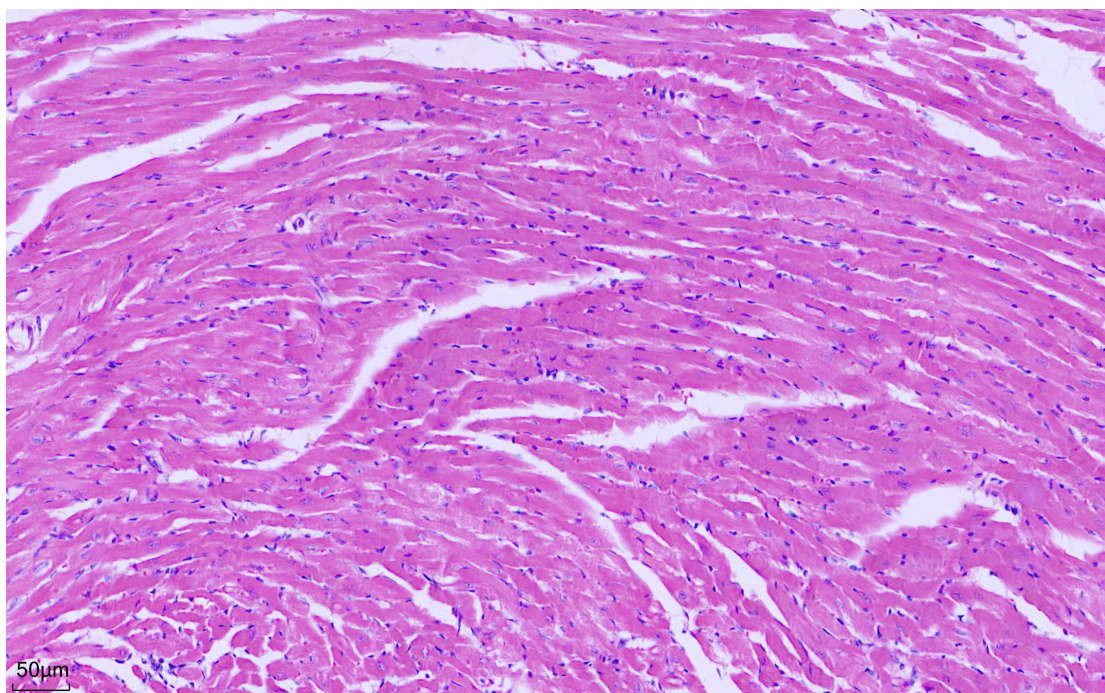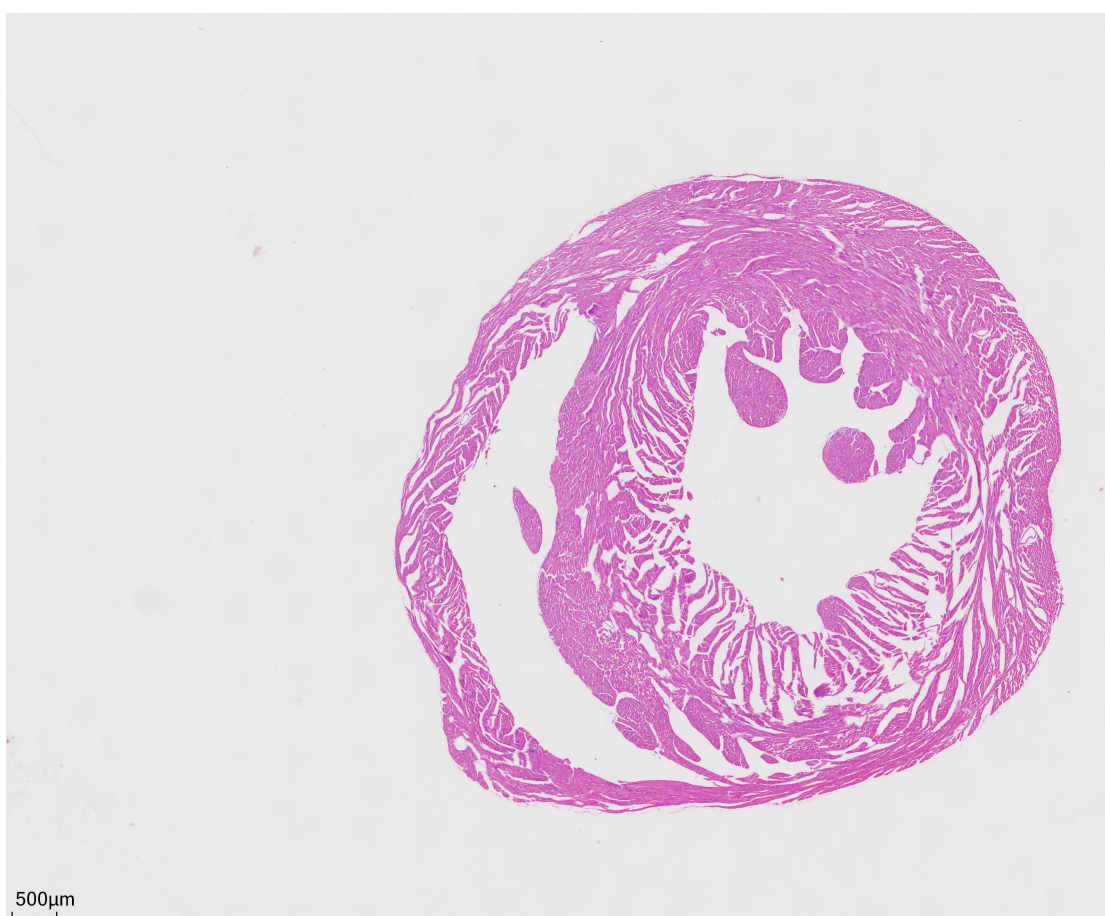

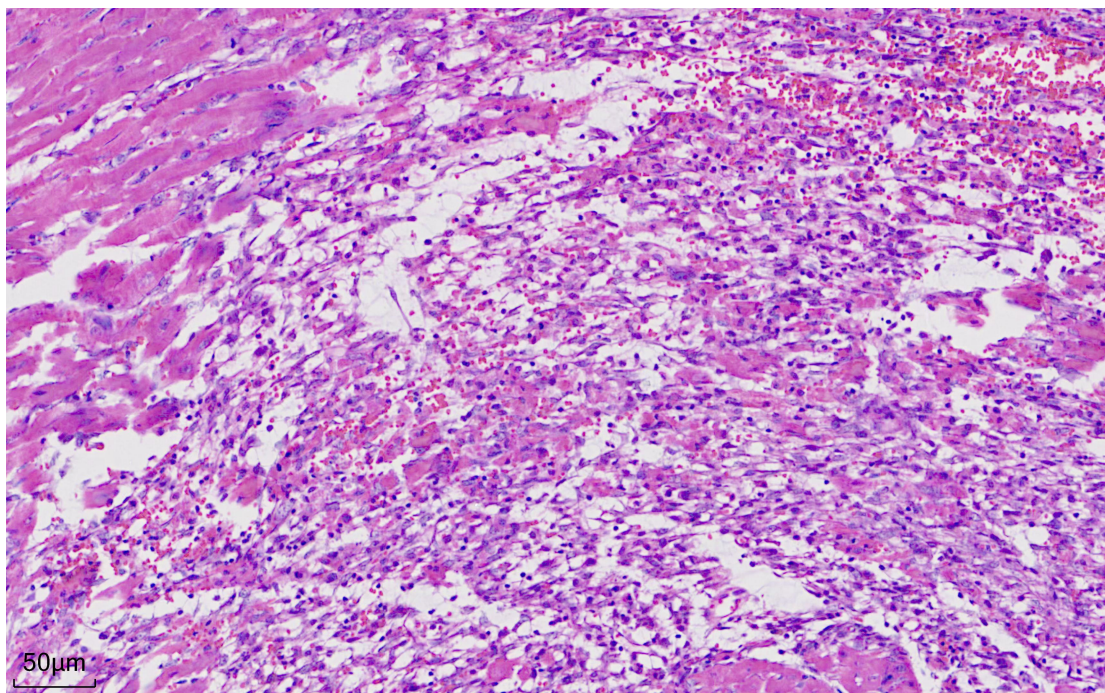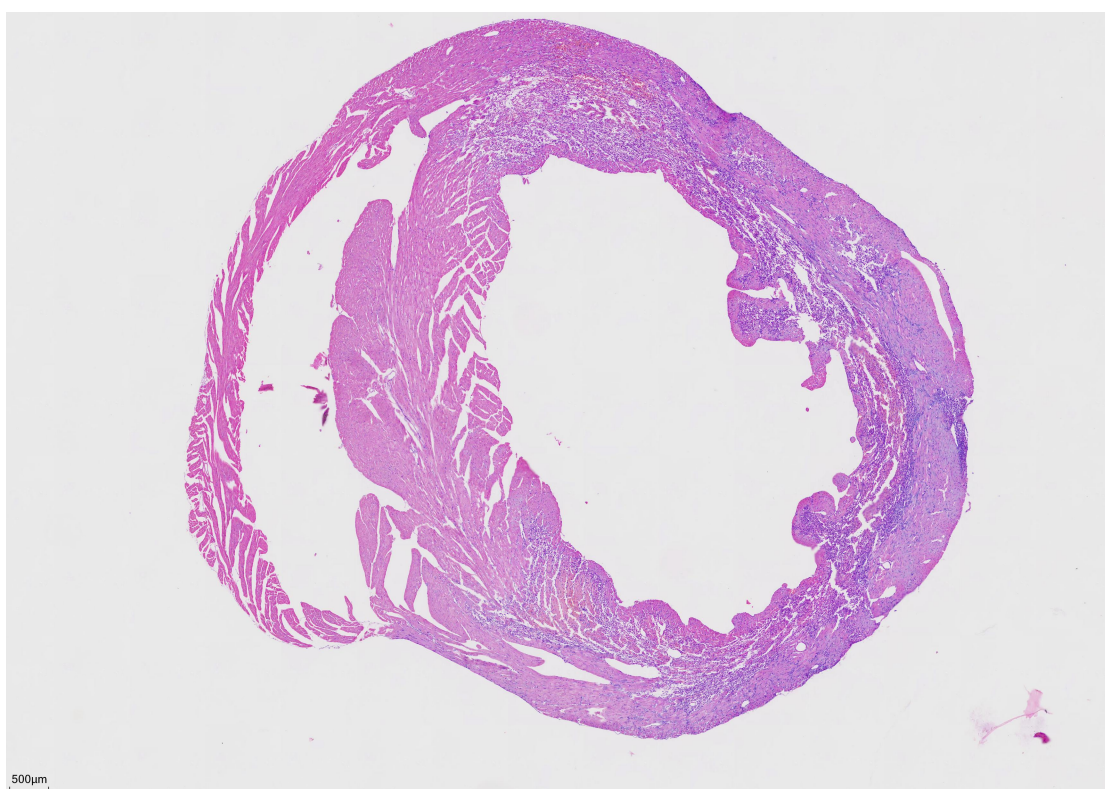

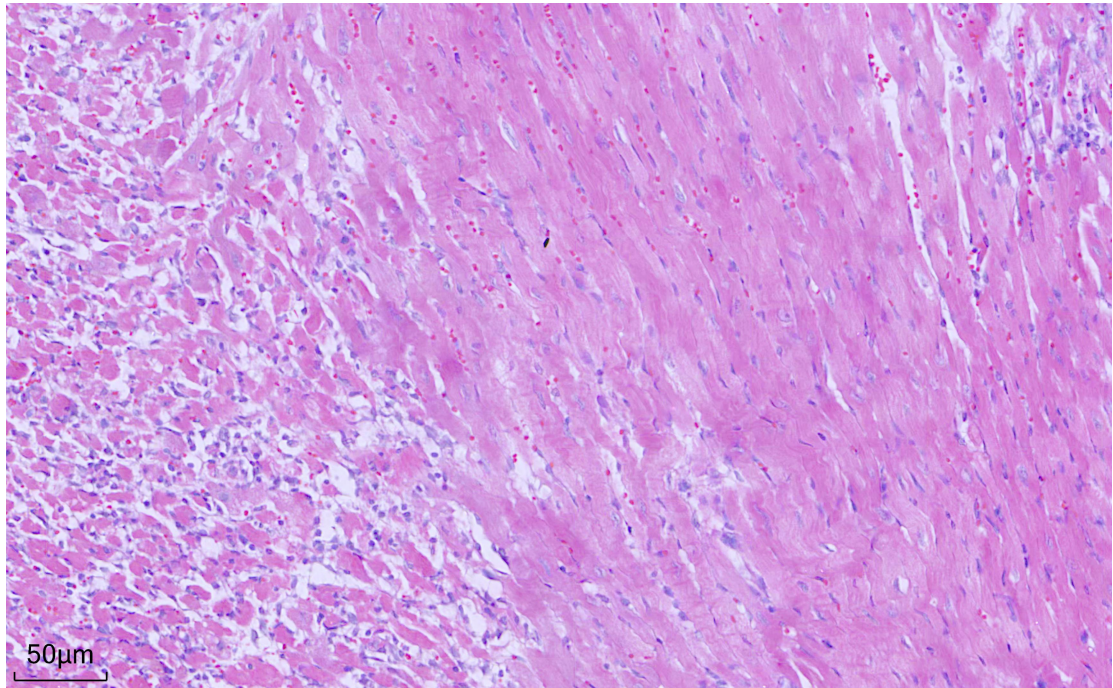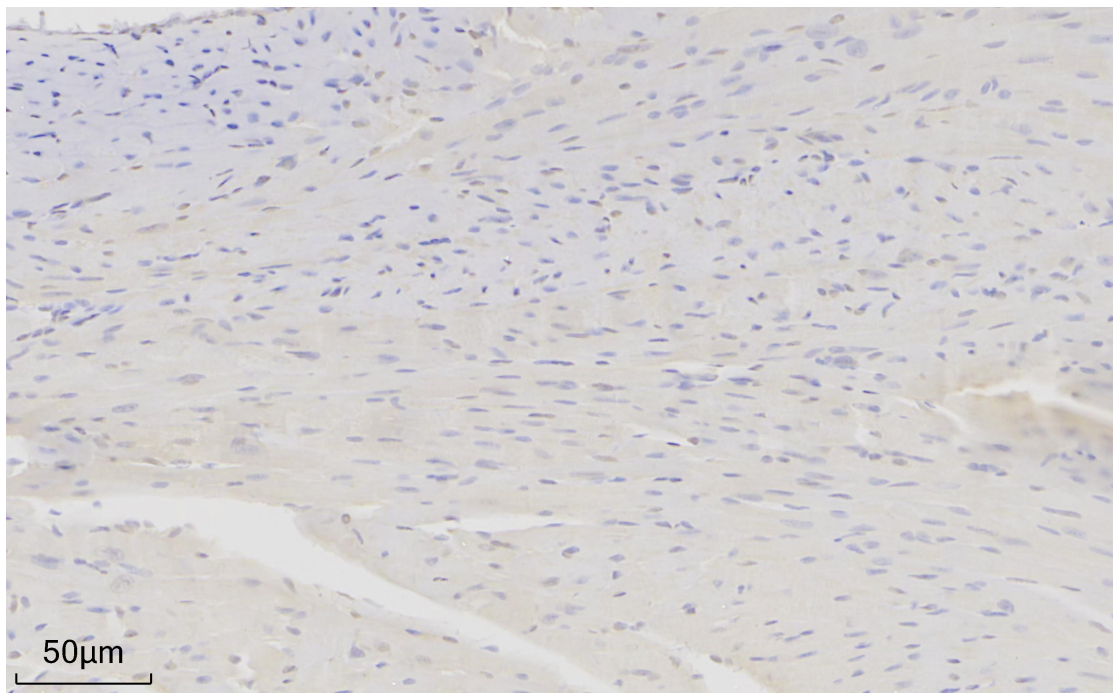

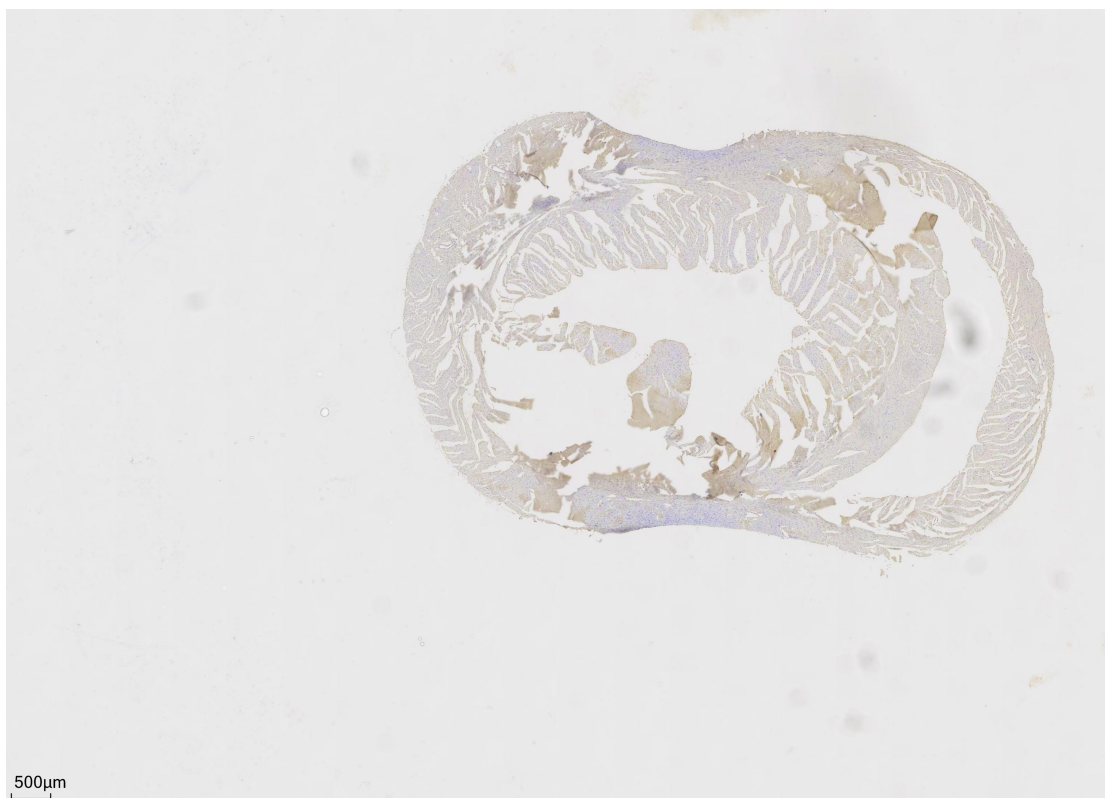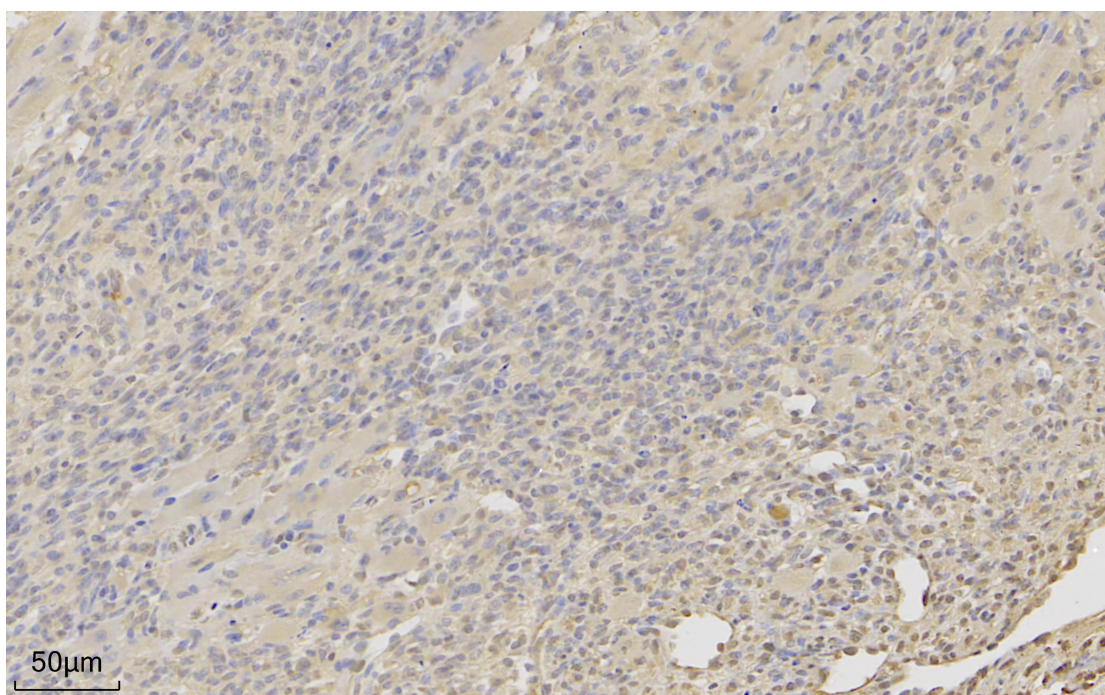

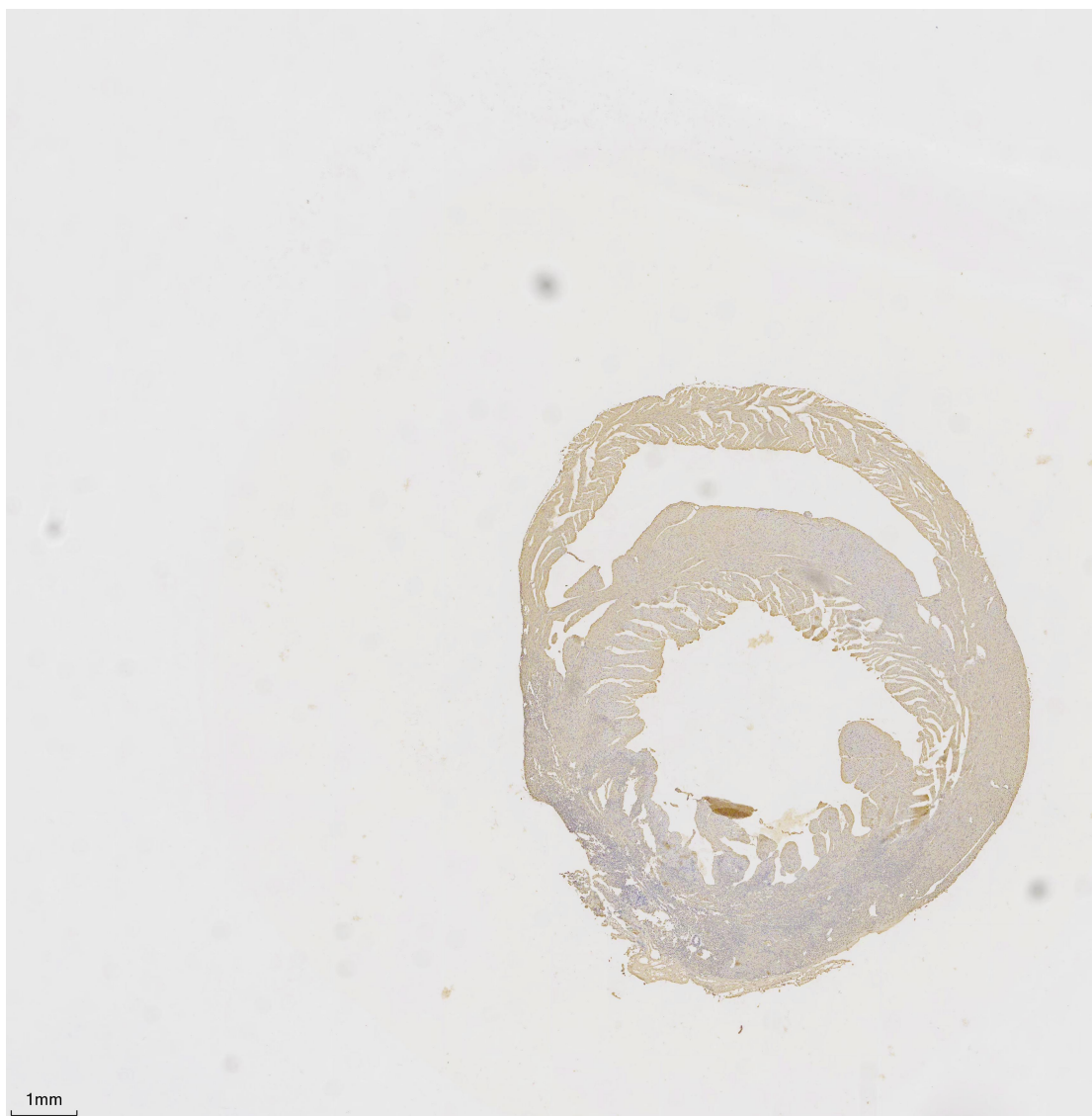

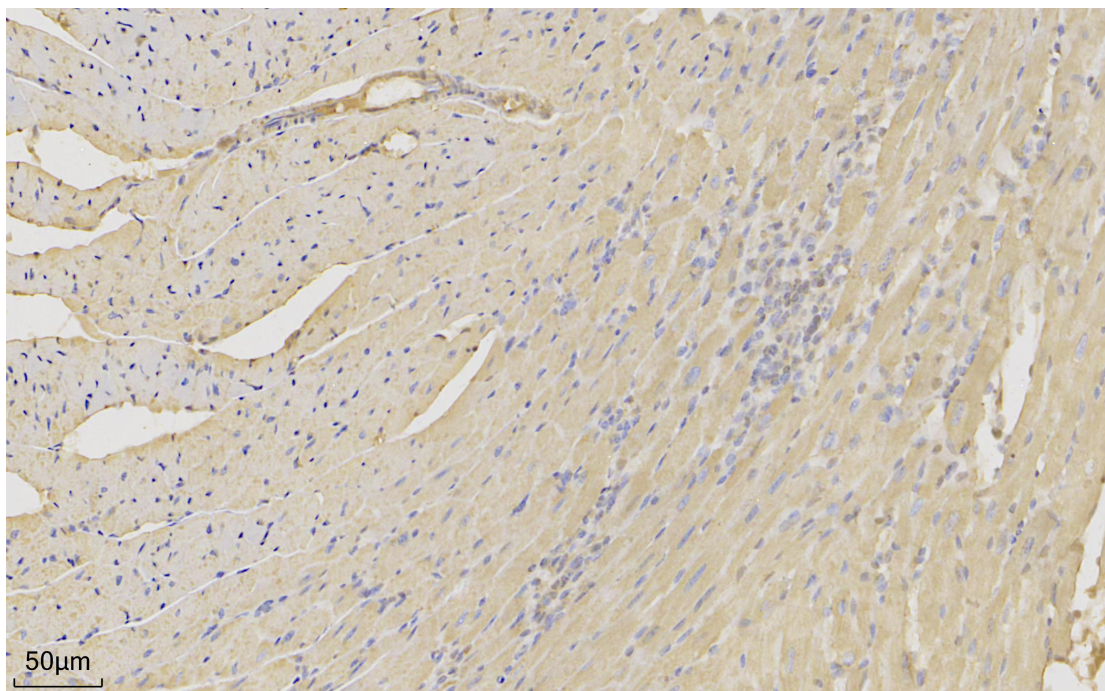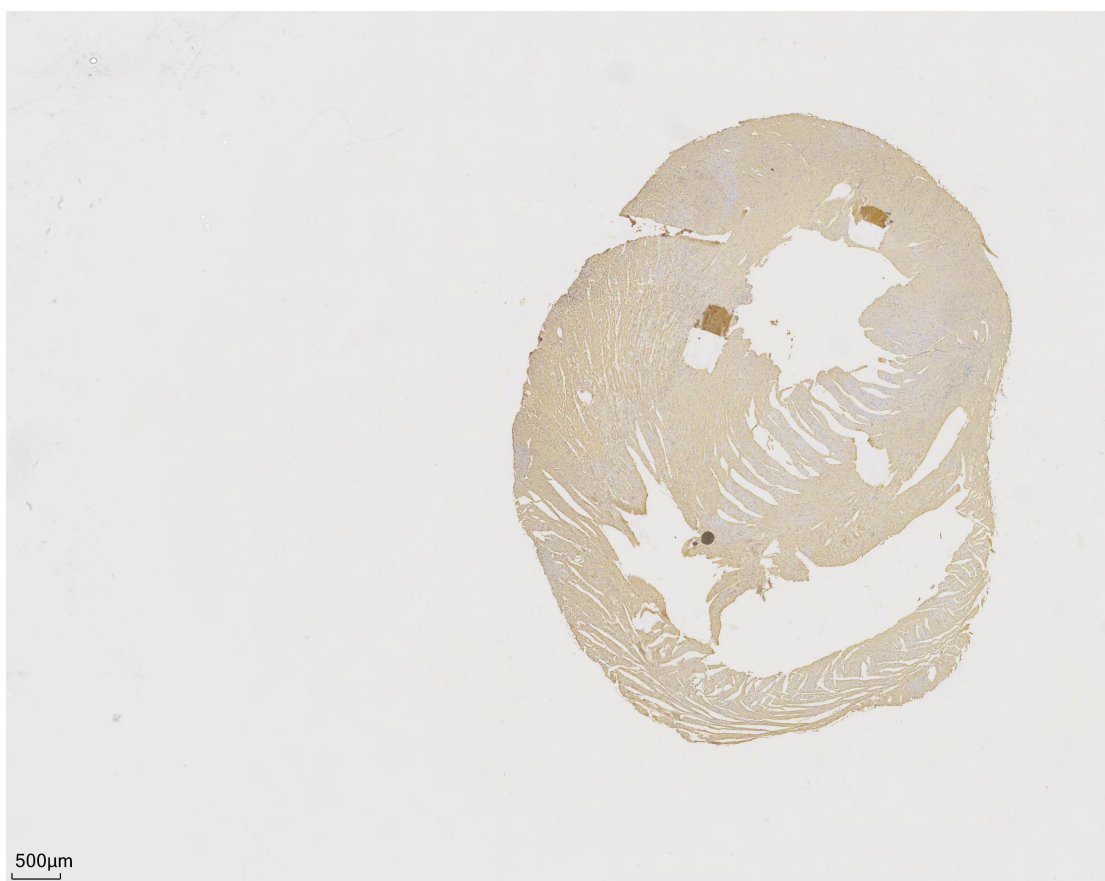

Supplement: S2 File — This zip file (C57.zip) includes the raw experimental materials related to the C57 mouse model. (ZIP) [file pone.0340382.s002.zip › C57/HE and IHC.pdf]

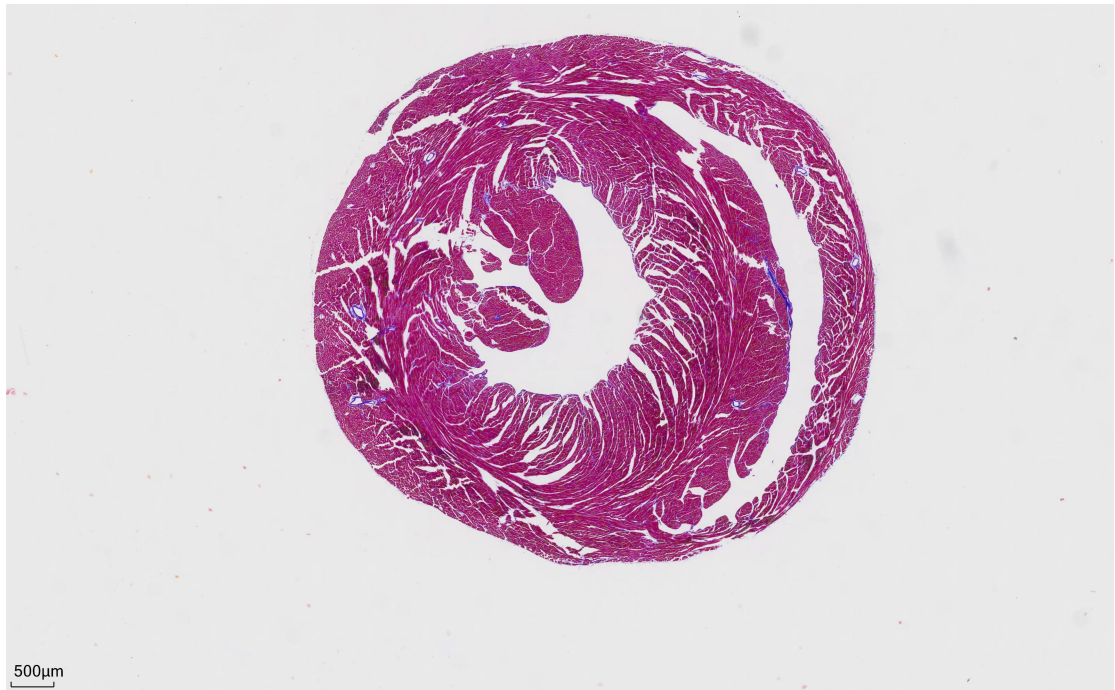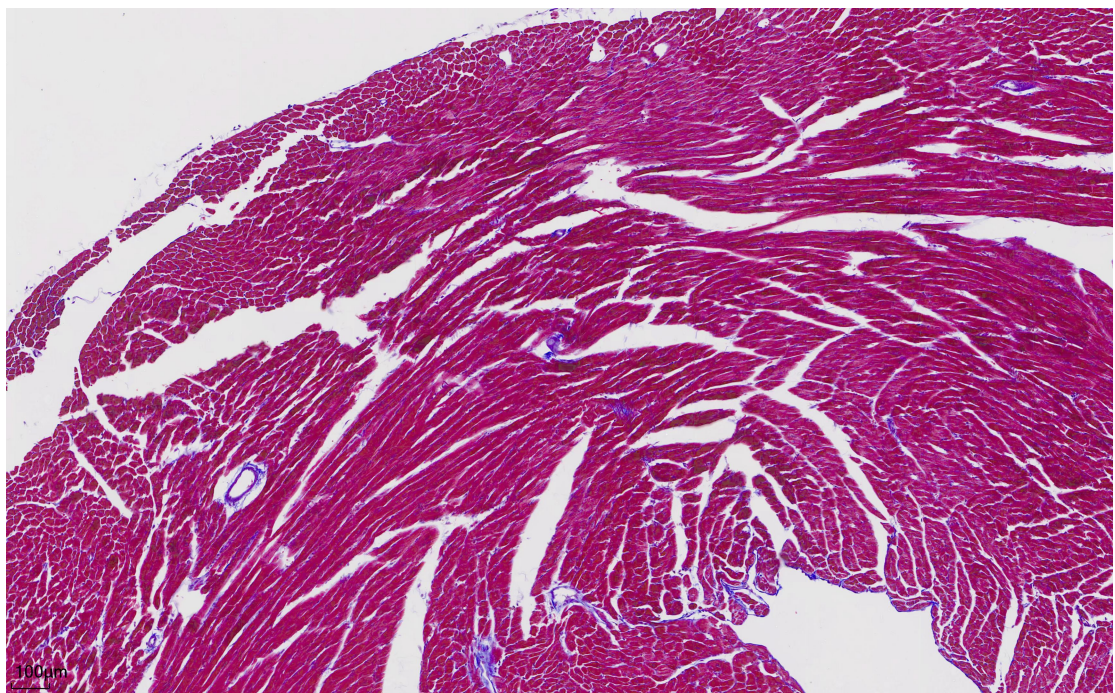

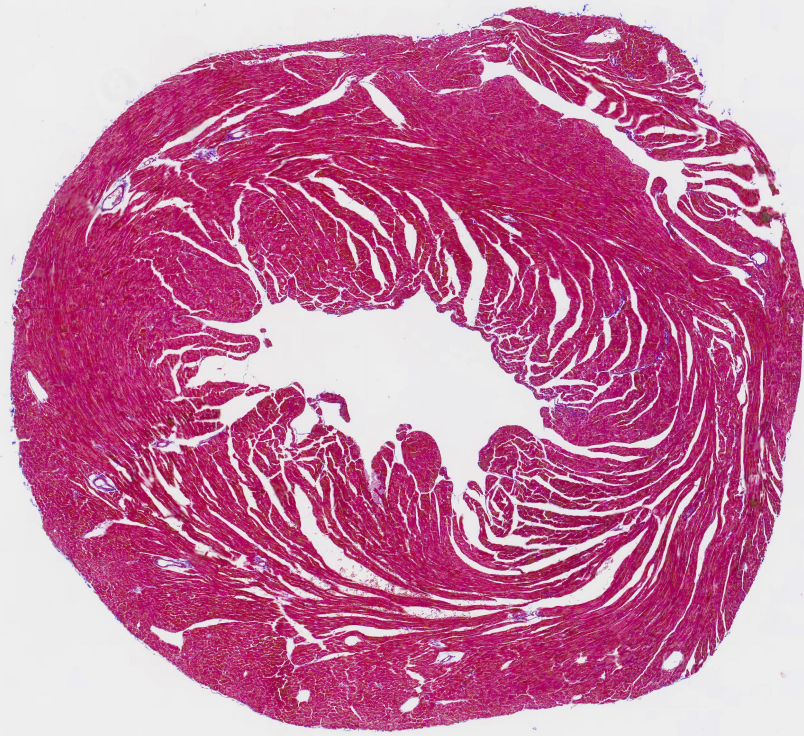

500µm

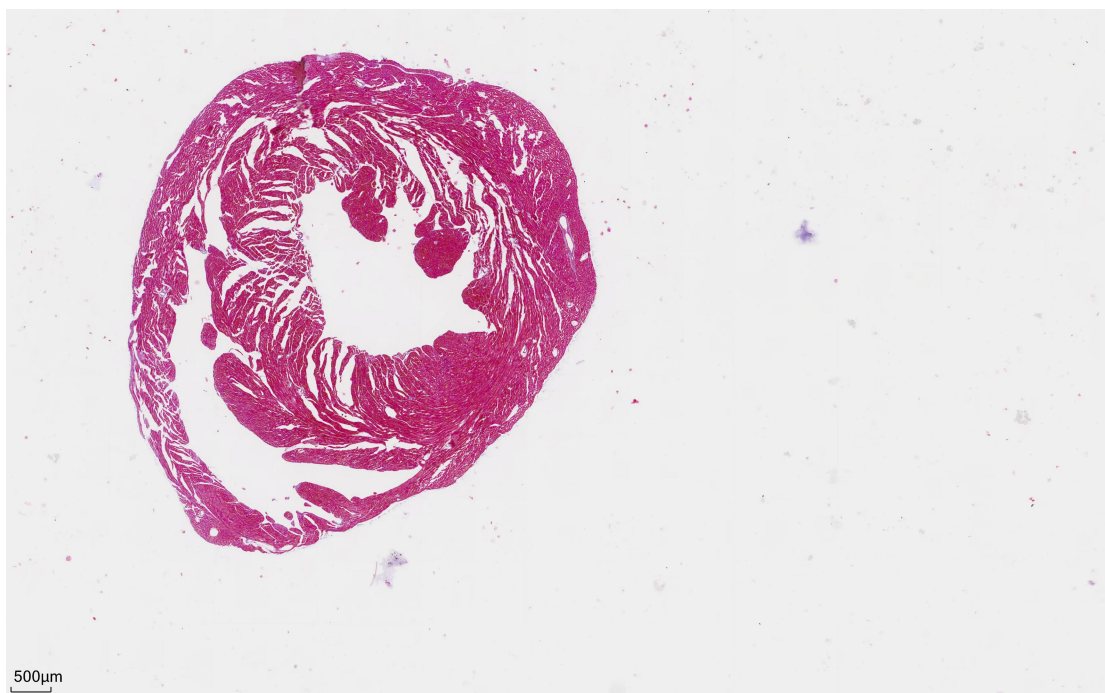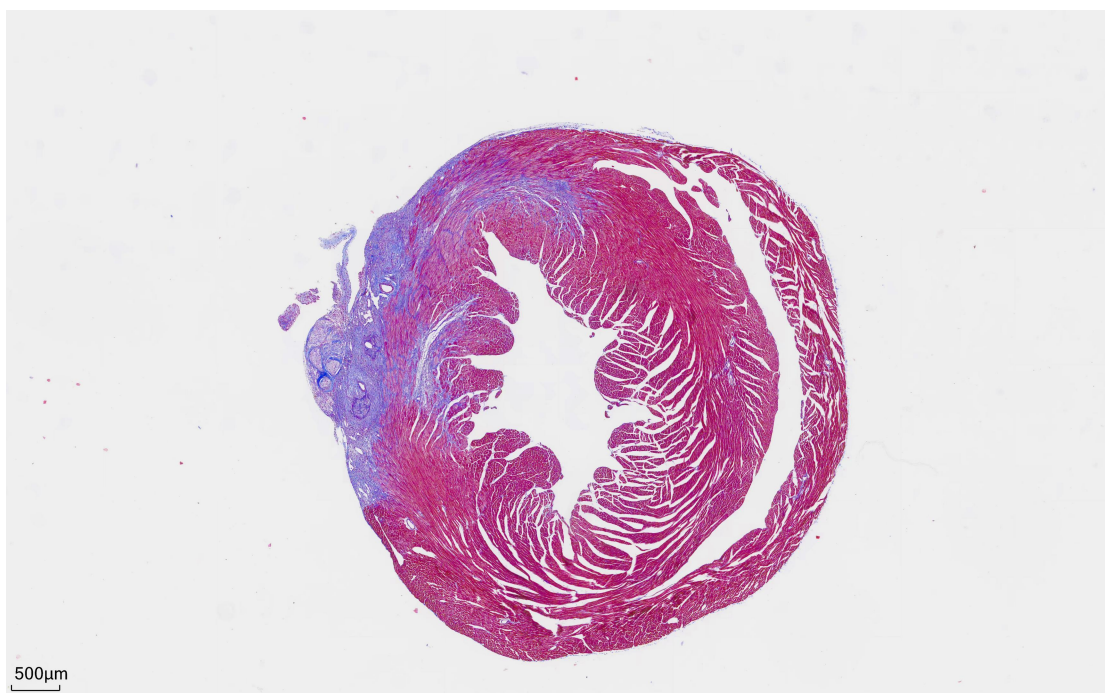

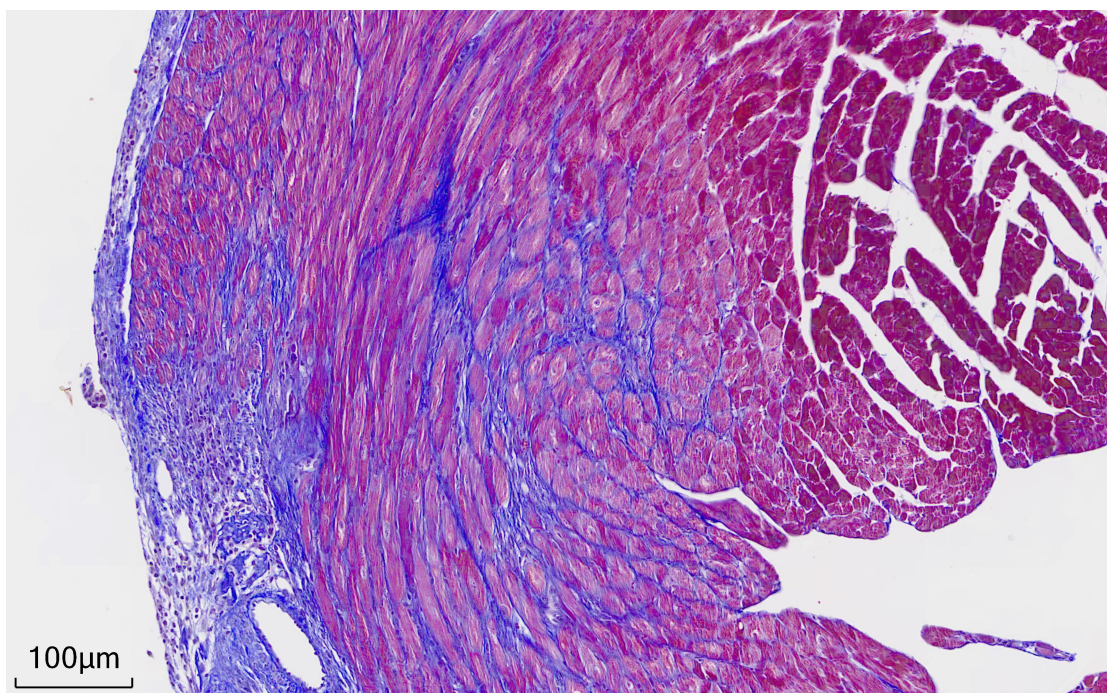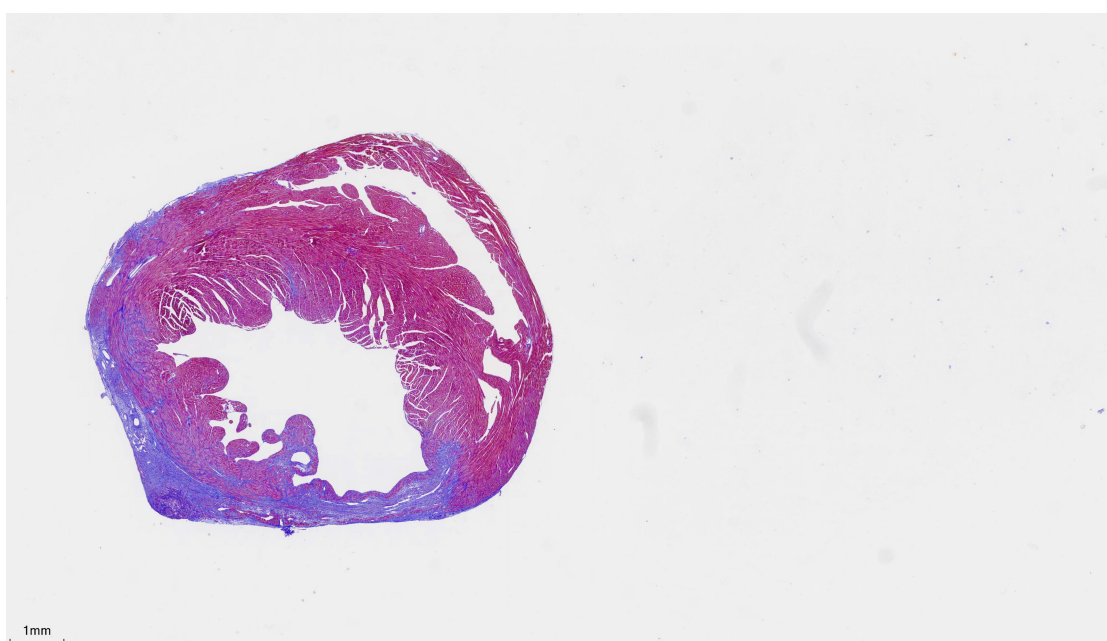

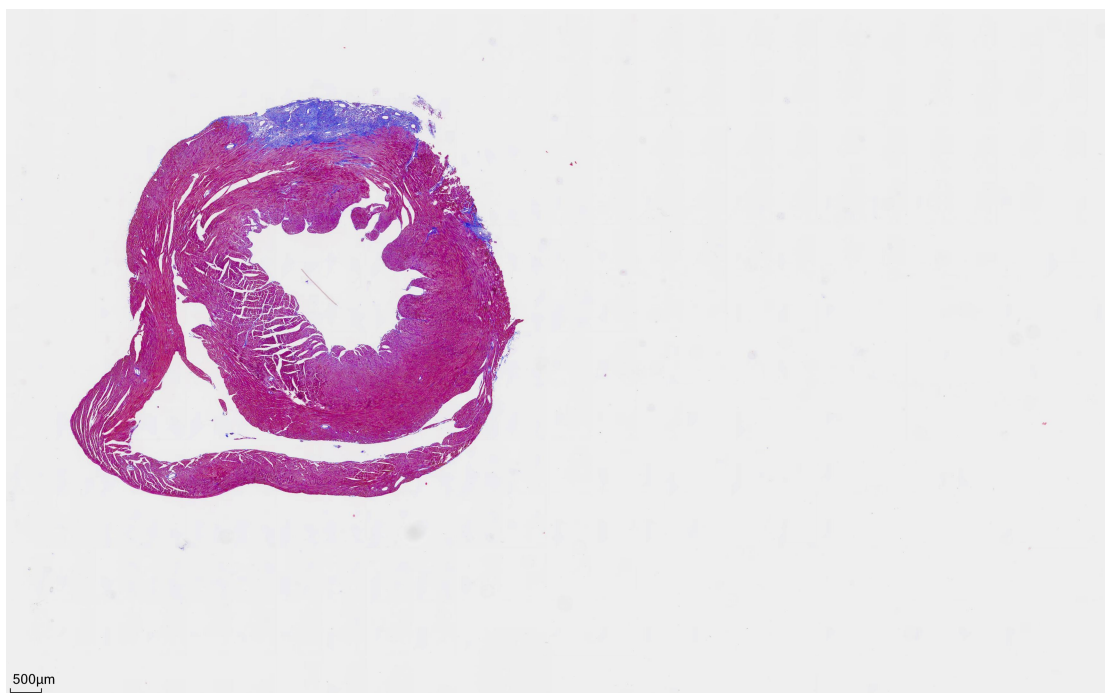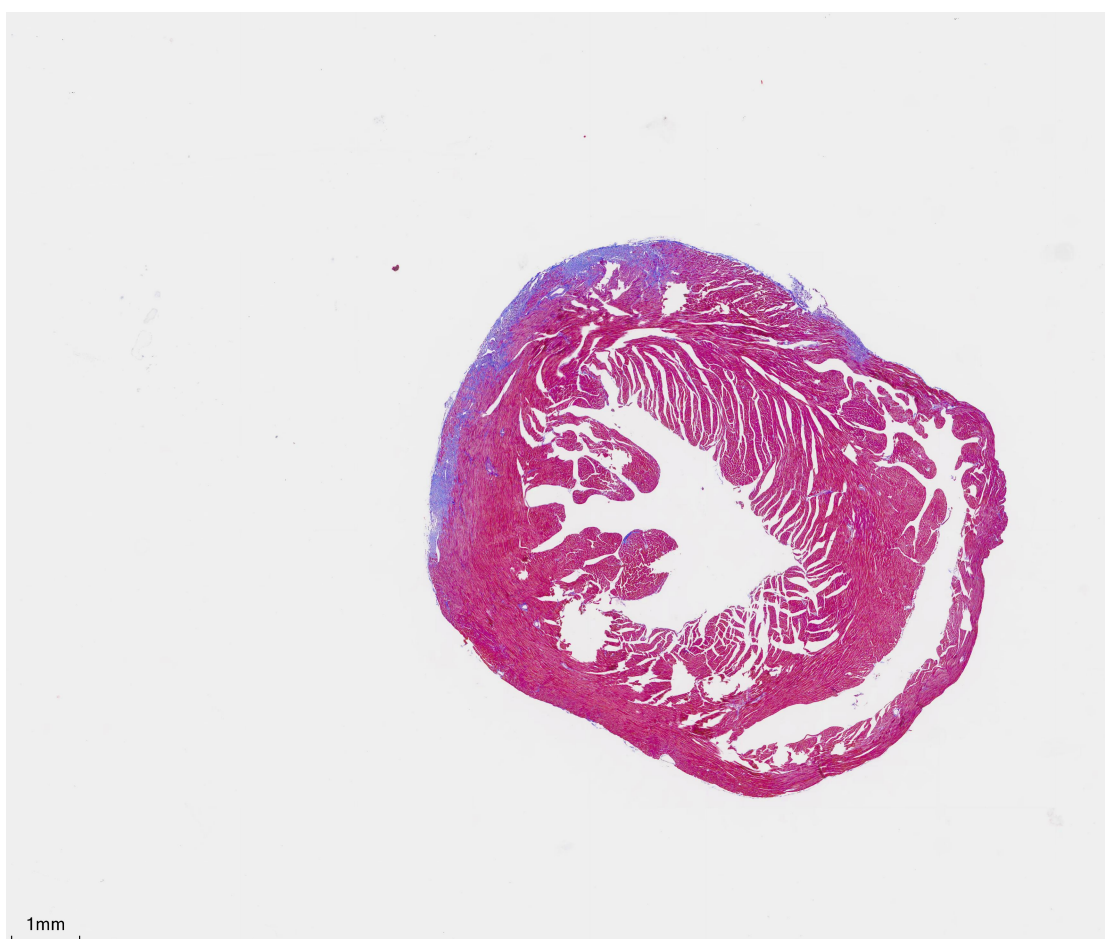

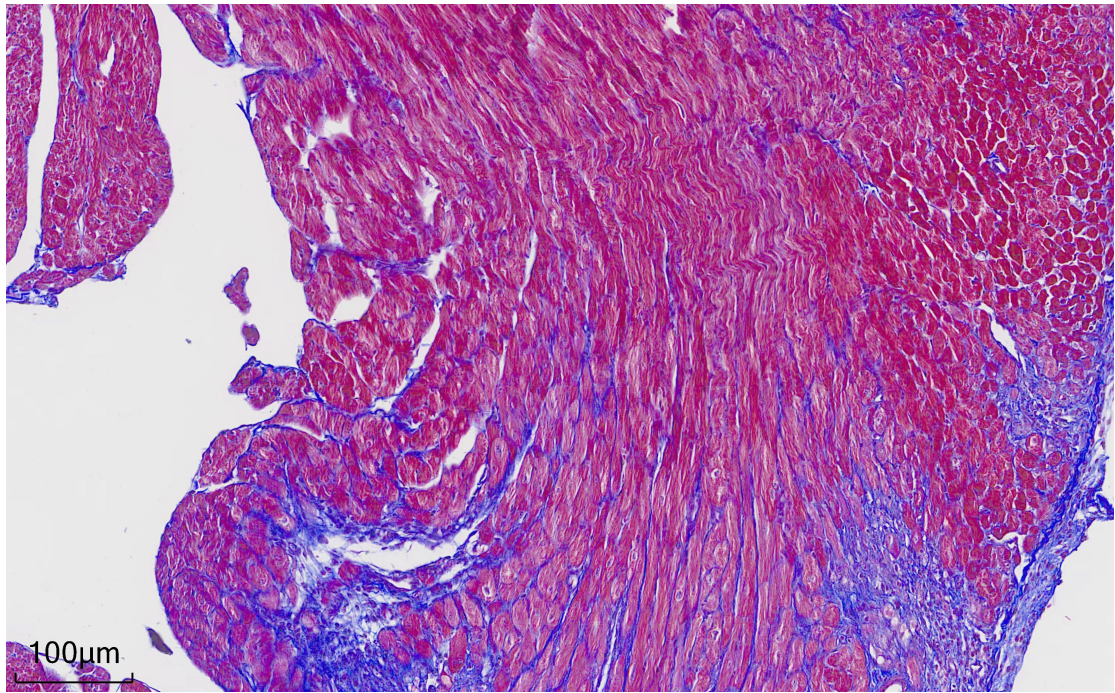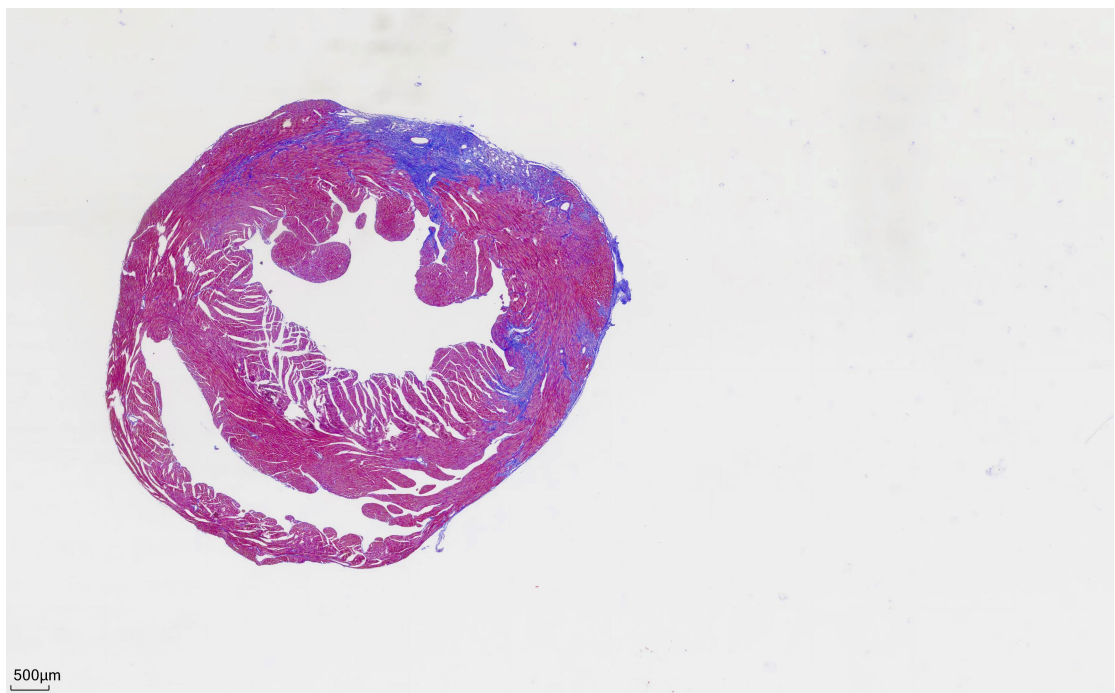

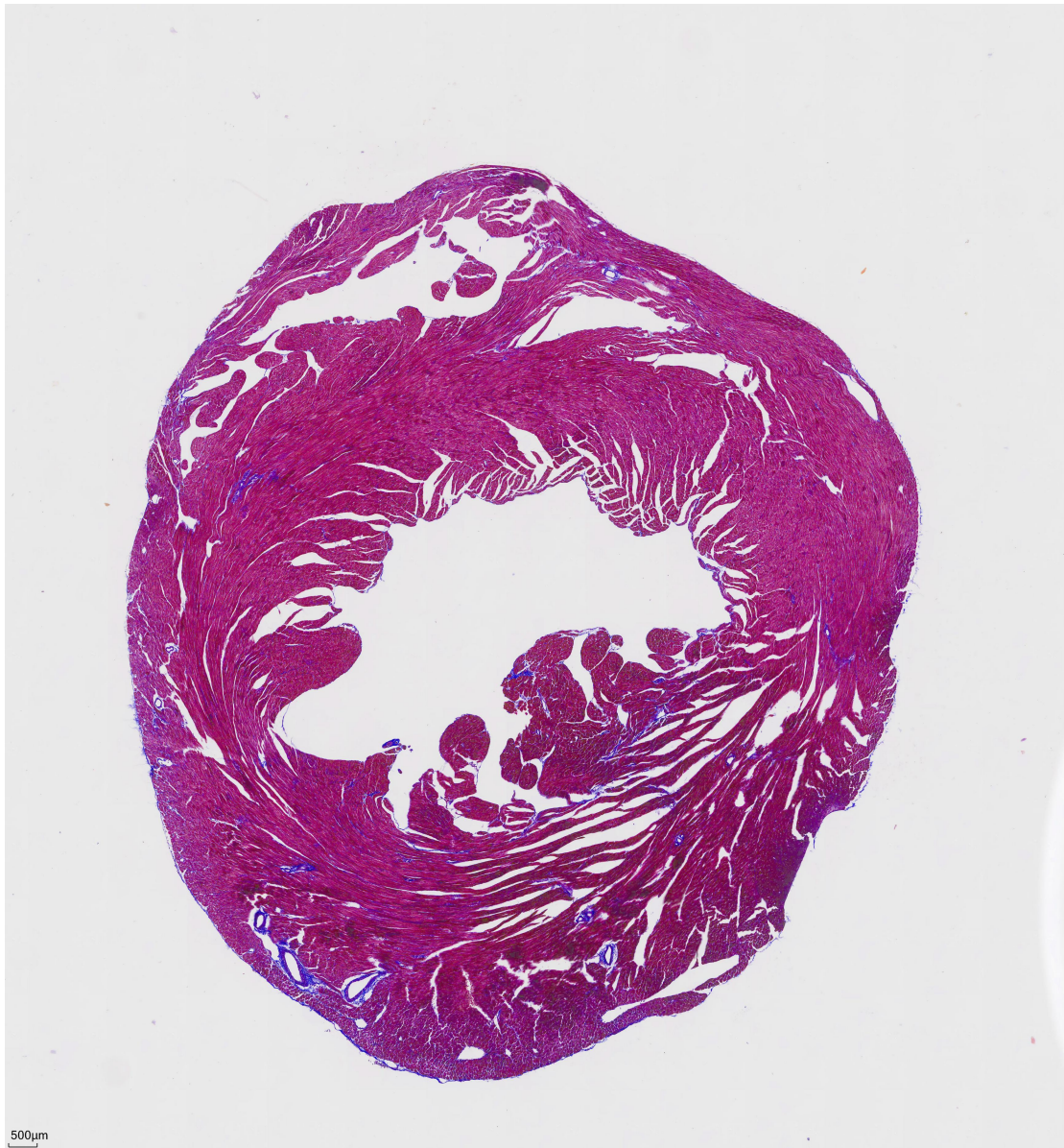

Supplement: S2 File — This zip file (C57.zip) includes the raw experimental materials related to the C57 mouse model. (ZIP) [file pone.0340382.s002.zip › C57/masson.pdf]

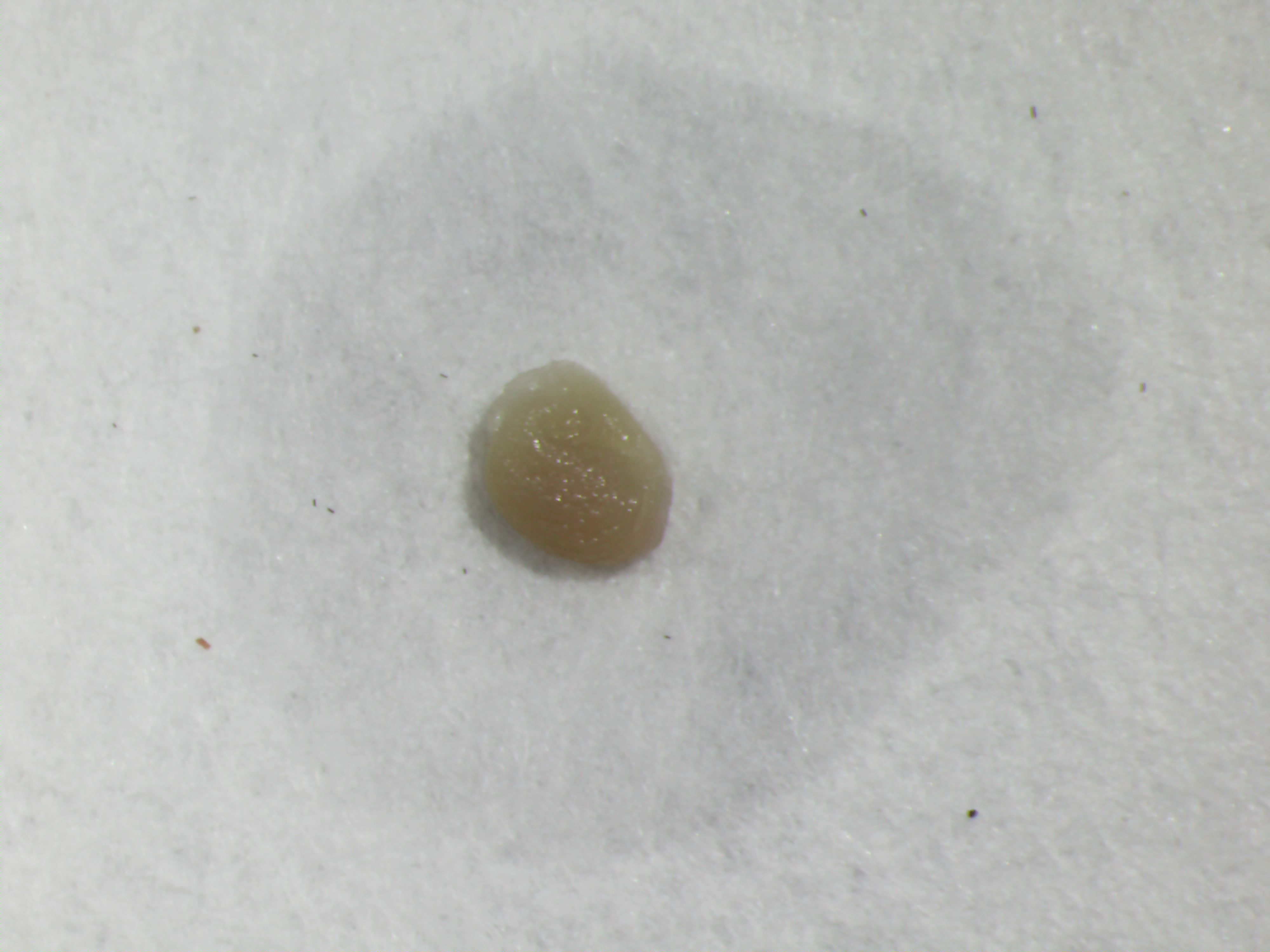

Supplement: S2 File — This zip file (C57.zip) includes the raw experimental materials related to the C57 mouse model. (ZIP) [file pone.0340382.s002.zip › C57/TTC and ultrasound/TTC/IR/1.tiff]

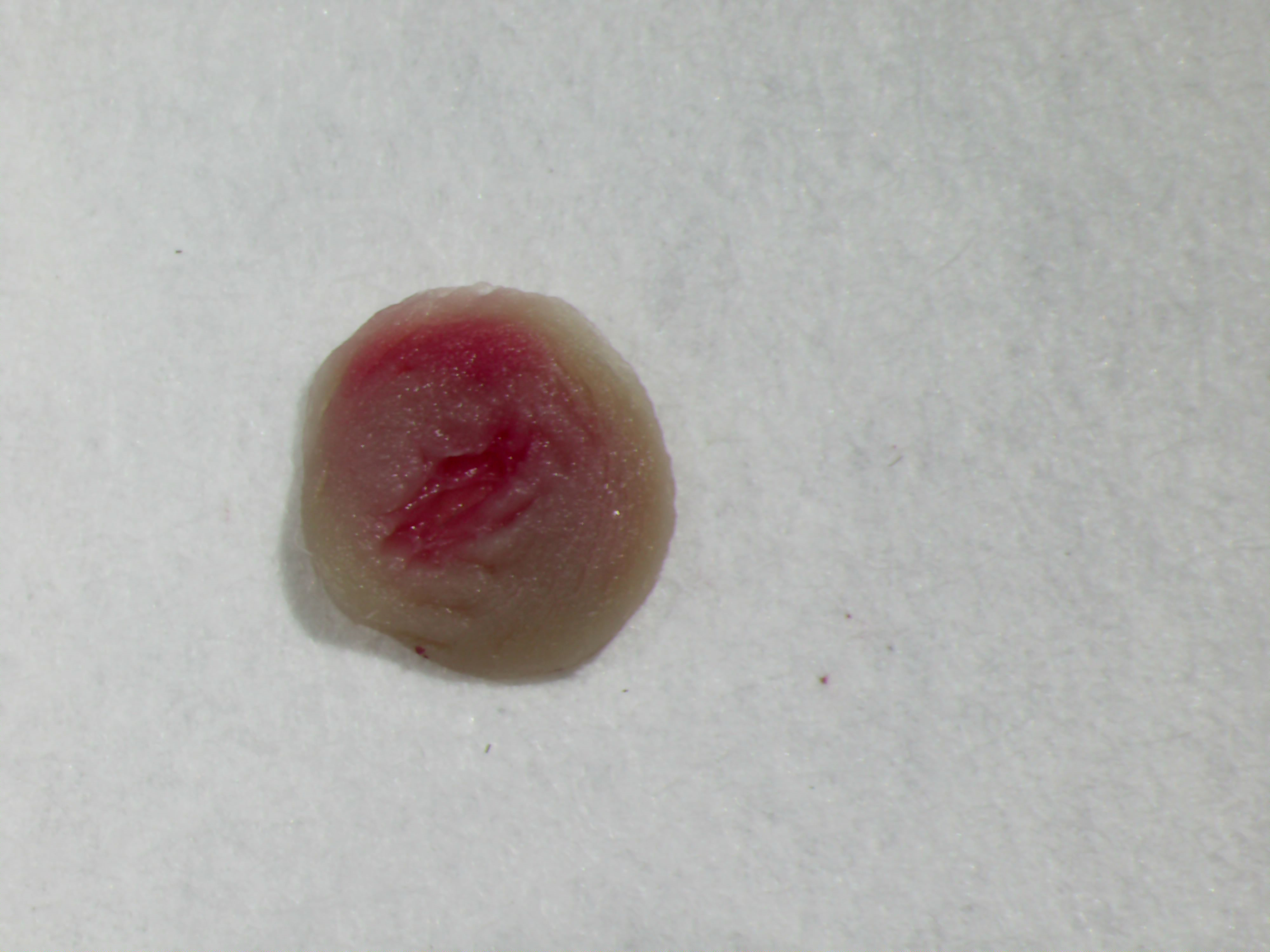

Supplement: S2 File — This zip file (C57.zip) includes the raw experimental materials related to the C57 mouse model. (ZIP) [file pone.0340382.s002.zip › C57/TTC and ultrasound/TTC/IR/2.tiff]

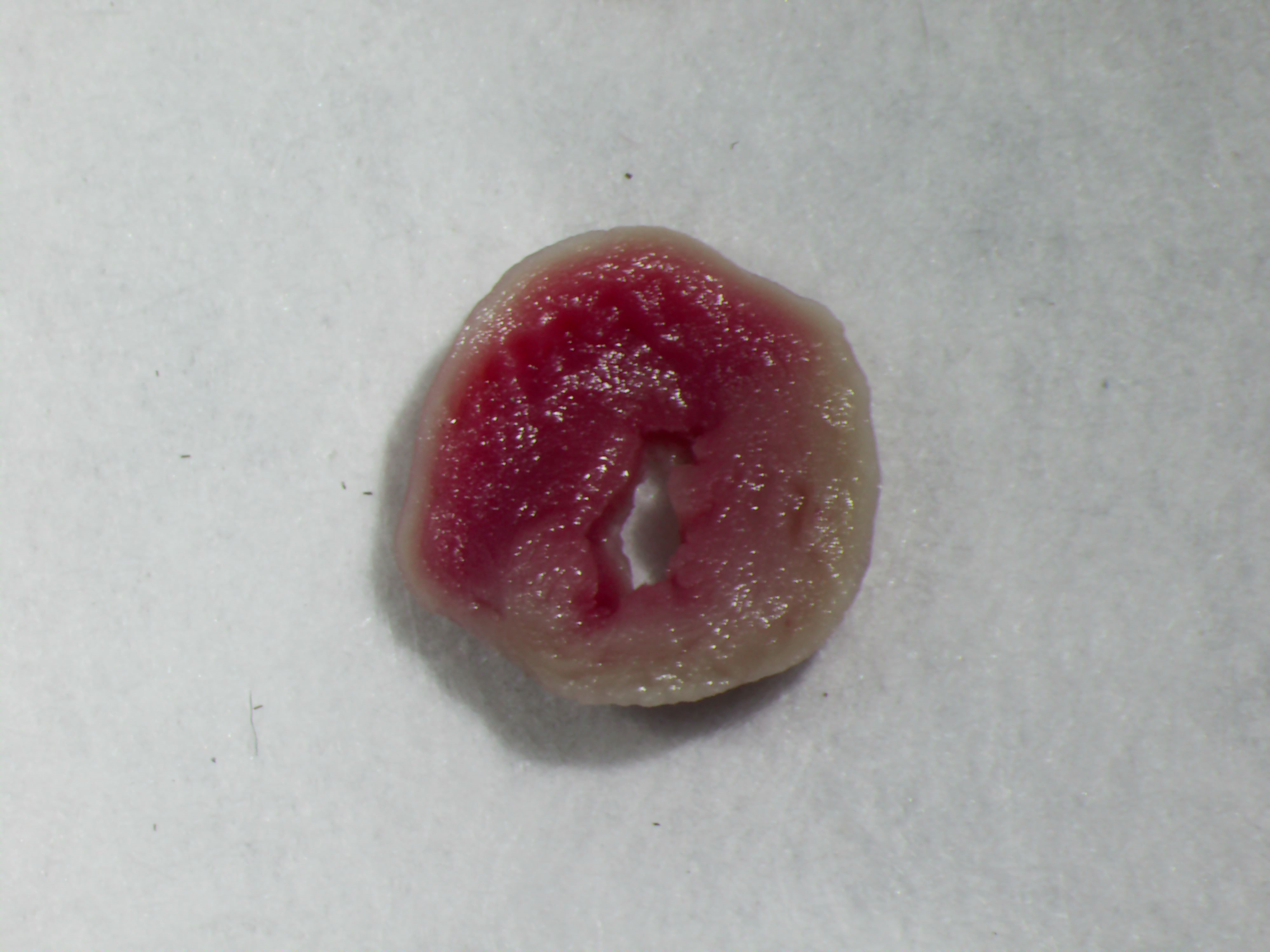

Supplement: S2 File — This zip file (C57.zip) includes the raw experimental materials related to the C57 mouse model. (ZIP) [file pone.0340382.s002.zip › C57/TTC and ultrasound/TTC/IR/3.tiff]

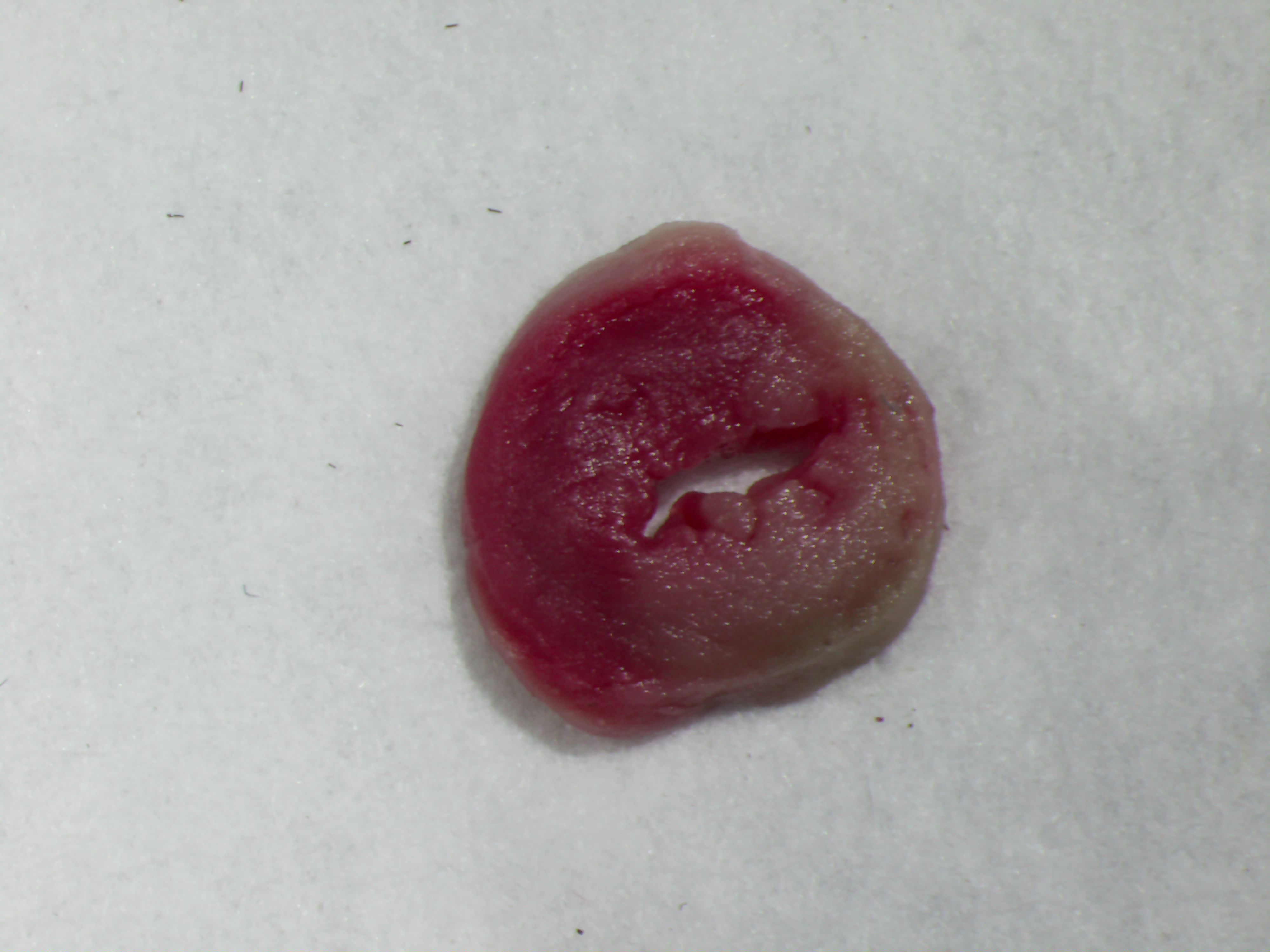

Supplement: S2 File — This zip file (C57.zip) includes the raw experimental materials related to the C57 mouse model. (ZIP) [file pone.0340382.s002.zip › C57/TTC and ultrasound/TTC/IR/4.tiff]

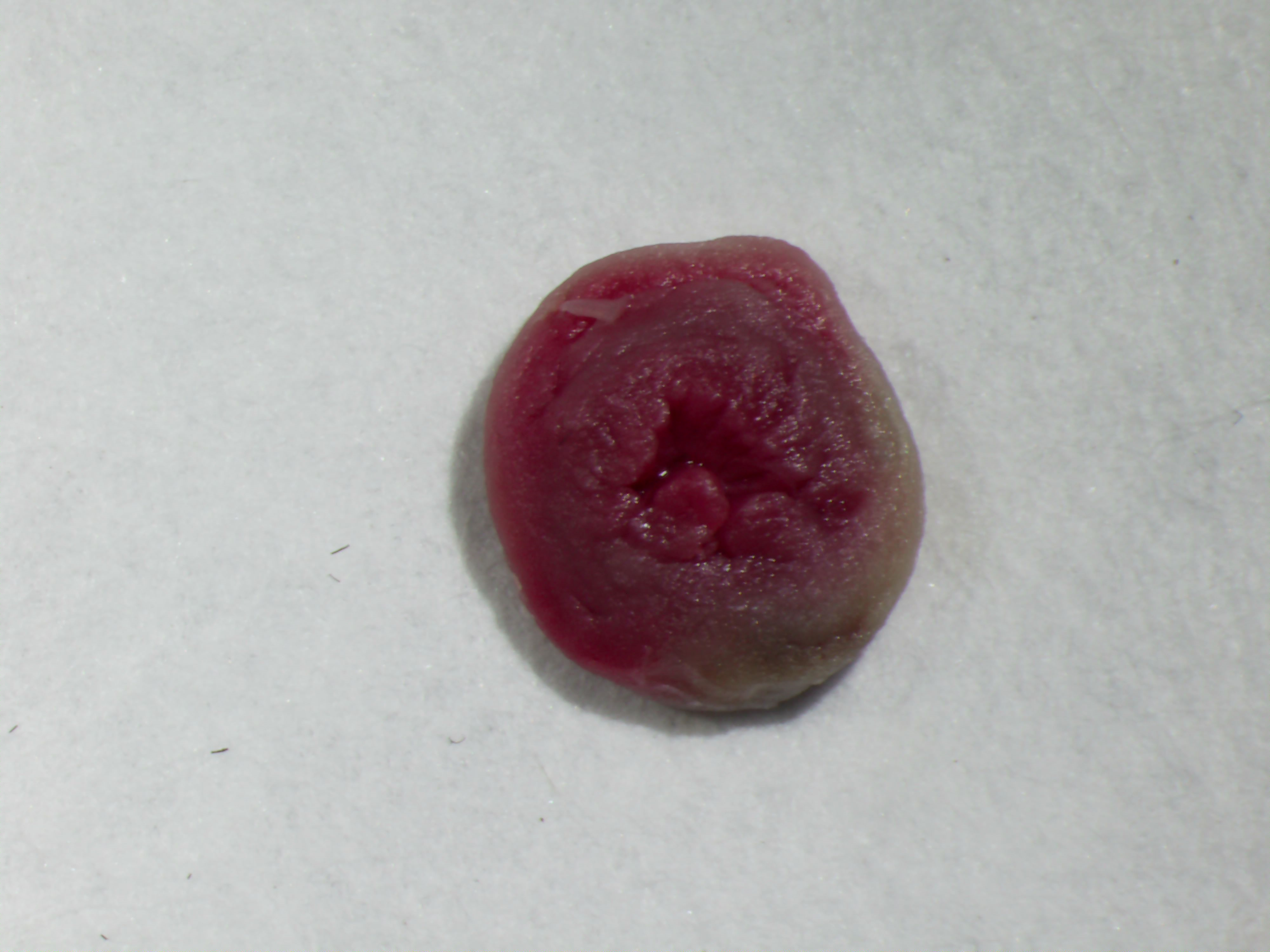

Supplement: S2 File — This zip file (C57.zip) includes the raw experimental materials related to the C57 mouse model. (ZIP) [file pone.0340382.s002.zip › C57/TTC and ultrasound/TTC/IR/5.tiff]

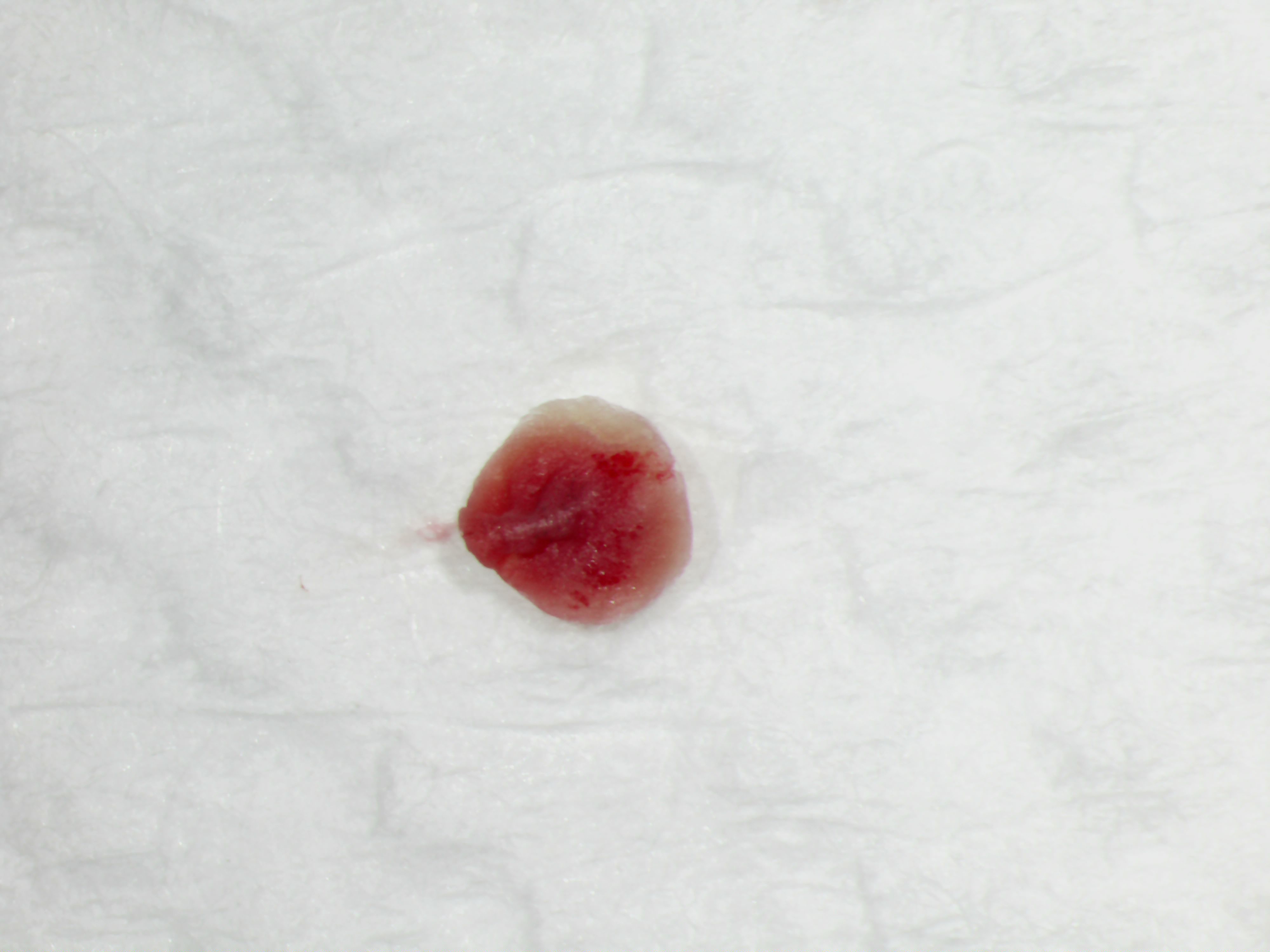

Supplement: S2 File — This zip file (C57.zip) includes the raw experimental materials related to the C57 mouse model. (ZIP) [file pone.0340382.s002.zip › C57/TTC and ultrasound/TTC/Sham/1.tiff]

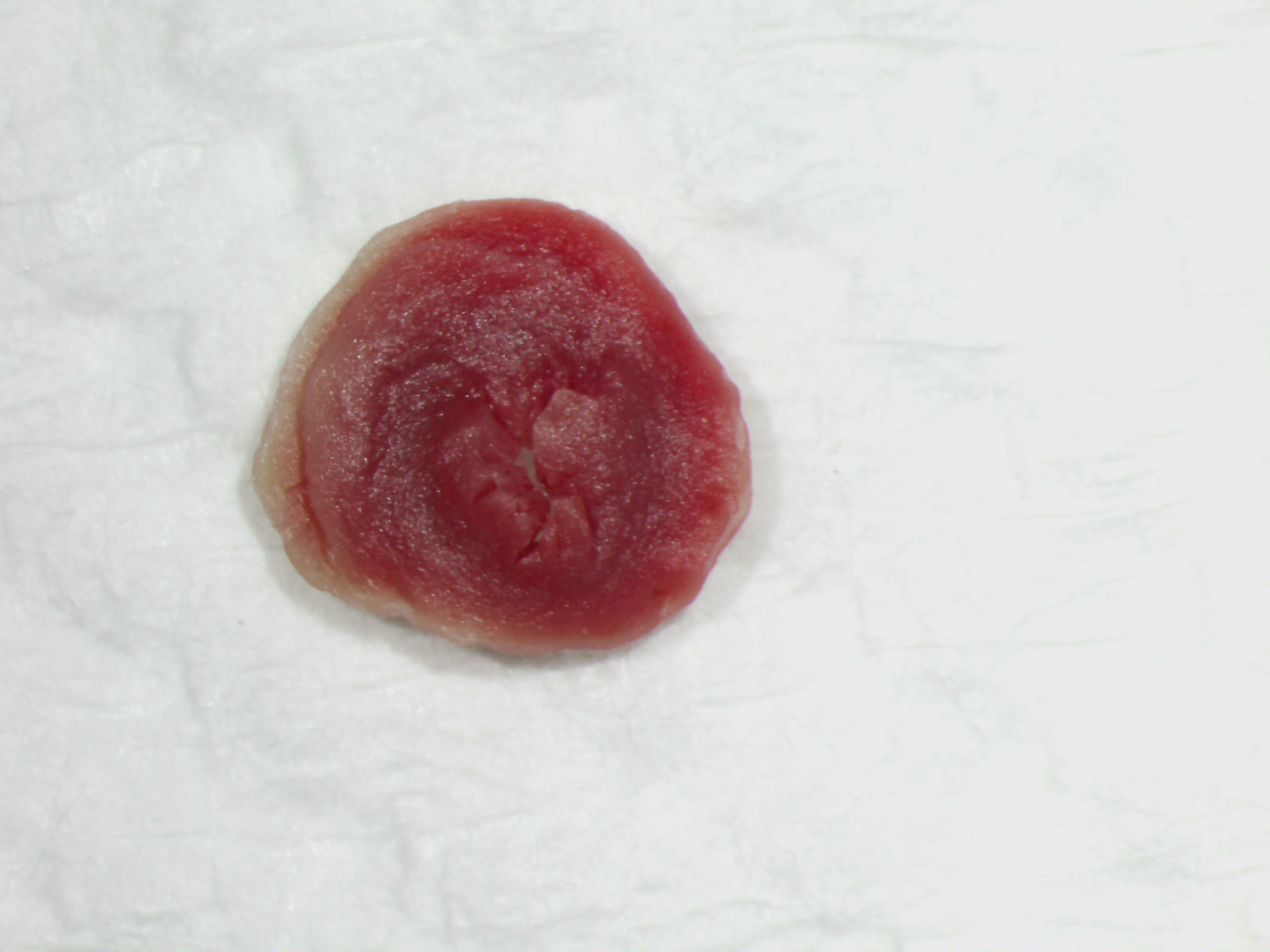

Supplement: S2 File — This zip file (C57.zip) includes the raw experimental materials related to the C57 mouse model. (ZIP) [file pone.0340382.s002.zip › C57/TTC and ultrasound/TTC/Sham/2.tiff]

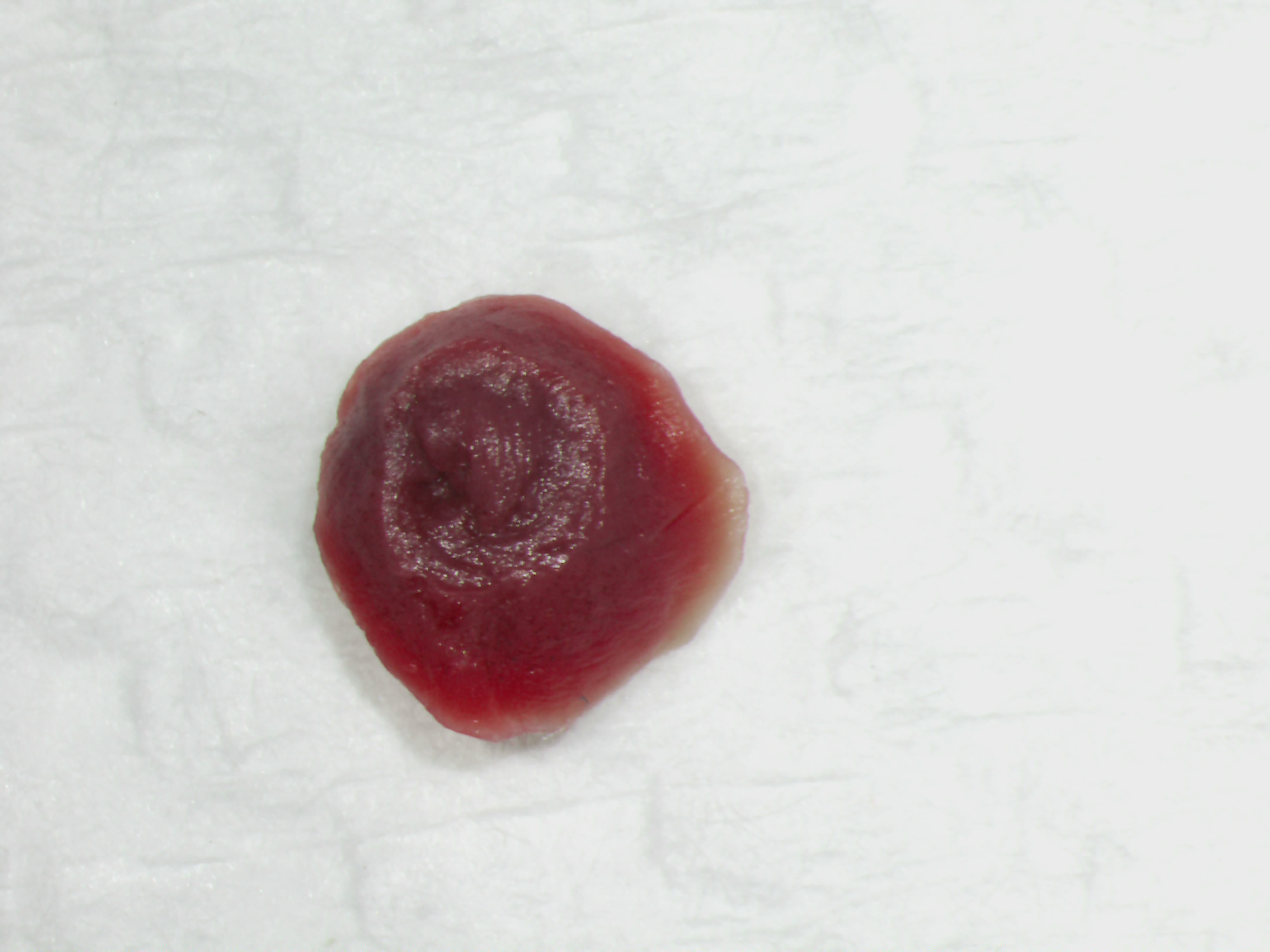

Supplement: S2 File — This zip file (C57.zip) includes the raw experimental materials related to the C57 mouse model. (ZIP) [file pone.0340382.s002.zip › C57/TTC and ultrasound/TTC/Sham/3.tiff]

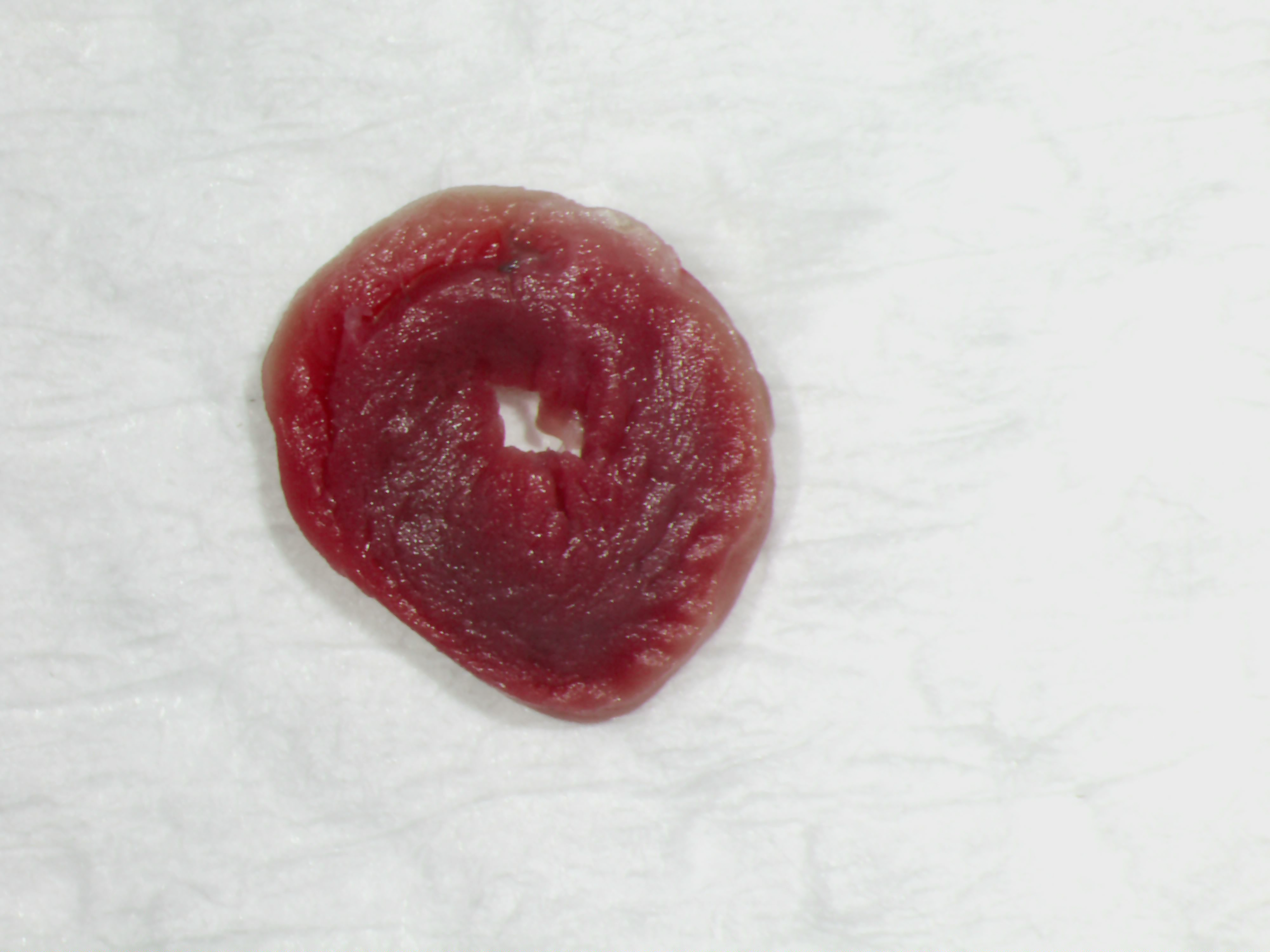

Supplement: S2 File — This zip file (C57.zip) includes the raw experimental materials related to the C57 mouse model. (ZIP) [file pone.0340382.s002.zip › C57/TTC and ultrasound/TTC/Sham/4.tiff]

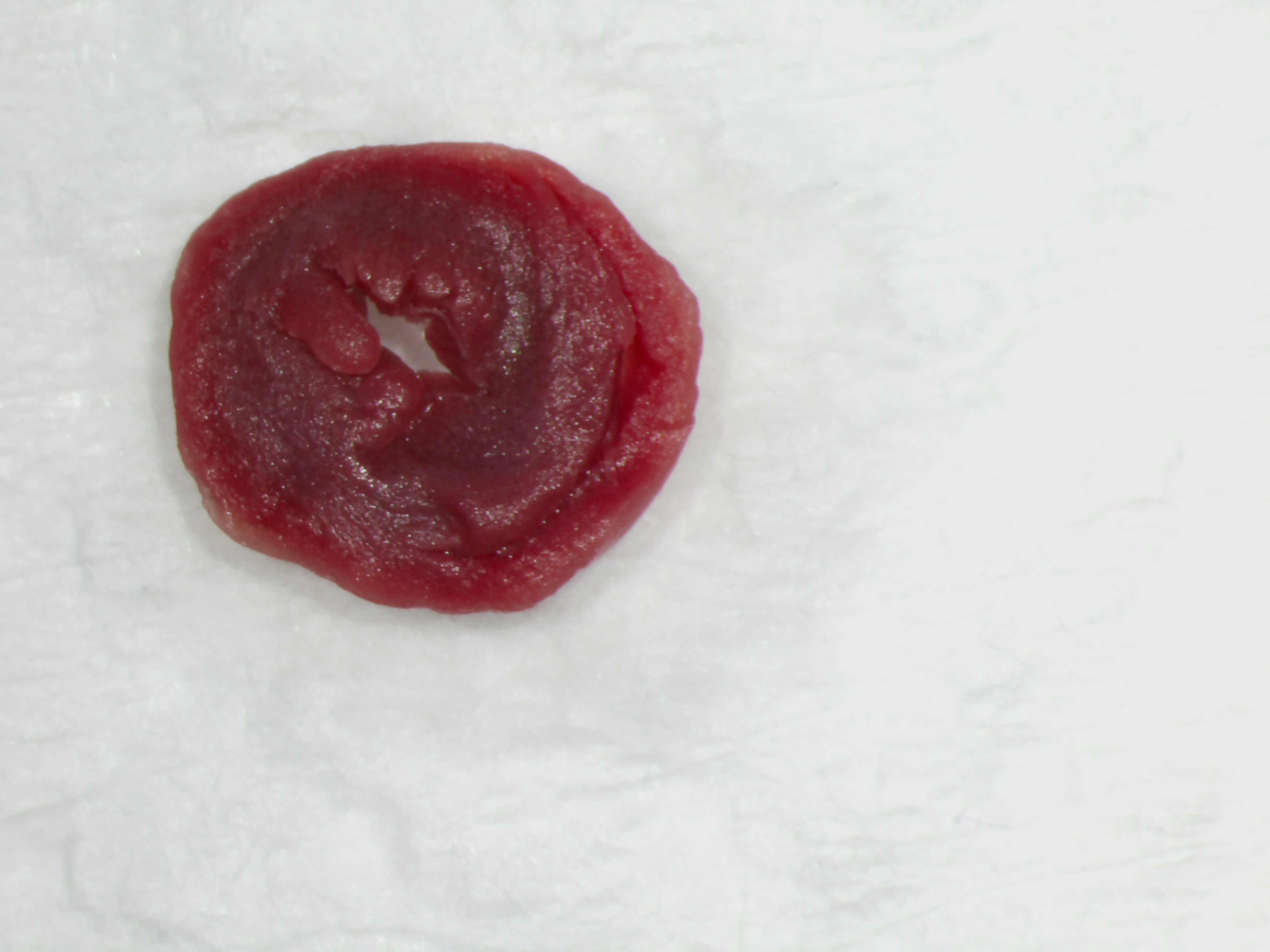

Supplement: S2 File — This zip file (C57.zip) includes the raw experimental materials related to the C57 mouse model. (ZIP) [file pone.0340382.s002.zip › C57/TTC and ultrasound/TTC/Sham/5.tiff]

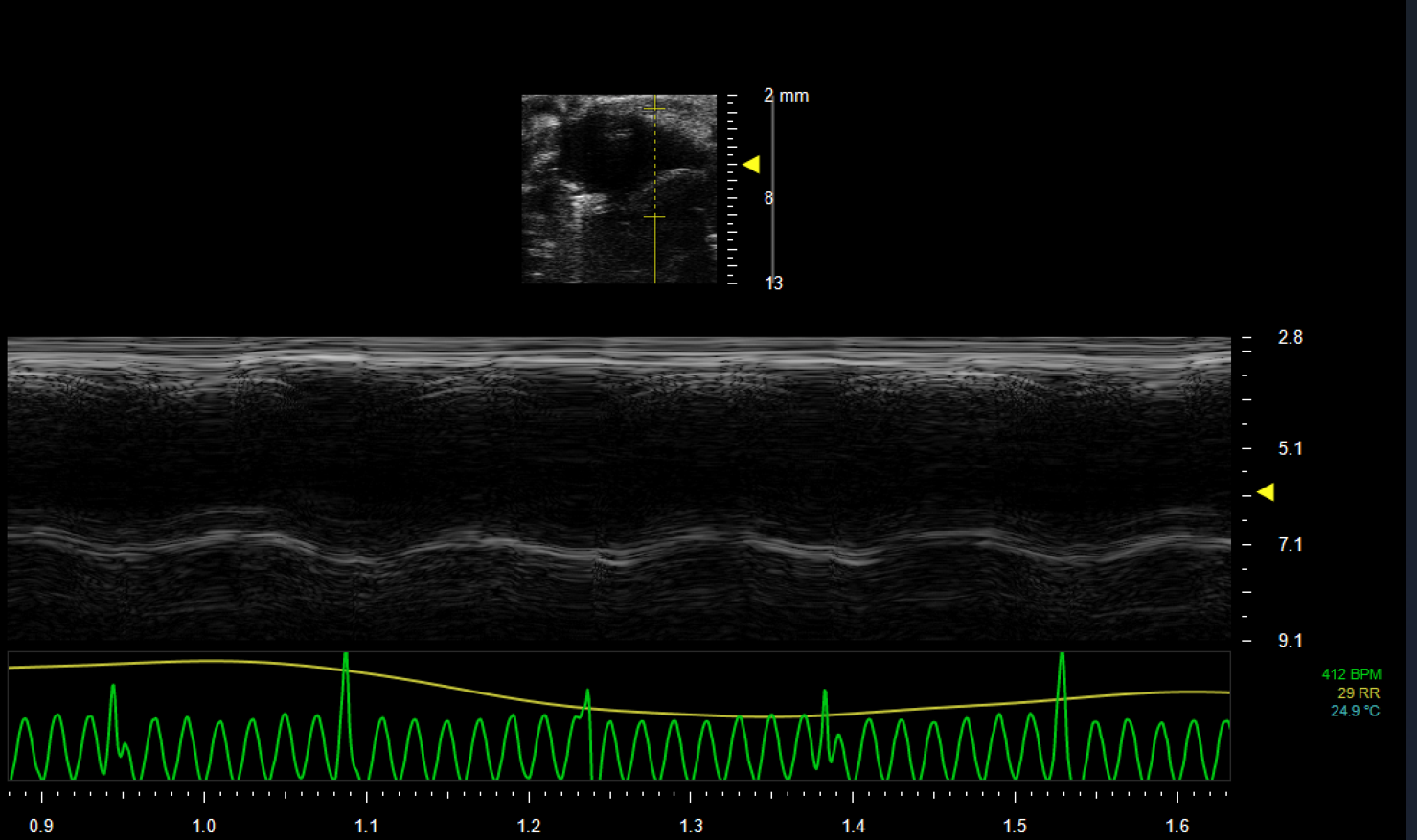

Supplement: S2 File — This zip file (C57.zip) includes the raw experimental materials related to the C57 mouse model. (ZIP) [file pone.0340382.s002.zip › C57/TTC and ultrasound/ultrasound/IR/b210f58a2289480e60454f6f3b42a284.png]

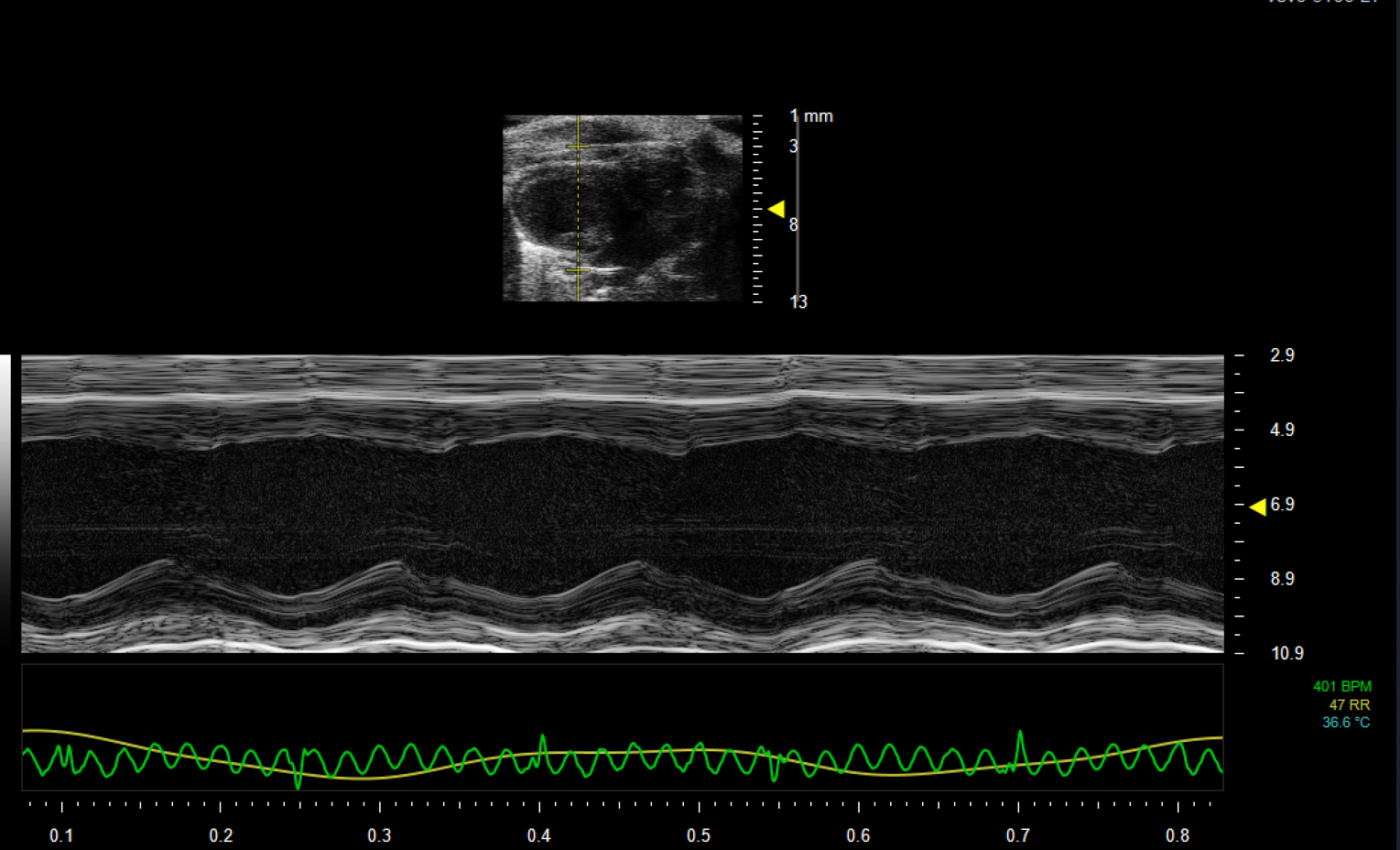

Supplement: S2 File — This zip file (C57.zip) includes the raw experimental materials related to the C57 mouse model. (ZIP) [file pone.0340382.s002.zip › C57/TTC and ultrasound/ultrasound/IR+LR12/611ef0a031ac4d33bb3ba18e1d26b5e5.png]

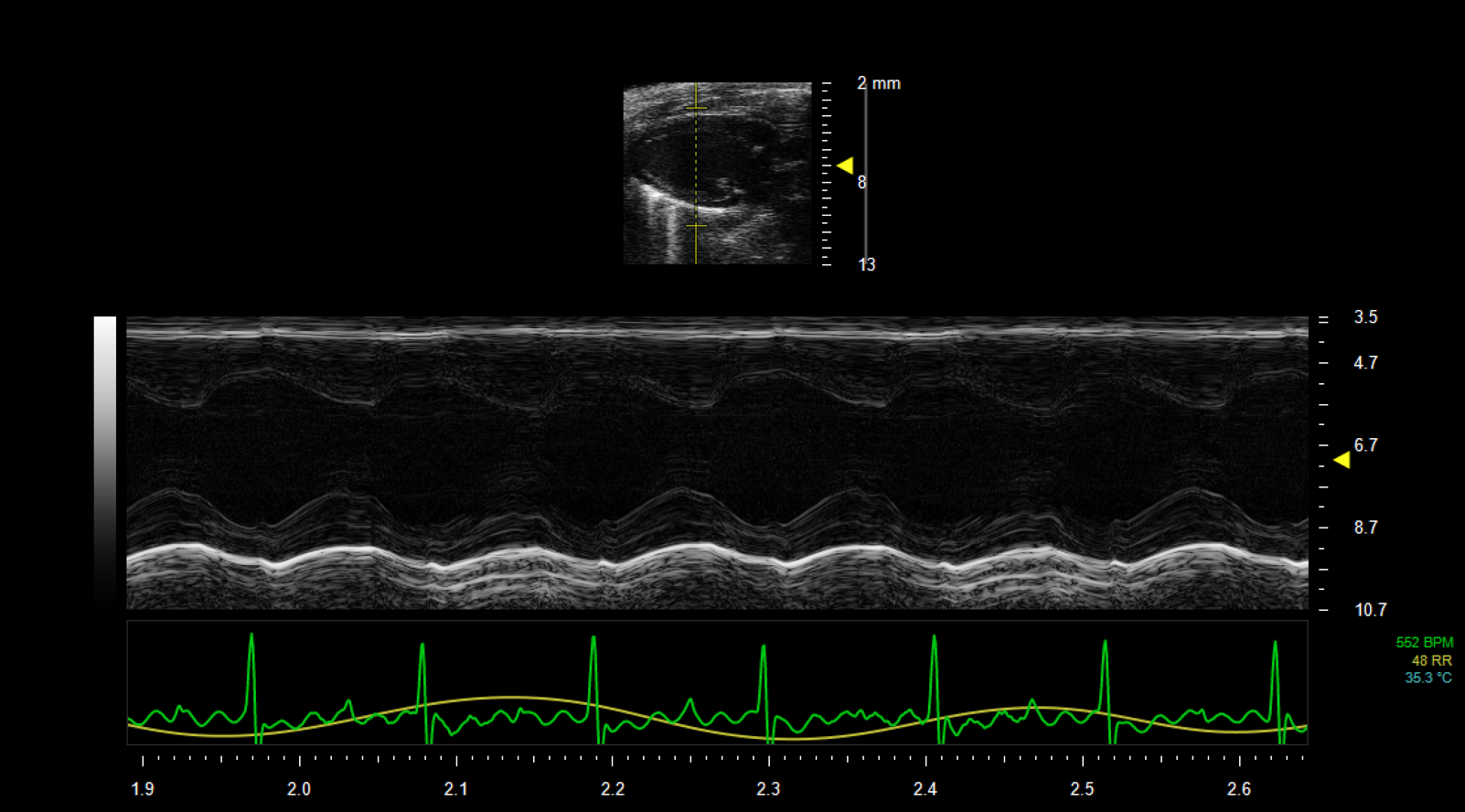

Supplement: S2 File — This zip file (C57.zip) includes the raw experimental materials related to the C57 mouse model. (ZIP) [file pone.0340382.s002.zip › C57/TTC and ultrasound/ultrasound/sham/b67e3414cbbd37fbbde5bb2b266f0bc9.png]

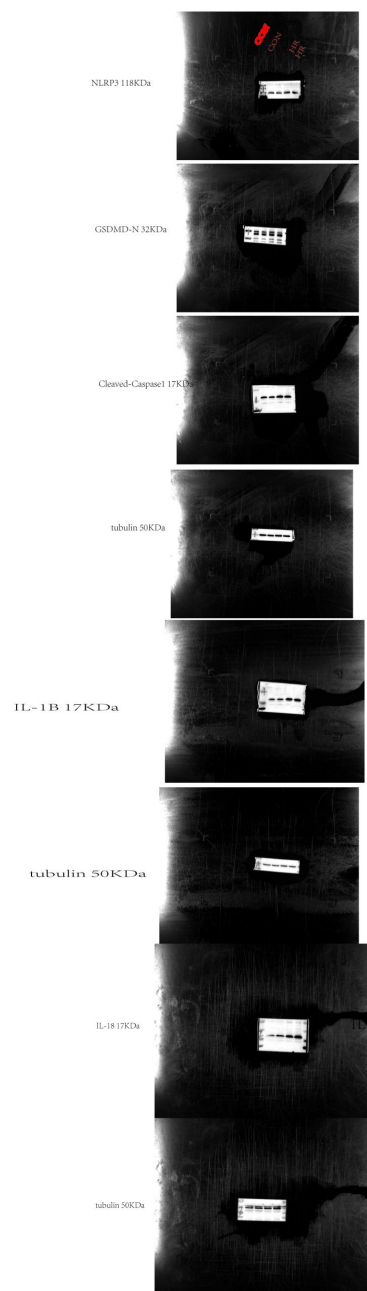

figure1 A-1

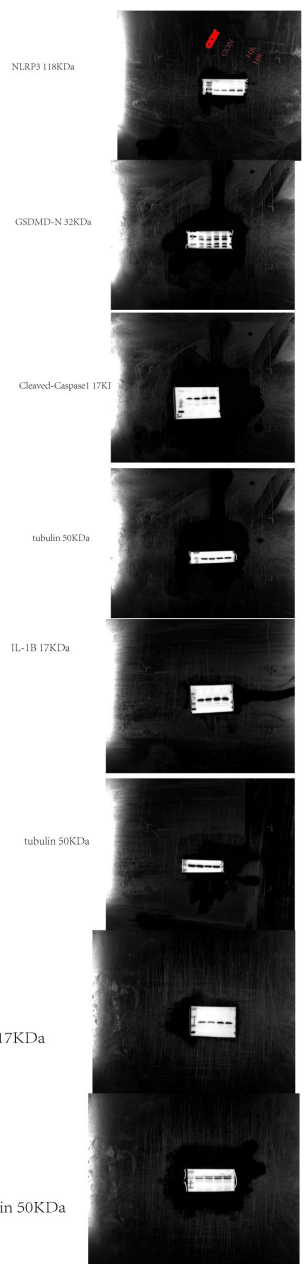

figure1A-2

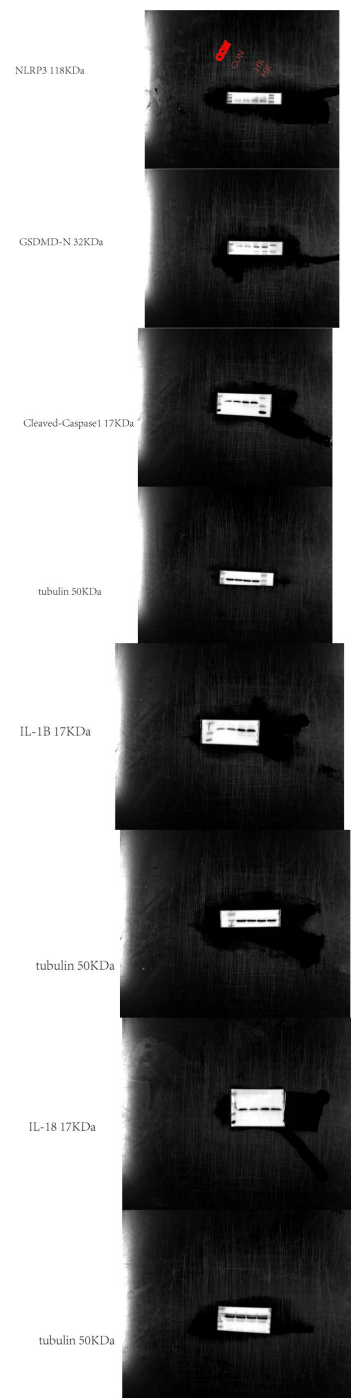

figure1A-3

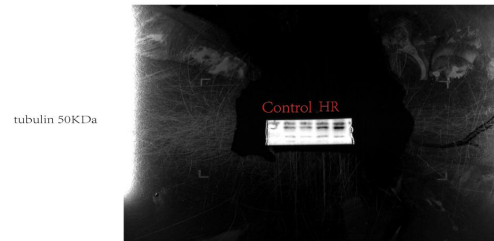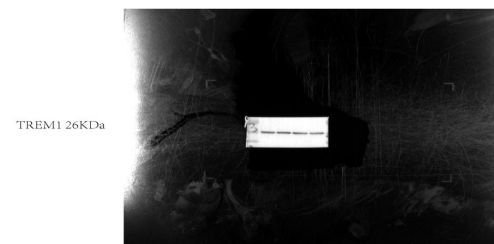

figure1G-1

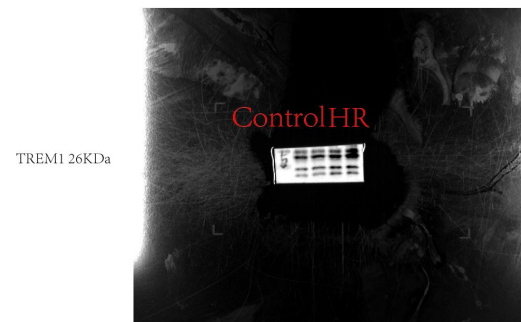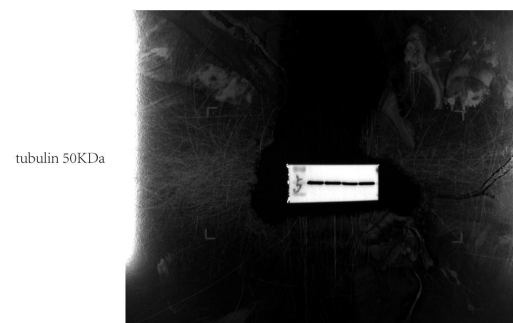

figure1G-2

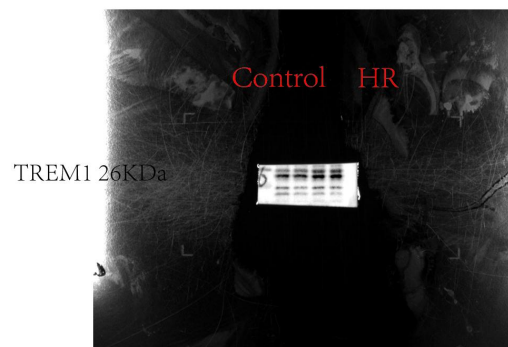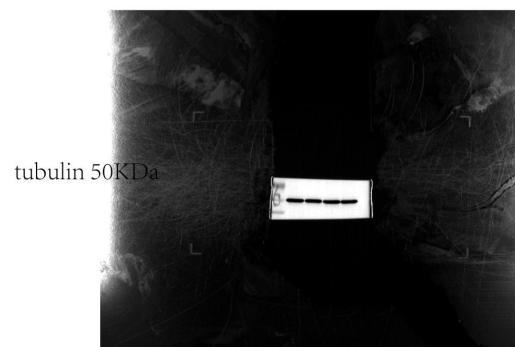

figure1 G-3

Supplement: S4 File — (PDF) [file pone.0340382.s004.pdf]

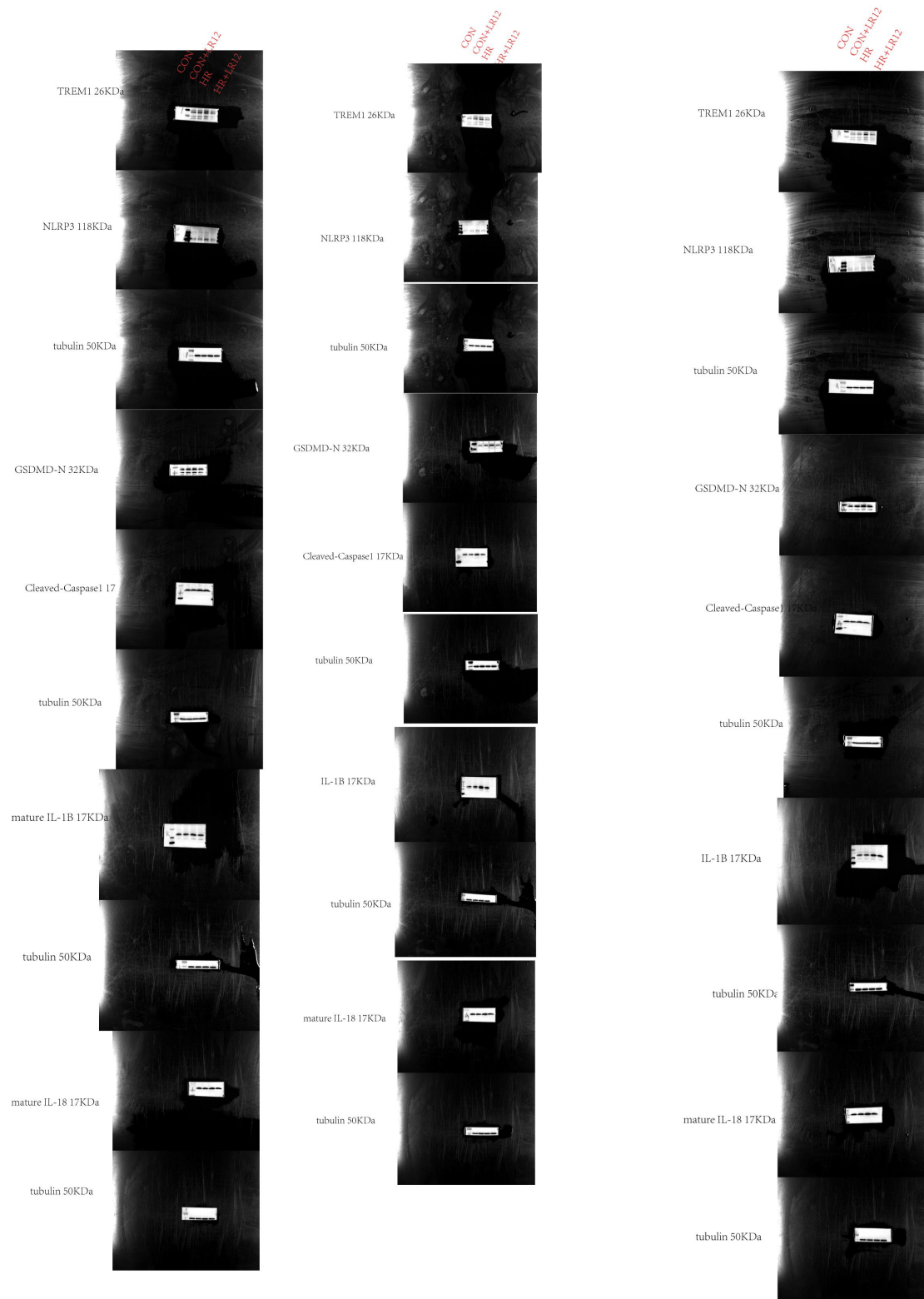

figure2 E

Supplement: S5 File — (PDF) [file pone.0340382.s005.pdf]

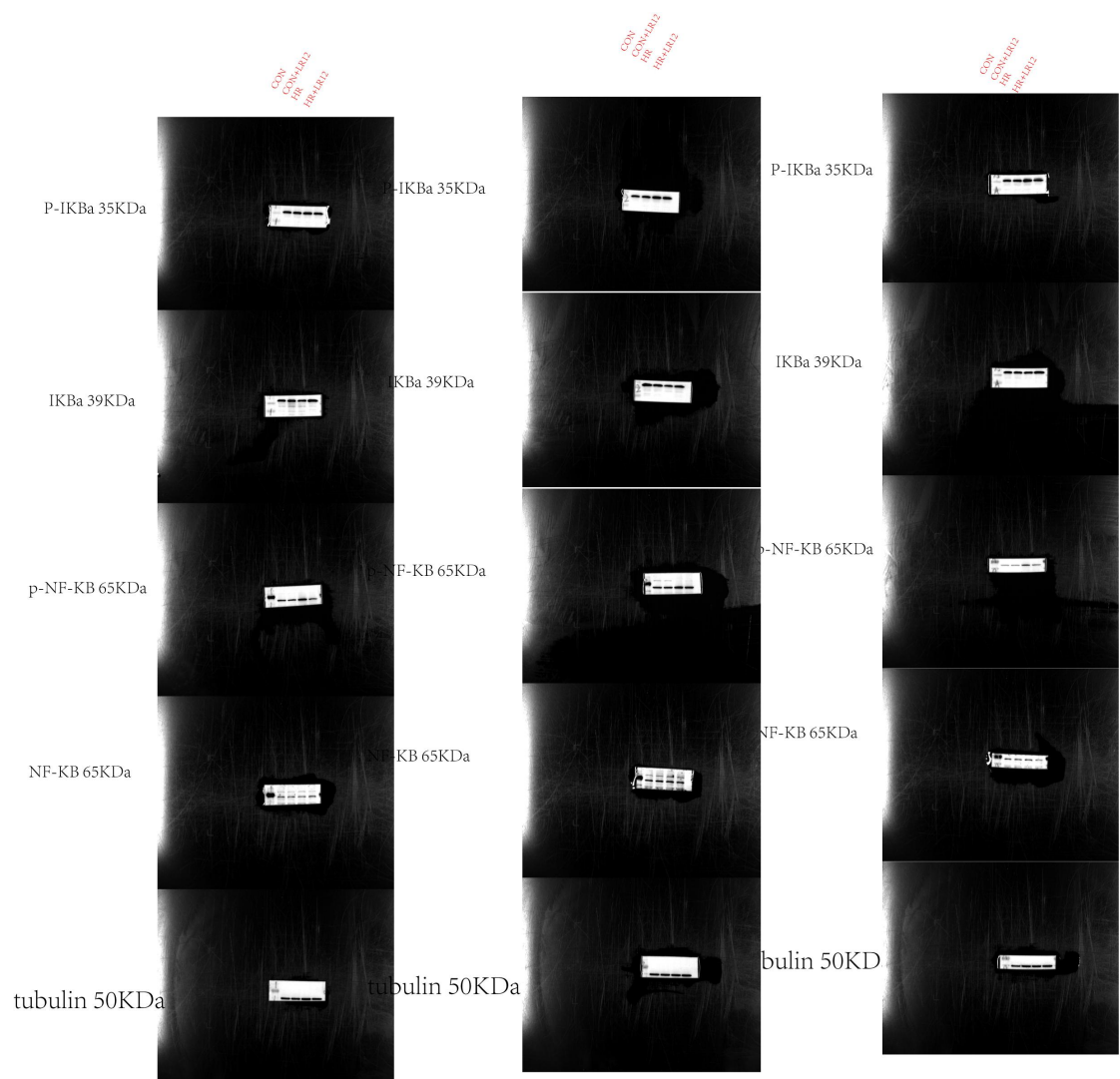

figure3A

Supplement: S6 File — (PDF) [file pone.0340382.s006.pdf]

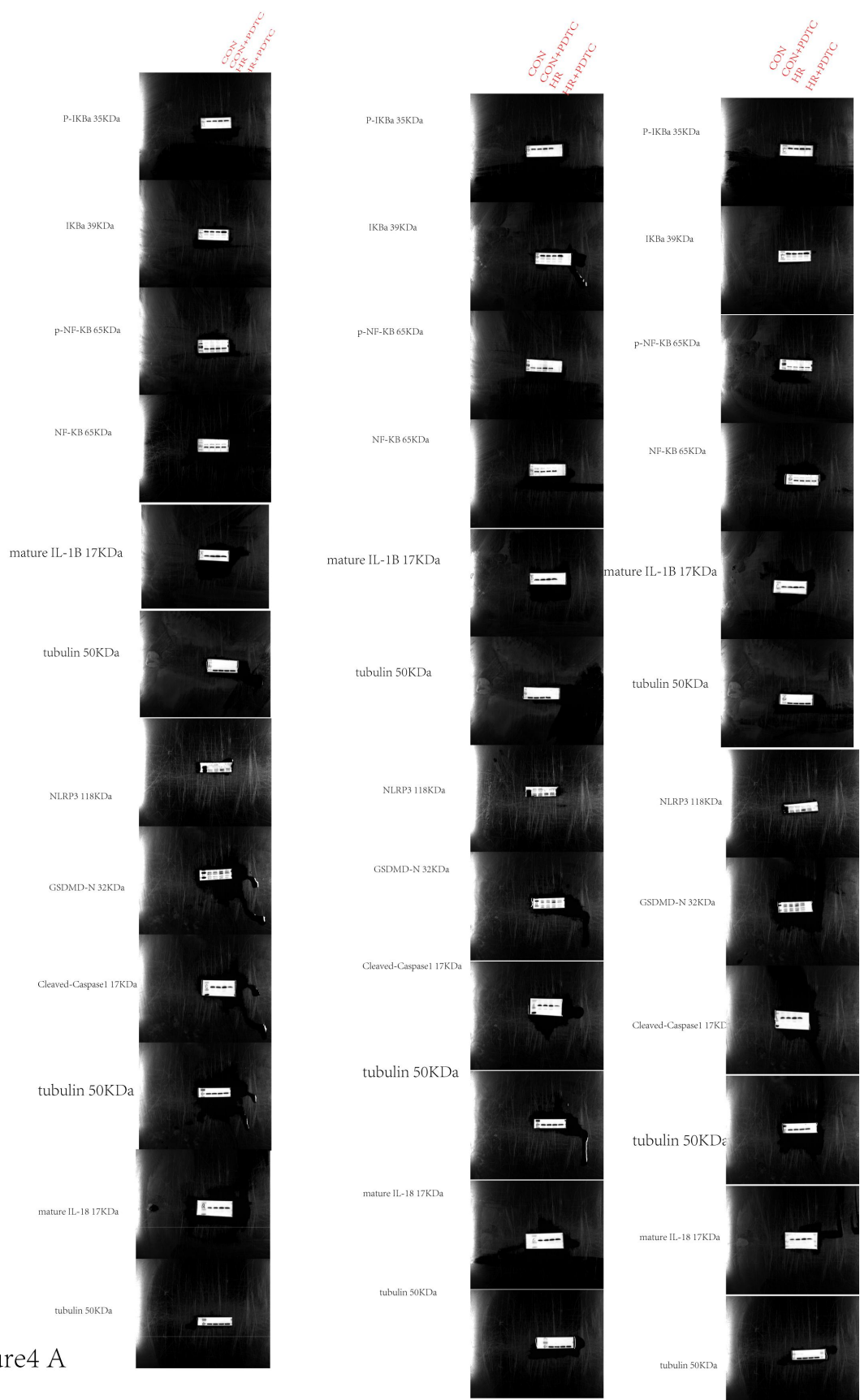

figure4 A

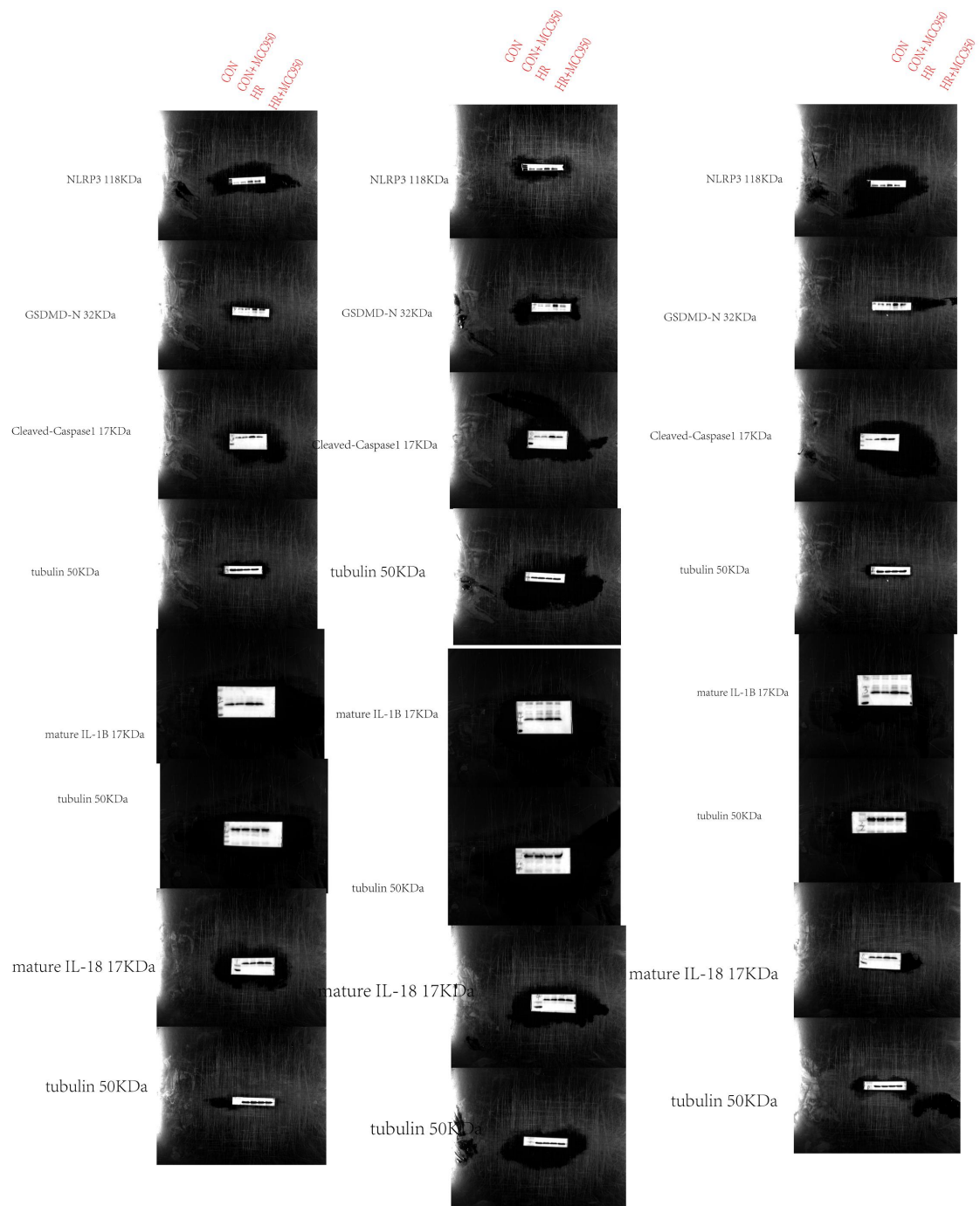

figure4 I

Supplement: S7 File — (PDF) [file pone.0340382.s007.pdf]

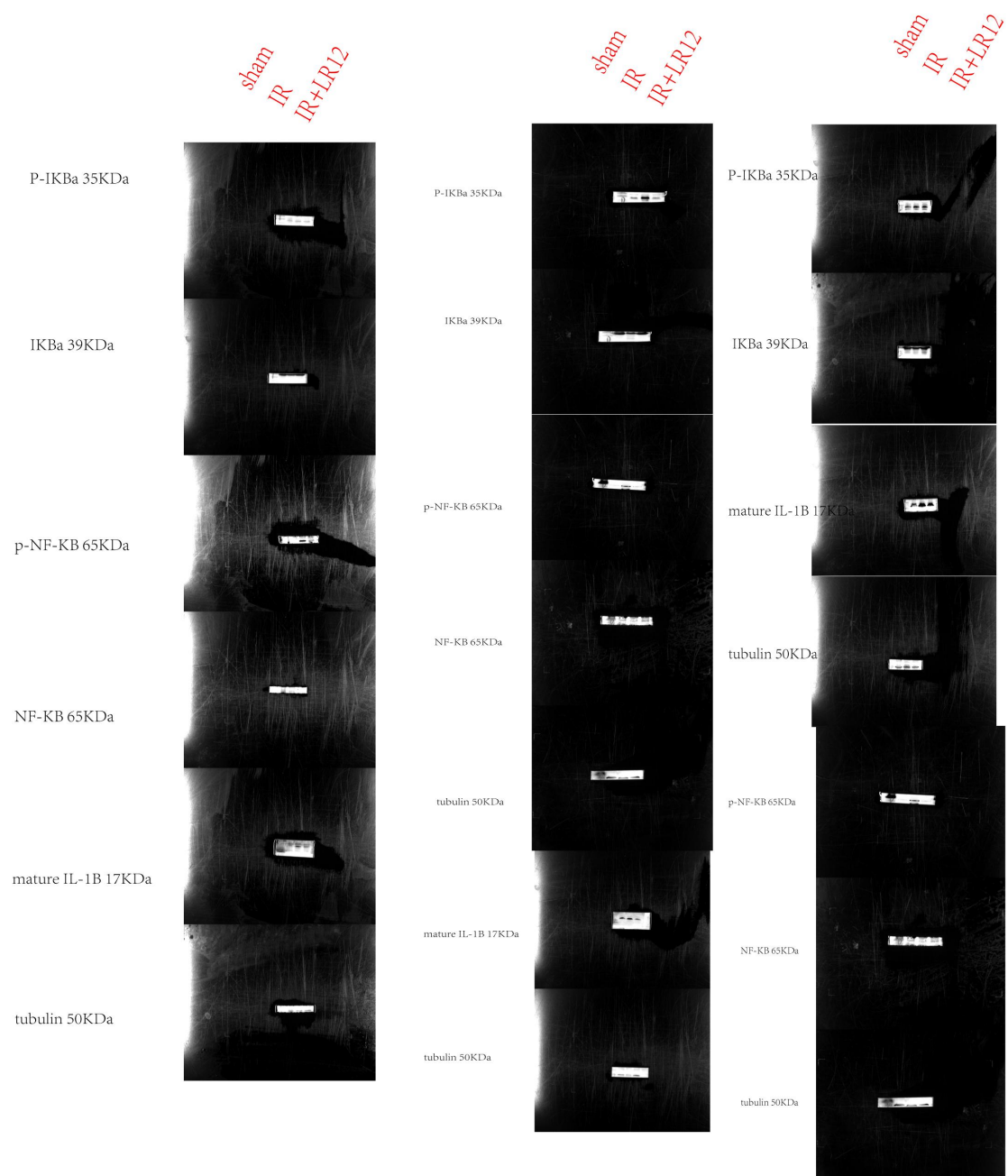

figure5A

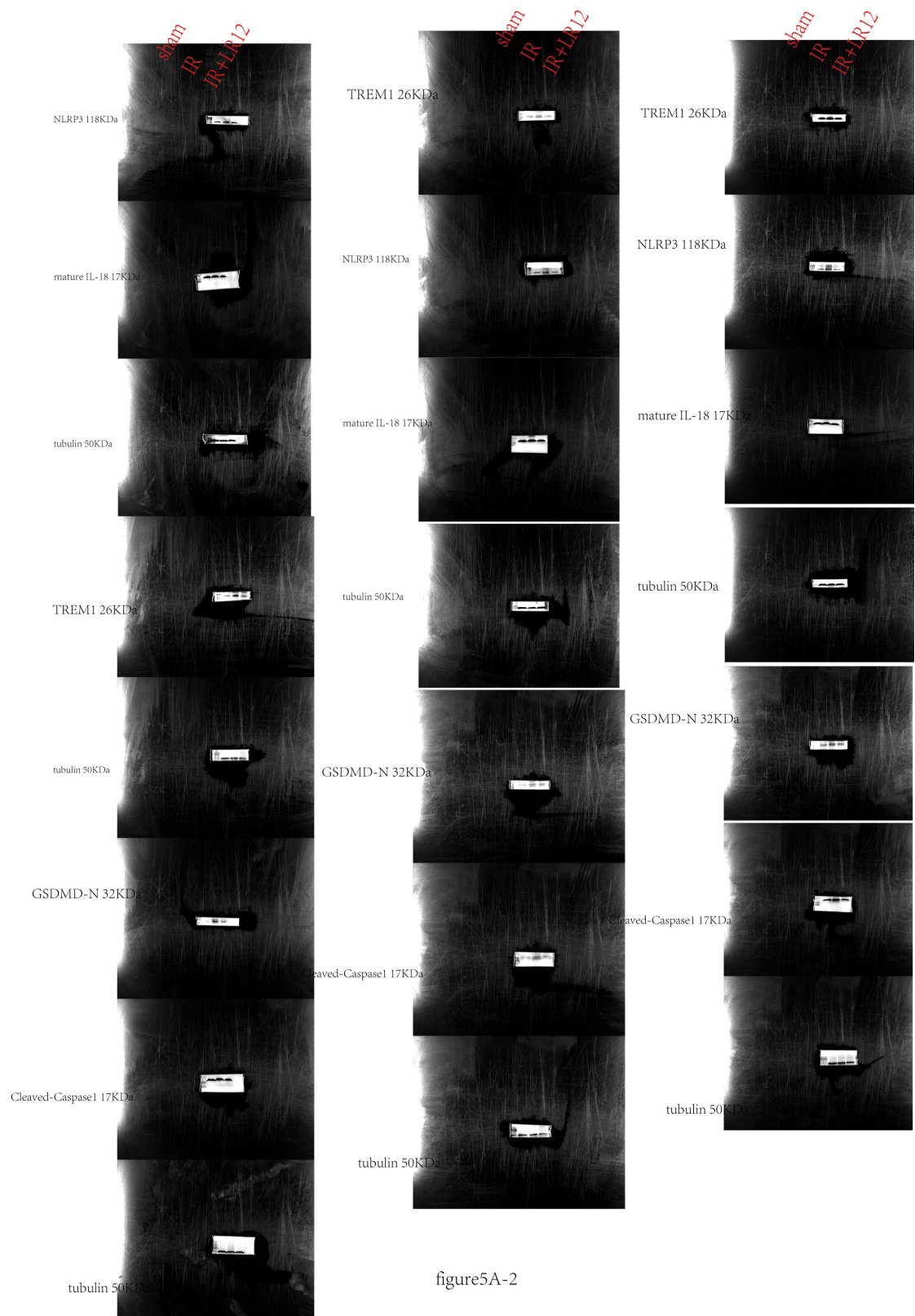

figure5A-2

Supplement: S8 File — (PDF) [file pone.0340382.s008.pdf]
